# Investigating relationships between loneliness, social isolation and health

**DOI:** 10.1038/s41467-026-74758-7

**Published:** 2026-07-15

**Authors:** Darren D. Hilliard, Robyn E. Wootton, Hannah M. Sallis, Margot P. Van De Weijer, Jorien L. Treur, Pamela Qualter, Padraig Dixon, Eleanor C. M. Sanderson, David J. Carslake, Rebecca C. Richmond, Patricia Beloe, Lucy Turner-Harris, Lauren Bowes Byatt, Marcus R. Munafò, Zoe E. Reed

**Affiliations:** 1 Nesta, Temple, London, UK; 2 School of Psychological Science, University of Bristol, Bristol, UK; 3 MRC Integrative Epidemiology Unit at the University of Bristol, Bristol, UK; 4 Psychiatric Genetic Epidemiology Group, Research Department, Lovisenberg Diaconal Hospital, Oslo, Norway; 5 PsychGen Centre for Genetic Epidemiology and Mental Health, Norwegian Institute of Public Health, Oslo, Norway; 6 Population Health Sciences, Bristol Medical School, University of Bristol, Bristol, UK; 7 Centre for Academic Mental Health, Bristol Medical School, University of Bristol, Bristol, UK; 8 Genetic Epidemiology, Department of Psychiatry, Amsterdam UMC location University of Amsterdam, Amsterdam, The Netherlands; 9 Manchester Institute of Education, University of Manchester, Manchester, UK; 10 Nuffield Department of Primary Care Health Sciences, University of Oxford, Oxford, UK; 11 Vice Chancellor’s Office, University of Bath, Bath, UK

**Keywords:** Risk factors, Behavioural genetics

## Abstract

Loneliness and social isolation are important public health concerns due to their associations with a range of health outcomes. However, it is difficult to ascertain whether any effects are biased by confounding and reverse causation. We use a triangulation approach combining observational, sibling control, and Mendelian Randomisation analyses (a genetically informed analysis), to draw robust conclusions about these relationships. Using a combination of UK Biobank data (N = 8,004 to 414,432) and genome-wide association studies (N = 17,526 to 2,083,151), we examine relationships between loneliness and social isolation and outcomes related to physical health, mental health and wellbeing and general health (e.g., multimorbidity capturing both mental and physical health). We find evidence for effects of loneliness and social isolation on poorer mental health and wellbeing and of loneliness on poorer general health. We do not find evidence of effects on specific physical health outcomes; however, these effects cannot definitively be ruled out.

## Abstract (web summary)

Loneliness and social isolation are associated with a range of health outcomes. Here, the authors find evidence of effects of loneliness and social isolation on poorer mental health and wellbeing, and effects of loneliness on poorer general health.

## Introduction

Loneliness and social isolation have become important public health issues in recent years, both being associated with poorer health outcomes, including specific physical and mental health outcomes (e.g., cardiovascular disease, depression, anxiety, and wellbeing), and general health outcomes (which capture both physical and mental health e.g., mortality)^1–8^. Loneliness and social isolation are related concepts, but differ in how they are defined and measured. Loneliness is a subjective measure of how individuals feel about their relationships (i.e., perceived social connectedness); social isolation is an objective measure of the number and type of social connections an individual has^9^.

There have been numerous studies examining the relationships between loneliness and/or social isolation, and health which have predominantly included observational analyses using cross-sectional data, demonstrating that those associations exist^4,8,10–13^. We note that there are few studies that have examined the effects of both loneliness and social isolation on health and therefore it is difficult to disentangle whether they have different effects on health outcomes. Where these have been studied together^3^ they have influenced similar outcomes, although often to different extents^1^. However, the nature of these relationships may be different and there is a need to investigate whether influences of loneliness and social isolation on health outcomes are independent. There is also longitudinal evidence suggesting loneliness and social isolation may influence later health outcomes^1,3,14^. Previous studies suggest that associations between loneliness and social isolation and health may be large. For example, loneliness was found to be associated with a 2.3 times higher likelihood of depression^7^ and social isolation was found to be associated with a 1.5 times higher risk of coronary artery disease^15^. However, establishing whether these associations reflect potentially causal relationships, and the direction of causation, can be difficult. Most previous studies have been unable to address this because they may be subject to residual confounding of the relationships between loneliness/social isolation and health outcomes, and reverse causation^3^.

One approach that can overcome the issues of confounding and reverse causation, is Mendelian Randomisation (MR)^16–19^, a genetically informed approach. MR uses genetic variants (single nucleotide polymorphisms; SNPs) as instrumental variables (IVs) for an exposure of interest (e.g., loneliness and social isolation) to estimate effects on outcomes of interest (providing assumptions are satisfied^19^). Whilst there have been many previous observational and longitudinal studies in this area of research, there have been few MR studies, and even fewer studies that examine these relationships using MR alongside other approaches with different strengths and biases. Of those MR studies that have been conducted, some report evidence of effects of loneliness/social isolation on increased risk of various health outcomes, predominantly for mental health outcomes^20–22^. Other MR studies have not found evidence of effects of loneliness/social isolation on both physical and mental health outcomes^23–25^, reflecting mixed findings in this area. These mixed MR findings may be a result of differences in the exact exposures used (i.e., examining leisure activities^22^ versus frequency of social contacts^26^ with both being described as social isolation measures, or combining measures of loneliness and social isolation into a single measure making it difficult to disentangle specific effects^23,25^) or differences in the approaches taken. For example, some previous studies have used a wide range of sensitivity analyses and found less consistent results across these suggesting a lack of evidence of effects^24,27^, whilst others have included minimal sensitivity analyses^25^, or have focused largely on results from the main analyses^20,22^. This has meant the way in which results have been interpreted has varied. In addition, many of those studies have further limitations in terms of using less stringent p-value thresholds for instrument selection^20,22^, not including appropriate sensitivity analyses or not interpreting results appropriately in light of these^20,22,25^. A more recent MR study, which also included observational analyses, found evidence of effects of loneliness on only 6 of the 26 physical and mental health outcomes examined (hypothyroidism, asthma, sleep apnoea, depression, psychoactive substance abuse and hearing loss)^27^. In the current study we have built on previous MR analyses, in particular the most recent aforementioned MR study by: (1) including both loneliness and social isolation as exposures, (2) using more specific measures of these (previous measures have captured multiple related concepts making it difficult to disentangle specific effects), (3) including a wider number of relevant outcomes (e.g., including continuous measures and novel outcomes in this area such as rates of hospital admissions and quality-adjusted life years), (4) using a greater range of sensitivity analyses, and (5) conducting MR alongside other analyses to strengthen causal inference. These aspects are particularly important given that loneliness and social isolation are complex phenotypes and the genetic variants proxying for these may violate key assumptions of MR. However, caution should still be taken when interpreting MR results using exposures like loneliness and social isolation that are more biologically distal from genetic instruments, and where these are less well defined than more biological exposures would be. Therefore, it is important to consider findings from MR analyses alongside findings from other approaches.

Within-family analyses can also help to estimate effects of an exposure on an outcome, whilst controlling for familial confounding and shared genetic predispositions. This approach requires the use of cohort data where there are siblings or other family members. Given this limitation there are few studies using within-family approaches to investigate relationships between loneliness/social isolation and health^28–30^. Of those that do exist they are focused on specific health-related outcomes and there are no studies, to our knowledge, looking at a range of health outcomes using within-family approaches.

In the current work we investigated specific health outcomes that are considered to have the greatest global ‘burden’^31,32^ and that have, in previous studies, been found to be associated with loneliness and/or social isolation. We categorised these into three broad categories of general health, physical health, and mental health and wellbeing. We employed a triangulation approach^33,34^ that consisted of three parts: (1) observational analyses to establish whether associations exist between loneliness or social isolation and the health outcomes, adjusting for important potential confounders; (2) sibling control analyses to facilitate control for familial confounding (e.g., parental socioeconomic status) and shared genetic predispositions; and (3) MR analyses to estimate effects, reducing bias from confounding and reverse causation. These analyses, in combination, provide evidence as to whether the associations reflect potential causal effects. Consistent results across all three analyses, which each have different strengths and avoid different sources of bias, enables us to draw more robust conclusions about the relationships between loneliness/social isolation and health outcomes^33^. This approach extends previous research in this area by using a pre-registered triangulation approach, including a comprehensive number of sensitivity analyses and examining a range of outcomes.

## Results

We combined observational analyses (cross-sectional and some with outcomes at a later timepoint, including time-to-event analyses), sibling control analyses, bidirectional two-sample MR (2SMR)^16,35^ and one-sample MR (1SMR)^36^ analyses in our triangulation approach. We used this to investigate the relationships between both loneliness and social isolation and a range of physical, general, and mental health outcomes, with the caveat that some outcomes were not available for either the 1SMR approach (as individual data was needed) or the 2SMR approach (as genome-wide association study (GWAS) data was needed). The sibling control analyses included two estimates: between- and within-family effects. The within-family effect provided a less biased estimate as it accounts for confounding factors shared within families. We used a combination of publicly available GWAS data for 2SMR and for the exposure in 1SMR, and data from UK Biobank for observational, sibling control and 1SMR analyses (see Methods for details). We also conducted multivariable MR (MVMR) to examine the independent effects of loneliness and social isolation on health.

The loneliness measure in UK Biobank captured whether the participant often felt lonely, and in the GWAS captured a combination of this same measure and other composite measures of loneliness (Supplementary Table S1). The social isolation measure in UK Biobank and the GWAS we conducted was a continuous variable combining frequency of friend/family visits and the number of people living in their household. The final measure had values between 0 and 2, where 2 indicated greater social isolation. We examined outcomes related to general health (hospital admissions^37^, quality-adjusted life years [QALYs]^38,39^, multimorbidity and mortality/death^40,41^)^12^, physical health (cardiovascular outcomes including coronary artery disease, heart failure^42,43^, stroke, systolic blood pressure^44,45^ type 2 diabetes^46,47^) and mental health^7^ (self-harm^48^, suicidal attempts^49^, depression diagnosis, a continuous depression trait^50^, anxiety diagnosis and a continuous anxiety trait^51^); wellbeing was captured by positive affect/happiness, meaning in life, a spectrum of wellbeing traits and life satisfaction^1^) – see Methods for details.

### UK Biobank sample description

A description of the UK Biobank data used in our analyses is presented in Table 1.

**Table 1:** UK Biobank sample description

Phenotype	*N*	Mean (SD) or percentage (%)
Age	422,899	56.45 (8.10) years
Sex	422,899	Female: 54%
Townsend deprivation index	422,356	−1.28 (3.11)
Years of education	401,953	14.99 (5.21)
Household income before tax	357,567	Less than £18,000: 22% £18,000 to £30,999: 25% £31,000 to £51,999: 26% £52,000 to £100,000: 21% Greater than £100,000: 6%
Ethnicity (self-reported responses)	420,389	White: 94% Asian or Asian British (Indian, Pakistani, Bangladeshi): 2% Black or Black British: 2% Mixed or any other Asian background, or Chinese or other ethnic group: 2%
Disability	411,595	33%
Number of ACEs	140,757	0.76 (1.13)
Loneliness	414,432	19%
Social isolation (on a scale of 0–2)	412,650	1.01 (0.34)
Number of hospital admissions	422,899	4.18 (13.66)
QALYs	343,796	0.81 (0.20)
Multimorbidity greater than or equal to 2	422,790	55%
Death in follow-up	422,898	7%
Coronary artery disease in follow up	400,050	7%
Heart failure in follow up	420,640	3%
Stroke in follow up	415,369	2%
Systolic blood pressure	421,649	137.79 (18.68) mmHg
Type 2 diabetes	422,899	5%
Self-harm	133,146	4%
Suicide attempt	132,956	2%
Depression	422,899	14%
Depression trait (PHQ-9 score; on a scale of 0–27)	131,040	2.75 (3.68)
Anxiety	422,899	9%
Anxiety trait (GAD-7 score; on a scale of 0–21)	131,638	2.15 (3.40)
Positive affect/happiness (on a scale of 1–6)	229,531	2.52 (0.74)
Meaning in life (on a scale of 1–5)	130,380	3.69 (0.83)

Note that the samples used in each analysis are a subset of this data depending on data availability.

*SD* standard deviation, *ACEs* adverse childhood experiences, *QALYs* Quality-adjusted life years, *CAD* Coronary artery disease, *PHQ-9* Patient Health Questionnaire 9-question version, *GAD-7* Generalised Anxiety Disorder 7-question version.

### Correlations

In UK Biobank, loneliness and social isolation were phenotypically correlated (Pearson’s correlation = 0.13; df = 405,767; 95% CI: 0.13–0.13; *p* < 0.001). Loneliness and social isolation were also genetically correlated, i.e., correlated genetic influences on both traits, based on GWAS data, (genetic correlation = 0.31; 95% CI: 0.25–0.38; *p* < 0.001). This demonstrates that the two phenotypes are capturing partially overlapping, but distinct traits.

### Triangulation approach

**Fig. 1:**
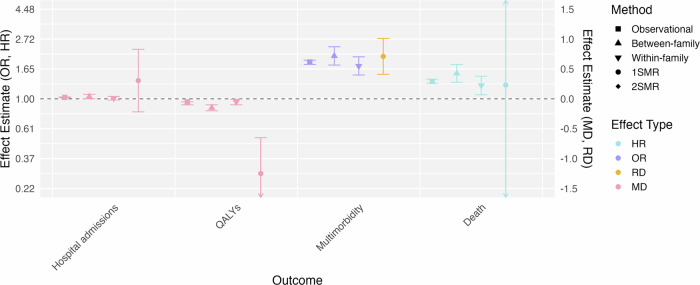
Main results from analyses examining relationships between loneliness and general health outcomes. 1SMR= One-sample Mendelian Randomisation, 2SMR= Two-sample Mendelian Randomisation, QALYs= Quality adjusted life years. There are no 2SMR results for these outcomes as these data were not available in genome-wide association studies. Data are presented as effects with 95% confidence intervals. For continuous outcomes the effect estimate presented is the standardised mean difference (MD). For binary outcomes the effect estimate is either presented as the hazards ratio (HR) where Cox proportional hazards models were used, the odds ratio (OR, plotted as the log(OR)) where logistic regression models were used or for 2SMR with a binary outcome, or the risk difference (RD) for 1SMR analyses where logistic regression models were not appropriate to use. For one-sample MR binary outcomes this is the approximate RD of the outcome per unit increase in genetically predicted loneliness and for continuous outcomes this is the MD in the outcome per unit increase in genetically predicted loneliness. Effects for hospital admissions reflect the standardised count of times they have been admitted to hospital, for QALYs this is the standardised percentage change in QALYs per year of follow-up, for multimorbidity this is whether someone had two or more health conditions. Observational analyses sample size = 332,507 for death; 118,947 for multimorbidity; 118,965 for hospital admissions; 93,307 for QALYs. Between- and within-family analyses sample size = 32,828 for death; 12,319 for multimorbidity; 12,322 for hospital admissions; 9837 for QALYs. 1SMR analyses sample size = 331,995 for death; 331,918 for multimorbidity; 331,996 for hospital admissions; 269,905 for QALYs.

**Fig. 2:**
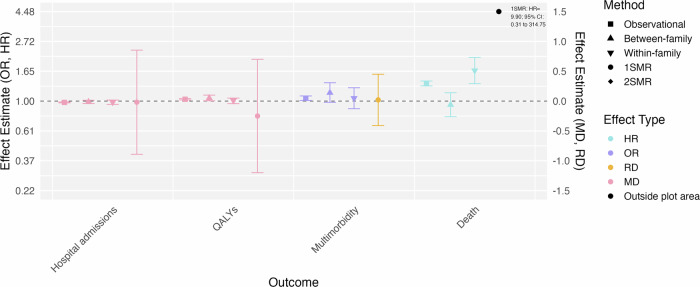
Main results from analyses examining relationships between social isolation and general health outcomes. 1SMR= One-sample Mendelian Randomisation, 2SMR= Two-sample Mendelian Randomisation, QALYs= Quality adjusted life years. There are no 2SMR results for these outcomes as these data were not available in genome-wide association studies. The point estimate for the 1SMR analysis for death was too large to plot alongside other points and therefore the results are shown in text on the plot. Data are presented as effects with 95% confidence intervals. For continuous outcomes the effect estimate presented is the standardised mean difference (MD). For binary outcomes the effect estimate is either presented as the hazards ratio (HR) where Cox proportional hazards models were used, the odds ratio (OR, plotted as the log(OR)) where logistic regression models were used or for 2SMR with a binary outcome, or the risk difference (RD) for 1SMR analyses where logistic regression models were not appropriate to use. For one-sample MR binary outcomes this is the approximate RD of the outcome per unit increase in genetically predicted social isolation and for continuous outcomes this is the MD in the outcome per unit increase in genetically predicted social isolation. Effects for hospital admissions reflect the standardised count of times they have been admitted to hospital, for QALYs this is the standardised percentage change in QALYs per year of follow-up, for multimorbidity this is whether someone had two or more health conditions. Observational analyses sample size = 335,593 for death; 119,929 for multimorbidity; 119,947 for hospital admissions; 94,086 for QALYs. Between- and within-family analyses sample size = 33,325 for death; 12,451 for multimorbidity; 12,454 for hospital admissions; 9950 for QALYs. 1SMR analyses sample size = 333,357 for death; 333,280 for multimorbidity; 333,358 for hospital admissions; 270,935 for QALYs.

**Fig. 3:**
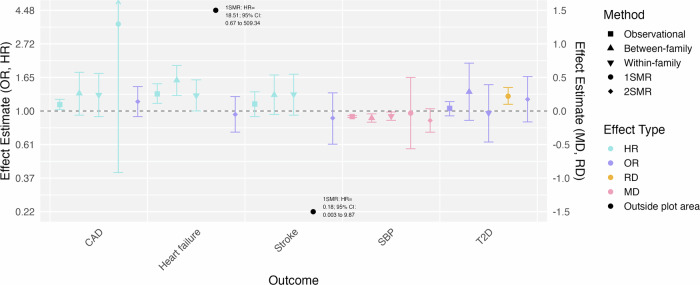
Main results from analyses examining relationships between loneliness and physical health outcomes. 1SMR= One-sample Mendelian Randomisation, 2SMR= Two-sample Mendelian Randomisation, CAD=Coronary artery disease, T2D=type 2 diabetes, SBP=systolic blood pressure. The point estimates for the 1SMR analysis for heart failure and stroke were too large to plot alongside other points and therefore the results are shown in text on the plot. Data are presented as effects with 95% confidence intervals. For continuous outcomes the effect estimate presented is the standardised mean difference (MD). For binary outcomes the effect estimate is either presented as the hazards ratio (HR) where Cox proportional hazards models were used, the odds ratio (OR, plotted as the log(OR)) where logistic regression models were used or for 2SMR with a binary outcome, or the risk difference (RD) for 1SMR analyses where logistic regression models were not appropriate to use. For one-sample MR binary outcomes this is the approximate RD of the outcome per unit increase in genetically predicted loneliness and for continuous outcomes this is the MD in the outcome per unit increase in genetically predicted loneliness. Effects for systolic blood pressure reflect standardised millimetres of mercury (mmHg). Observational analyses sample size = 115,557 for CAD; 118,693 for heart failure; 117,889 for stroke, 118,965 for T2D; 118,876 for SBP. Between- and within-family analyses sample size = 11,959 for CAD; 32,667 for heart failure; 32,310 for stroke, 12,322 for T2D; 12,317 for SBP. 1SMR analyses sample size = 314,205 for CAD; 330,211 for heart failure; 326,048 for stroke, 331,996 for T2D; 331,696 for SBP. 2SMR analyses genome-wide association study sample size 573,604 for loneliness; 1,347,212 for CAD; 977,323 for heart failure; 1,308,460 for stroke, 1,114,458 for T2D; 757,601 for SBP.

**Fig. 4:**
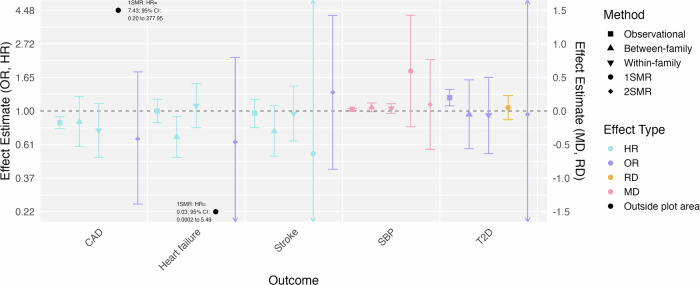
Main results from analyses examining relationships between social isolation and physical health outcomes. 1SMR= One-sample Mendelian Randomisation, 2SMR= Two-sample Mendelian Randomisation, CAD=Coronary artery disease, T2D=type 2 diabetes, SBP=systolic blood pressure. The point estimates for the 1SMR analysis for CAD and heart failure were too large to plot alongside other points and therefore the results are shown in text on the plot. Data are presented as effects with 95% confidence intervals. For continuous outcomes the effect estimate presented is the standardised mean difference (MD). For binary outcomes the effect estimate is either presented as the hazards ratio (HR) where Cox proportional hazards models were used, the odds ratio (OR, plotted as the log(OR)) where logistic regression models were used or for 2SMR with a binary outcome, or the risk difference (RD) for 1SMR analyses where logistic regression models were not appropriate to use. For one-sample MR binary outcomes this is the approximate RD of the outcome per unit increase in genetically predicted social isolation and for continuous outcomes this is the MD in the outcome per unit increase in genetically predicted loneliness. Effects for systolic blood pressure reflect standardised millimetres of mercury (mmHg). Observational analyses sample size = 116,512 for CAD; 119,673 for heart failure; 118,866 for stroke, 119,947 for T2D; 119,854 for SBP. Between- and within-family analyses sample size = 12,090 for CAD; 33,163 for heart failure; 32,796 for stroke, 12,454 for T2D; 12,449 for SBP. 1SMR analyses sample size = 315,599 for CAD; 331,588 for heart failure; 321,451 for stroke, 333,358 for T2D; 333,358 for SBP. 2SMR analyses genome-wide association study sample size = 457,086 for social isolation; 1,347,212 for CAD; 977,323 for heart failure; 1,308,460 for stroke, 1,114,458 for T2D; 757,601 for SBP.

**Fig. 5:**
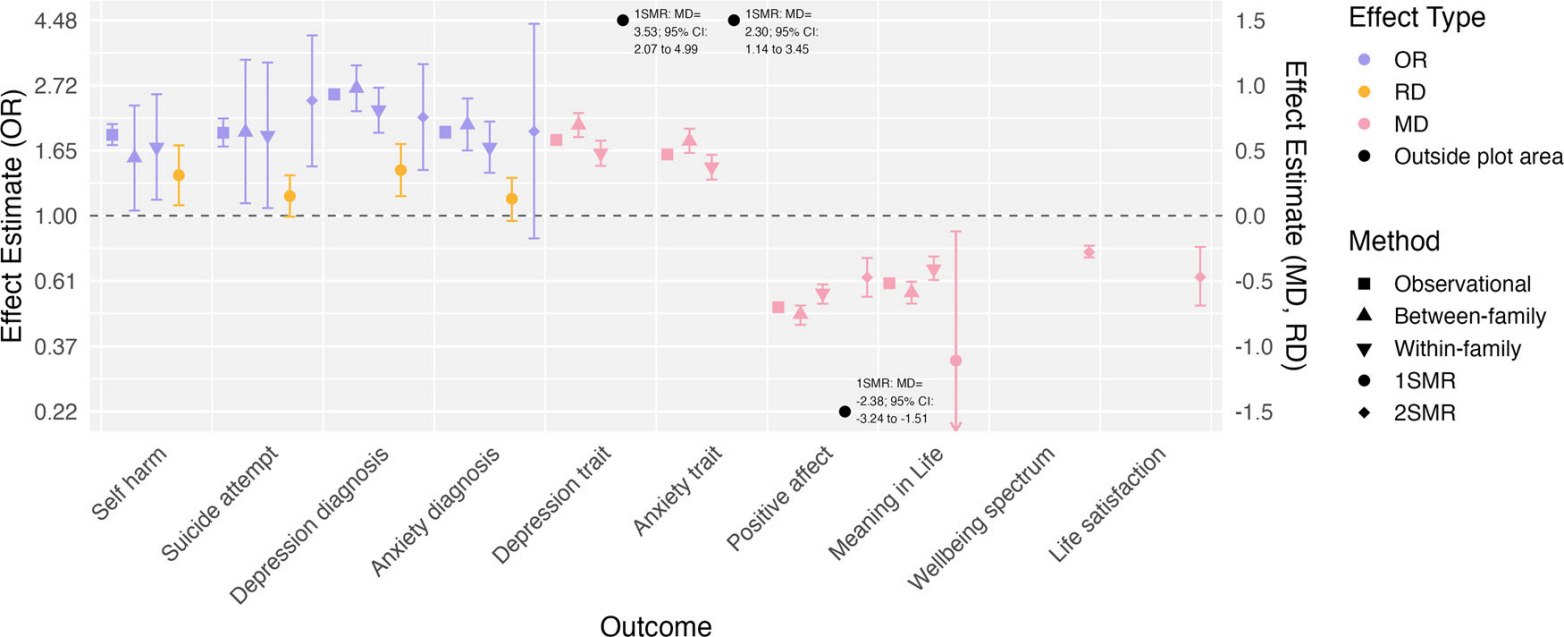
Main results from analyses examining relationships between loneliness and mental health and wellbeing outcomes. 1SMR= One-sample Mendelian Randomisation, 2SMR= Two-sample Mendelian Randomisation. There are no 2SMR results for self-harm, depression trait, anxiety trait or meaning in life as these data were not available in genome-wide association studies. There are only 2SMR results for wellbeing spectrum and life satisfaction because there were no equivalent measures in UK Biobank. The point estimates for the 1SMR analysis for depression trait, anxiety trait and positive affect were too large to plot alongside other points and therefore the results are shown in text on the plot. Data are presented as effects with 95% confidence intervals. For continuous outcomes the effect estimate presented is the standardised mean difference (MD). For binary outcomes the effect estimate is either presented as the odds ratio (OR, plotted as the log(OR)) where logistic regression models were used or for 2SMR with a binary outcome, or the risk difference (RD) for 1SMR analyses where logistic regression models were not appropriate to use. For one-sample MR binary outcomes this is the approximate RD of the outcome per unit increase in genetically predicted loneliness and for continuous outcomes this is the MD in the outcome per unit increase in genetically predicted loneliness. Effects for the depression trait reflect the standardised score (ranging from 0 to 27), effects for the anxiety trait reflect the standardised score (ranging from 0 to 21), effects for happiness/positive affect reflect the standardised rating (ranging from 1 to 6), effects for meaning in life reflect the standardised rating (ranging from 1 to 5). Observational analyses sample size = 79,000 for self-harm; 78,904 for suicide attempt; 118,965 for depression diagnosis, 118,965 for anxiety diagnosis; 78,213 for depression trait; 78,513 for anxiety trait, 92,761 for positive affect; 77,577 for meaning in life. Between- and within-family analyses sample size = 8154 for self-harm; 8146 for suicide attempt; 12,322 for depression diagnosis, 12,322 for anxiety diagnosis; 8077 for depression trait; 8100 for anxiety trait, 9464 for positive affect; 8004 for meaning in life. 1SMR analyses sample size = 108,966 for self-harm; 108,827 for suicide attempt; 331,996 for depression diagnosis, 331,996 for anxiety diagnosis; 107,357 for depression trait; 107,853 for anxiety trait, 178,666 for positive affect; 106,828 for meaning in life. 2SMR analyses genome-wide association study sample size = 573,604 for loneliness; 518,612 for suicide attempt; 142,646 for depression diagnosis, 17,526 for anxiety diagnosis; 2,083,151 for wellbeing spectrum; 2,083,151 for life satisfaction.

**Fig. 6:**
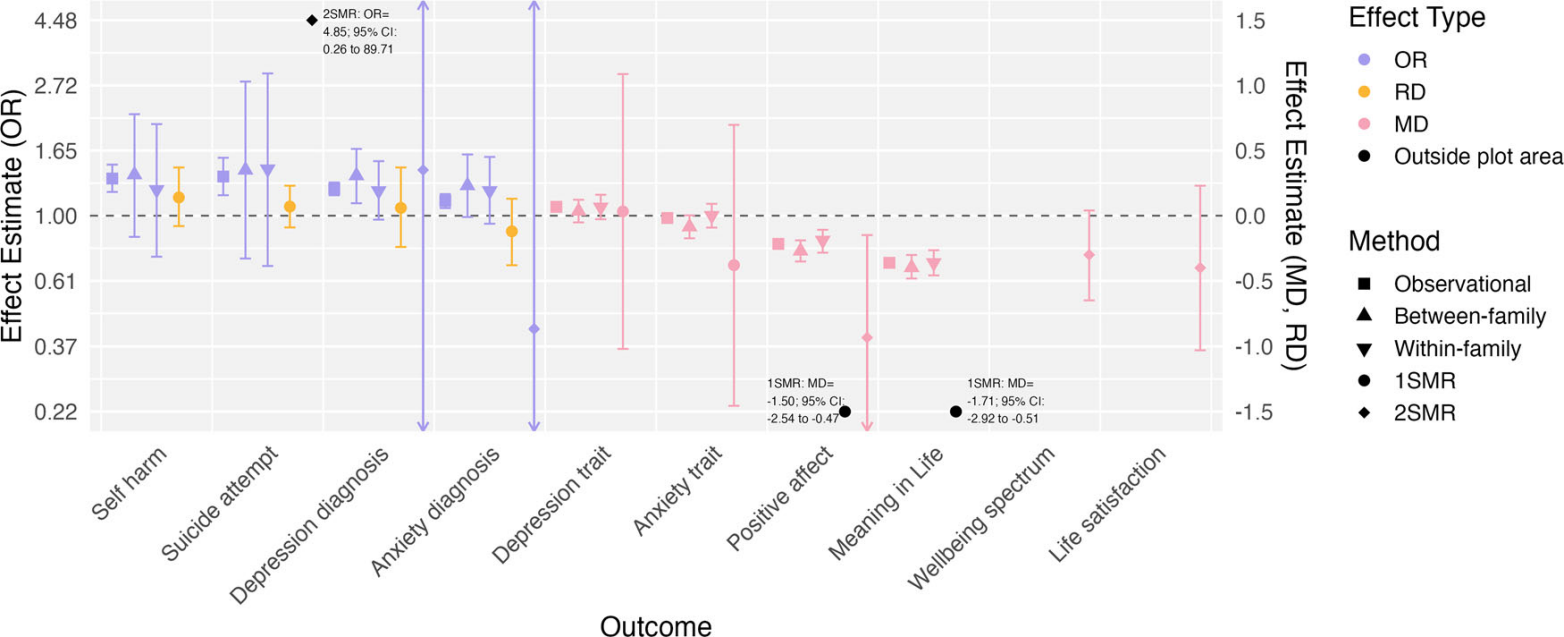
Main results from analyses examining relationships between social isolation and mental health and wellbeing outcomes. 1SMR= One-sample Mendelian Randomisation, 2SMR= Two-sample Mendelian Randomisation. There are no 2SMR results for self-harm, depression trait, anxiety trait or meaning in life as these data were not available in genome-wide association studies. There are only 2SMR results for wellbeing spectrum and life satisfaction because there were no equivalent measures in UK Biobank. The point estimates for the 2SMR analysis for suicide attempt and the 1SMR analysis for positive affect and meaning in life were too large to plot alongside other points and therefore the results are shown in text on the plot. Data are presented as effects with 95% confidence intervals. For continuous outcomes the effect estimate presented is the standardised mean difference (MD). For binary outcomes the effect estimate is either presented as the odds ratio (OR, plotted as the log(OR)) where logistic regression models were used or for 2SMR with a binary outcome, or the risk difference (RD) for 1SMR analyses where logistic regression models were not appropriate to use. For one-sample MR binary outcomes this is the approximate RD of the outcome per unit increase in genetically predicted social isolation and for continuous outcomes this is the MD in the outcome per unit increase in genetically predicted social isolation. Effects for the depression trait reflect the standardised score (ranging from 0 to 27), effects for the anxiety trait reflect the standardised score (ranging from 0 to 21), effects for happiness/positive affect reflect the standardised rating (ranging from 1 to 6), effects for meaning in life reflect the standardised rating (ranging from 1 to 5). Observational analyses sample size = 79,596 for self-harm; 79,500 for suicide attempt; 119,947 for depression diagnosis, 119,947 for anxiety diagnosis; 78,782 for depression trait; 79,081 for anxiety trait, 93,483 for positive affect; 78,148 for meaning in life. Between- and within-family analyses sample size = 8228 for self-harm; 8221 for suicide attempt; 12,454 for depression diagnosis, 12,454 for anxiety diagnosis; 8,153 for depression trait; 8176 for anxiety trait, 9561 for positive affect; 8075 for meaning in life. 1SMR analyses sample size = 109,432 for self-harm; 109,291 for suicide attempt; 333,358 for depression diagnosis, 333,358 for anxiety diagnosis; 107,790 for depression trait; 108,274 for anxiety trait, 179,367 for positive affect; 107,258 for meaning in life. 2SMR analyses genome-wide association study sample size = 457,086 for social isolation; 518,612 for suicide attempt; 142,646 for depression diagnosis, 17,526 for anxiety diagnosis; 2,083,151 for wellbeing spectrum; 2,083,151 for life satisfaction.

Results from our observational analyses, sibling control analyses, 1SMR and 2SMR analyses where loneliness and social isolation were the exposures are summarised in Figs. 1–6 and detailed in Supplementary Tables S5 (observational), S8 (sibling control), S9 (1SMR) and S13 (2SMR). The effect sizes from these analyses are not always directly comparable because of the different approaches and measures used. We were mainly interested in how consistent the direction of effect was across the different analyses, with different assumptions, and how similar effect sizes were where approaches were similar (e.g., observational and sibling control). Details of each analysis can be found in the Methods. We have presented our main results for each of these outcome groups below, followed by sensitivity analyses. To summarise, we found evidence of effects of loneliness and social isolation on poorer mental health and wellbeing and of loneliness on poorer general health, with stronger effects for loneliness compared to social isolation in our results. We did not find evidence of effects of loneliness and social isolation on physical health outcomes, although we were unable to definitely rule out these effects.

### Triangulation approach for general health outcomes

Overall, we found evidence across all approaches for effects of being lonely on decreased QALYs (*p *$\le$ 0.001 to 0.03) and increased risk of multimorbidity (*p *$\le$ 0.001) (Fig. 1; Supplementary Tables S5, S8 and S9). For death and hospital admissions, there were associations between being lonely and an increased hazard for death across all approaches, except MR (*p *$\le$ 0.001 to 0.006) as well as increased rates of hospital admissions in the observational and sibling control analyses for the between-family effect only (*p *$\le$ 0.001 to 0.04); the corresponding MR estimates were not inconsistent but were very imprecise (Fig. 1; Supplementary Tables S5, S8, and S9). We also found associations between increased social isolation and an increased hazard for death in the observational and the sibling control analyses for the within-family effect only (*p *$\le$ 0.001); again, the corresponding MR estimate was not inconsistent but was imprecise (Fig. 2; Supplementary Tables S5, S8 and S9). We did not find consistent evidence of effects where social isolation was the exposure for any of the other general health outcomes, as effects were only found in observational analyses (*p *$\le$ 0.001 to 0.02) (Fig. 2; Supplementary Tables S5, S8 and S9). We note that the Cox proportional hazards models used for the 1SMR, where death was the outcome were likely too imprecise to reliably assess evidence of an effect. MR analyses are less powerful than other methods applied to the same sample size. This is exacerbated by the reduced statistical power of a Cox model when the event is rare. Where we do not see evidence of effects, confidence intervals were wide so should not be interpreted as evidence of absence of an effect.

### Triangulation approach for physical health outcomes

There was some evidence of effects of being lonely on decreased systolic blood pressure (*p *$\le$ 0.001 to 0.91) and an increased hazard for heart failure (*p *$\le$ 0.001 to 0.72) observed across all analyses, other than the MR analyses (Supplementary Tables S5, S8, S9 and S13). There was, however, a lack of evidence generally for being lonely on other physical health outcomes (*p* > 0.111 for coronary artery disease, *p* > 0.109 for stroke, *p* > 0.183 for type 2 diabetes), other than observationally for an increased hazard of coronary artery disease (*p* = 0.018; Supplementary Table S5) and in 1SMR analyses for increased type 2 diabetes risk (*p *$\le$ 0.001; Supplementary Table S9). There was evidence for observational effects on an increased hazard of stroke and increased type 2 diabetes risk (*p* $\le$ 0.001), but after adjusting for ACEs and/or disability these effects attenuated (*p* = 0.286 for stroke and *p* = 0.496 for type 2 diabetes) (Supplementary Table S5). We did not find evidence of effects where social isolation was the exposure for any physical health outcomes (*p* > 0.157 for coronary artery disease, *p* > 0.186 for heart failure, *p* > 0.114 for stroke, *p* > 0.576 for type 2 diabetes, *p* > 0.103 for systolic blood pressure, Supplementary Tables S5, S8, S9 and S13), other than observationally for increased systolic blood pressure (*p* = 0.002), a decreased hazard of coronary artery disease (*p *$\le$ 0.001, although the effect in the unadjusted model was for an increased hazard of coronary artery disease) and increased type 2 diabetes risk (*p* = 0.002) (Supplementary Table S5) and in the between-family model for an increased hazard of heart failure (*p* = 0.013; Supplementary Table S8). With heart failure and stroke, effects were observed in unadjusted models (*p* < 0.001) but not in the adjusted models (*p* = 0.980 for heart failure and *p* = 0.745 for stroke; Supplementary Table S5). We note that the Cox proportional hazards models used for the 1SMR, where heart failure, coronary artery disease and stroke were outcomes, were also likely too imprecise to reliably assess evidence of an effect. MR analyses are less powerful than other methods applied to the same sample size, and this reduced statistical power in our 1SMR analyses was not mitigated by the large sample sizes in the GWAS used for the 2SMR. This is exacerbated by the reduced statistical power of a Cox model when the event is rare. Where we do not see evidence of effects, confidence intervals were wide so should not be interpreted as evidence of absence of an effect.

### Triangulation approach for mental health and wellbeing outcomes

Overall, we found evidence across all analyses for effects of being lonely on increased risk of self-harm (*p *$\le$ 0.001 to 0.008), suicide attempt (*p *$\le$ 0.001 to 0.062), depression diagnosis (*p *$\le$ 0.001), increased levels of the depression trait (*p *$\le$ 0.001) and anxiety trait (*p *$\le$ 0.001) and decreased levels of happiness/positive affect (*p *$\le$ 0.001), meaning in life (*p *$\le$ 0.001 to 0.028), wellbeing (*p *$\le$ 0.001) and life satisfaction (*p *$\le$ 0.001) (Supplementary Tables S5, S8, S9 and S13). There was some evidence of effects of being lonely on anxiety diagnosis (*p *$\le$ 0.001) in all analyses other than 1SMR and 2SMR (*p* = 0.131 and *p* = 0.123, respectively), although the 2SMR analysis had lower statistical power due to the smaller sample size compared to other outcome GWAS (Supplementary Tables S5, S8, S9 and S13). We also found consistent evidence across analyses of effects of increased levels of social isolation on lower levels of happiness/positive affect (*p *$\le$ 0.001 to 0.020) and meaning in life (*p *$\le$ 0.001 to 0.008). However, this was not the case for the other mental health and life satisfaction outcomes (*p* > 0.193 for self-harm, *p* > 0.289 for suicide attempt, *p* > 0.096 for depression diagnosis, *p* > 0.167 for depression trait, *p* > 0.062 for anxiety diagnosis, *p* > 0.057 for anxiety trait, *p* = 0.082 for wellbeing and *p* = 0.211 for life satisfaction; Supplementary Tables S5, S8, S9 and S13), other than observationally for increased risk of self-harm (*p *$\le$ 0.001), suicide (*p *$\le$ 0.001), depression diagnosis (*p *$\le$ 0.001), increased depression trait (*p*$\le$0.001) and increased risk of anxiety diagnosis (*p *$\le$ 0.001) (Supplementary Table S5) and in the between-family model for depression diagnosis (*p* = 0.004; Supplementary Table S8). Where we do not see evidence of effects, confidence intervals were wide so should not be interpreted as evidence of absence of an effect.

### Sensitivity analyses for all outcomes

For the observational analyses using alternative models due to data being positively skewed (i.e., Gamma and negative binomial models) for hospital admissions, depression trait and anxiety trait, results were similar (Supplementary Table S6). Similarly, when using a slightly different measure for social isolation (excluding children in household) results were similar (Supplementary Table S7). We also conducted 1SMR analyses using unweighted genetic scores for loneliness from the published GWAS (we used the split sample approach in main analyses due to sample overlap and this additional analysis to compare results). Effects from this analysis were similar to or attenuated compared to the split sample main approach for loneliness (Supplementary Table S10). We also used a 2SMR framework to conduct sensitivity analyses for the 1SMR analyses (using weighted median and weighted mode approaches). In these sensitivity analyses, effects were mostly attenuated and there was generally a lack of evidence of an effect with the weighted mode approach, but evidence of an effect (where effects were observed in the main analyses) with the weighted median approach (Supplementary Table S11). This may suggest that some of the genetic variants used were weak, which could be attributed to horizontal pleiotropy (where the genetic variant influences the outcome through a pathway not via the exposure).

For our 2SMR analyses, effects tended to be consistent across 2SMR sensitivity analyses, with the exception of the MR-Egger^52^ and simulation extrapolation (SIMEX)-adjusted MR-Egger. This may indicate the presence of horizontal pleiotropy, although given the wide confidence intervals and the low I-squared values (Supplementary Table S12) it is possible that these tests were too imprecise to assess this reliably. Other tests (Supplementary Table S13) did not show strong evidence for the presence of horizontal pleiotropy. Our MR-Causal Analysis Using Summary Effect estimates (CAUSE)^53^ results indicated that there was evidence supporting a causal model over a sharing model for loneliness with depression, wellbeing spectrum and positive affect, and social isolation with wellbeing spectrum and positive affect. We also used Steiger filtering to estimate the percentage of genetic variants in the instrument that explained more variance in the exposure than the outcome, which can help to infer the most likely direction of effect^54^. These results indicated that, for the most part, a high percentage (>80%) of genetic variants explained more variance in the exposure than the outcomes, suggesting the exposures are more likely to cause the respective outcomes than vice versa. This was lower for loneliness and life satisfaction (58%), suggesting the direction of effect is less clear in this case.

### Analyses with loneliness and social isolation as outcomes

We were interested in assessing whether relationships might be bidirectional using 2SMR with loneliness and social isolation as the outcomes, (see Supplementary Table S14). These analyses were restricted to those where summary level GWAS data was available, similar to our other two-sample analyses (i.e., not including hospital admissions, QALYs, multimorbidity, death, self-harm, depression trait, anxiety trait and meaning in life). We were interested in whether there was evidence to suggest there may also be effects operating in the opposite direction, but we were not focused on interpreting the magnitudes of these effects. Similar to analyses with loneliness and social isolation as exposures, we found evidence across the main 2SMR and sensitivity analyses for effects of depression diagnosis on increased loneliness (*p* < 0.001; Supplementary Table S14) and wellbeing spectrum, positive affect and life satisfaction on decreased loneliness (*p* < 0.001; Supplementary Table S14). Where life satisfaction was the exposure the F-statistic was 3.49 (Supplementary Table S12), which likely indicates the presence of weak instrument bias^18^. Given this, the findings for life satisfaction should be interpreted with caution.

For social isolation there was some suggestive evidence for depression diagnosis influencing increased social isolation, but this was inconsistent across sensitivity analyses (Supplementary Table S14). The most consistent results across sensitivity analyses were observed for decreased wellbeing spectrum, positive affect and life satisfaction on increased social isolation (Supplementary Table S14). Again, given the F-statistic of 3.49 for life satisfaction (Supplementary Table S12), the results likely indicate the presence of weak instrument bias, and caution should be taken when interpreting them.

### Analyses examining independent effects of loneliness and social isolation

We conducted multivariable MR (MVMR) analyses with both loneliness and social isolation as exposures to assess whether their influences on outcomes were independent (Supplementary Table S15). MVMR allows for the inclusion of multiple exposures to estimate the effect of each exposure conditioned on the other. Results supported 2SMR findings for increased loneliness on increased risk of suicide attempt, depression and decreased levels of wellbeing spectrum, positive affect and life satisfaction. These results were consistent across sensitivity analyses, with the exception of MR-Egger, where confidence intervals were wide (Supplementary Table S15). This suggests that social isolation did not impact the influence of loneliness on these outcomes.

For social isolation the 2SMR effect observed on decreased positive affect seemed to attenuate with no evidence of an effect remaining when loneliness was also accounted for, suggesting loneliness may influence this effect (e.g., as a mediator).

## Discussion

Using a triangulation approach, our results provide consistent evidence across analyses that loneliness may causally negatively impact mental health and wellbeing outcomes, and that social isolation may causally negatively impact wellbeing outcomes. This is in line with previous studies demonstrating observational associations between loneliness, social isolation and poorer mental health^1,7,8^. Our findings suggest that effects on mental health and wellbeing may be stronger for loneliness and that loneliness could also be influencing the effect of social isolation on positive affect. We also found consistent evidence across analyses that being lonely may causally negatively impact some aspects of general health (i.e., increasing the risk of multimorbidity and decreasing QALYs), in line with previous studies^1^. However, in contrast to previous observational studies^2,14^ we did not find consistent evidence across analyses of effects of social isolation on general health, or of either loneliness or social isolation on specific physical health outcomes.

We considered our results across the three different sets of analyses, as each analysis is prone to different limitations and biases. For example, the exposures in our MR analyses are not biological exposures and may be prone to horizontal pleiotropy (i.e., the instrument influencing the outcomes through pathways not via the exposure). In addition, some of our analyses (e.g., the one-sample MR analysis for loneliness and death) resulted in very imprecise estimates. A key strength of this study is the integration of multiple analyses through a triangulation approach, which allows for a comprehensive examination of each exposure-outcome relationship and enables the evaluation of conclusions based on the results from these diverse analyses.

We found comparable effect sizes with previously published observational effects (e.g., for depression and death)^7,55^. Our results therefore suggest potentially large negative effects of loneliness on mental health. We also found evidence of effects in the other direction (i.e., poorer mental health and wellbeing causing loneliness or greater social isolation). This suggests that the relationships between loneliness and social isolation and mental health and wellbeing may exacerbate each other.

The lack of evidence for effects of loneliness and social isolation on physical health outcomes and some general health outcomes does not mean that causal relationships do not exist. Our findings may reflect an absence of evidence as opposed to evidence of absence, particularly given previous findings in longitudinal studies suggesting effects of loneliness and social isolation on physical health outcomes, including cardiovascular disease and type 2 diabetes^3,14^. Our MR analyses, and some of our sibling control analyses, were not precise enough (i.e., confidence intervals were wide) to provide evidence for, or against, a potential causal interpretation in many cases. However, often, we found evidence of effects observationally, where we adjusted for important potential confounders, but not in the sibling control or MR analyses. This may suggest that there are important familial confounders or shared genetic predispositions that influence these associations. Furthermore, the fact we find more consistent evidence of effects for loneliness on general health, but not specific physical health outcomes (which likely influence general health) is interesting and could be due to several reasons. First, it may suggest that the effects of individual physical health outcomes were too small to detect in our study, but the combined effects of these influence general health. Second, it may be that because the general health measures also capture mental health, that mental health outcomes are driving these effects. Third, we examined only a select number of physical health outcomes, and it may be that there are effects on other physical health outcomes not examined here (i.e., non-cardiovascular outcomes). Previous studies have found effects on other physical health outcomes that we did not examine, such as dementia, Alzheimer’s disease, asthma and osteoarthritis, amongst others^4,56,57^, and these merit examination in future triangulation studies. Outcomes examined in this study were chosen for being particularly burdensome health outcomes with previously observed associations with loneliness and/or social isolation^1,7,12,38,40,44–46,48,51^.

Our results are in line with a recent study that included observational analyses in UK Biobank and MR analyses^27^. That study differed from the current study in the specific exposures and GWAS used. They also included fewer analyses (i.e., no sibling control analyses or 1SMR analyses) and fewer sensitivity analyses, which are important given that loneliness and social isolation are complex phenotypes and MR assumptions e.g., around pleiotropy, are more likely to be violated. However, they found similar results to those in this study. Our findings draw on a robust pre-registered triangulation approach strengthening the conclusions drawn in both our study and this recent study.

A final aspect to discuss is that the social isolation measure we used is different to some previous observational studies in this area, which may partially explain the differential findings. Our measure of social isolation did not include group activities and was focused on household size and frequency of seeing friends/family. Unlike some previous studies^58,59^, we opted not to include measures related to group activities or going to venues such as a gym or pub, because those may be capturing other behaviours that influence health outcomes through routes other than social interaction.

### Strengths and limitations

The main strength of our study is the use of a triangulation approach. The different methods we used have their own strengths and biases, but by interpreting the results together we have greater confidence in our findings. Another strength is the range of outcomes we explored which incorporate both self-reported and linked National Health Service (NHS) electronic care record data, covering different aspects of general, physical and mental health and wellbeing in an in-depth manner. We also included an extensive number of sensitivity analyses across our approaches which address different biases and assumptions, resulting in more robust conclusions. Finally, we used data from large and recent GWAS and UK Biobank, a large cohort study with rich phenotypic data.

There are several limitations to the current study that should be considered when interpreting our findings. First, there were several analyses, particularly where social isolation was the exposure, where our MR estimates had wide confidence intervals meaning that statistical power may have been lacking to detect effects. Second, our measure of loneliness in UK Biobank was a binary response to a single question asking whether the respondent was lonely, whereas other studies have used composite measures of loneliness. Loneliness is likely to be captured better using such validated composite measures that include different aspects of loneliness. The single item measure we have used could result in weaker associations in this study. Similarly, our social isolation measure did not capture social interactions outside of the household, friends and family, potentially missing key sources of social interaction. Furthermore, our measures of both loneliness and social isolation were assessed at a single time point during middle-late adulthood. Both loneliness and social isolation are likely to change across the life course, and important time periods for loneliness and social isolation e.g., childhood, adolescence and young adulthood are not captured in this study. It may be that effects on the health outcomes studied here vary across different life stages and therefore conclusions about the impact of loneliness and social isolation in earlier life are limited. However, even a single measurement of loneliness is likely to reflect chronic loneliness in some individuals. In addition, by including only a single timepoint we were able to able to examine outcomes that temporally occurred after the exposure was measured. We did include MR analyses which should provide estimates of effects over the lifetime, but these results would be strengthened by the inclusion of analyses in age groups not covered in UK Biobank. Third, whilst we accounted for several important potential confounders in our observational and sibling control analyses, there may be factors that we could not account for. Fourth, results from our sibling analyses may not be generalisable because it required there to be at least two siblings from the same family participating in UK Biobank. In terms of representation, UK Biobank participants are generally healthier and less socioeconomically deprived than the general population^60^ and our MR analyses were restricted to individuals of European ancestry. Therefore, our results may not be generalisable outside of those populations. In addition, this may mean that there are fewer cases in terms of the health outcomes examined, continuous measures may be skewed towards ‘healthier’ ranges, and rates of loneliness and social isolation may be lower than in the general population. Individuals with poorer health or who are more lonely or socially isolated may have been less likely to participate in UK Biobank, introducing selection bias in our analyses which could bias associations. Therefore, caution should be taken when interpreting results from analyses based solely on UK Biobank data. Fifth, in our two-sample MR analyses where we included binary exposures, there are limitations to interpreting these effects where these exposures may reflect dichotomisation of an underlying continuous trait e.g., a diagnosis of depression reflects a clinical cut-off of an underlying continuous distribution of depressive symptoms^61^. The main limitation of including binary exposures is that the effect estimate may not be particularly interpretable, Therefore, results from MR analyses with binary exposures in this study should be considered in terms of direction of effect as opposed to overly focusing on the magnitude of the effect. Sixth, loneliness and social isolation are unlikely to be directly caused by the genetic variants used as instruments in this study (i.e., they are biologically distal and the pathways from genetic variants to these exposures are likely to be complex). MR analyses are likely to be more reliable when exposures are more directly related to the genetic variants included as instruments, for example, when using biological exposures such as blood-based measures^16^. Additionally, biologically distal exposures may increase the chances of there being horizontal pleiotropy. Although we conducted a range of sensitivity analyses to address this, it may be that pleiotropic pathways do still exist. This should be considered when interpreting the MR results and the MR results should also be interpreted alongside the other analyses within the triangulation framework.

### Future directions

Given the findings in this study there are some useful directions that future research could take. It is important to better understand the pathways from loneliness/social isolation to mental health and wellbeing outcomes to target effective interventions to improve these outcomes. Examining the impact of loneliness and social isolation across the life course would provide additional insight, but this was not possible in the current study given the data available. Longitudinal studies (including chronic loneliness and social isolation) would be useful to provide a more comprehensive understanding of the impact of these exposures on health outcomes. In addition, examining relationships with other physical outcomes would also be helpful. Finally, further analyses that would complement this triangulation approach would be useful. For example, within-family MR would be helpful to better account for confounding due to factors such as population stratification and dynastic effects. We did not include this approach here as using the sibling data in UK Biobank would result in underpowered analyses^62^, due to the small sample sizes we would have when combined with a split sample approach (similar to our one-sample approach, a split sample approach would be required given the sample overlap in the GWAS and UK Biobank). Further analyses using larger samples with data from mother, father, and child trios or sibling pairs may provide greater statistical power; however, the availability of such data to support this type of analysis remains limited.

Our study used a combination of approaches, including some that were novel or had limited prior applications in this field (i.e., sibling control and MR analyses), to investigate whether loneliness and social isolation may influence a variety of general, physical and mental health and wellbeing outcomes. Overall, our triangulation study provided robust evidence suggesting effects of loneliness and social isolation on poorer mental health and wellbeing and of loneliness on poorer general health. Taken together, these findings suggest that loneliness, and potentially social isolation, are still important public health concerns, especially with respect to mental health and general health. Interventions targeting loneliness and social isolation may prove to be effective strategies for improving a range of mental health, wellbeing and general health outcomes.

## Methods

### Ethics

UK Biobank received ethics approval from the UK National Health Service Research Ethics Committee (REC reference for UK Biobank is 11/NW/0382) and all participants gave informed consent to the use of their anonymised data and samples for any health-related research and for UK Biobank to access their health-related records. Participants in UK Biobank were not reimbursed although travel expenses could be claimed.

### Pre-registration

The analysis plan is pre-registered on the Open Science Framework (10.17605/OSF.IO/GWPNY; pre-registered 18/12/2023). The analysis plan is located under the ‘Files’ section. Deviations from this analysis plan are outlined in Supplementary Methods Section 1.

### Data sources and measures

#### UK Biobank

For conventional multivariable observational, sibling control, and 1SMR analyses we used phenotypic and genetic data from the UK Biobank, a large population-based prospective cohort of around 500,000 participants aged between 38 and 73 years and living in the UK, recruited between 2006 and 2010^63^. We excluded participants who withdrew their consent using the latest withdrawal lists for this project. Access to UK Biobank was provided under an approved project (project number: 81499).

The phenotypic data we used are described in detail in Supplementary Table S2. To summarise, the UK Biobank loneliness measure was a binary variable, where participants were asked whether they often felt lonely. The social isolation measure was a continuous variable we created by combining measures of frequency of friend/family visits and the number of people living in their household. We assigned values between 0 and 1 for each measure and then summed these to create a final measure with values between 0 and 2 where 2 indicated greater social isolation. We included the following general health outcomes; hospital admissions, quality-adjusted life years (QALYs), multimorbidity and mortality, the following physical health outcomes; coronary artery disease, heart failure, stroke, systolic blood pressure, and type 2 diabetes and the following mental health/wellbeing outcomes; self-harm, suicide attempts, depression (binary/diagnosis and a continuous trait from the Patient Health Questionnaire 9-question version; PHQ-9, scale from 0 to 27), anxiety (binary/diagnosis and a continuous trait from the Generalised Anxiety Disorder 7-question version; GAD-7, scale from 0 to 21), positive affect (scale from 1 to 6) and meaning in life (scale from 1 to 5).

In UK Biobank there were 488,377 participants with genetic data available. Details on the quality control and other processing steps prior to using these data are described in Supplementary Methods Section 2. The number of UK Biobank participants for our main analyses ranged from 77,577 to 414,432 for observational analyses, 8004 to 40,440 for sibling control analyses and 106,828 to 333,358 for 1SMR. The difference in sample sizes was due to the data available for each measure.

#### Genome-wide association studies (GWAS)

We used publicly available GWAS summary statistics (*N* = 17,526 to 2,083,151) for our 2SMR analyses and for our exposures of loneliness and social isolation in 1SMR analyses (see Supplementary Table S1). However, we conducted our own GWAS of social isolation in UK Biobank (see Supplementary Methods Section 3, Supplementary Fig. S1 and Supplementary Table S3). There are GWAS of measures related to social isolation that already exist^23^. However, those typically focus on binary measures related to frequency of social contacts or on specific leisure activities. Therefore, to ensure as much information as possible was captured in the exposure, we conducted our own GWAS of the social isolation measure described above.

### Statistical analyses

In our triangulation approach we first conducted observational analyses to establish whether there was an association between loneliness or social isolation and a given health outcome. We then used sibling control and Mendelian Randomisation (MR) analyses to provide evidence of whether there are effects of these exposures on the health outcomes. Triangulation is particularly useful when different methods have different biases and assumptions. If results are consistent across the different methods, there is greater confidence in the results^64^. Our triangulation approach was qualitative (i.e., assessing whether there was consistent evidence of effects). Therefore, we have not focused on significance thresholds, where results are dichotomised based on arbitrary p-value thresholds^65^, and have not adjusted for multiple testing. We have instead considered evidence across the main analyses and sensitivity analyses and have also considered the precision of estimates when interpreting results.

No statistical method was used to predetermine sample size as we used data that was available in UK Biobank. However, in the pre-registration we included power calculations for our MR and sibling control analyses. These did not guide how we conducted our analyses (i.e., we conducted them all regardless of power), but they did guide interpretation of our findings. The power calculations indicated that some analyses would likely be underpowered. For example, where anxiety and suicide were the outcomes for loneliness and social isolation exposures, and where social isolation was the exposure, many of these analyses may only be powered to detect larger effect sizes, and therefore results should be interpreted taking this into consideration.

All analyses were conducted in R version 4.1.0^66^. All statistical tests were two-tailed tests.

### Correlation between loneliness and social isolation

We calculated the phenotypic Pearson correlations between the loneliness and social isolation measures in UK Biobank. We also calculated the genetic correlation between these using GWAS summary statistics for loneliness and social isolation using linkage disequilibrium (LD) score regression^67^.

### Observational and sibling control analyses in UK Biobank

We conducted linear regressions (for continuous outcomes), logistic regressions (for binary outcomes) or Cox proportional hazard models (time-to-event outcomes) (i.e., all-cause mortality and incident coronary artery disease, heart failure and stroke). Supplementary Methods Section 4 describes how we applied the Cox proportional hazards models in this study.

In all observational and sibling control analyses we adjusted for potential confounders with two levels of adjustment. We do not adjust for these in our MR analyses as this would be inappropriate given that these potential confounders occur post-conception (and therefore cannot influence genetic variants) and the assumption in MR related to confounding refers to there being no confounding of the genetic variants and outcomes. In our first adjusted models we included potential demographic confounders (age when attended assessment centre, sex, assessment centre, socioeconomic position as measured by the Townsend deprivation index, education years based on qualifications^68^, household income before tax and ethnicity). In the second adjusted model we included all previous potential confounders as well as long-standing illness/disability/infirmity and a score for adverse childhood experiences (ACEs)^69^. We additionally conducted analyses using our first level of adjustment as well as 1) long-standing illness/disability/infirmity or 2) ACEs to examine the impact of these factors further. We note that disability, in particular, could be considered an outcome as well as a factor influencing loneliness, which could introduce potential collider bias and therefore results should be considered across all levels of adjustment. Details of potential confounders are presented in Supplementary Table S4. We also conducted a number of additional sensitivity analyses to assess whether findings were robust under different models or with a different social isolation exposure accounting for number of children in the household (see Supplementary Methods Section 5).

In the sibling control analyses we only included (biological) siblings thus controlling for shared environmental confounding (e.g., childhood household SES) and partly shared genetic predispositions^70^. The approach we used to identify siblings in UK Biobank to include in analyses is described in Supplementary Methods Section 6. In the sibling-control analyses, we calculated the sibship loneliness/social isolation average (the between-family effect) and each sibling’s deviation from the sibship average (the within-family effect, unbiased by shared familial confounders). Each of the outcomes was assessed using a model that included the between- and within-family estimates, and adjusting for the same factors as included in the observational models with the full sample.

#### Mendelian Randomisation (MR) analyses

MR is a method that uses genetic variants as instrumental variables for an exposure of interest. MR is less prone to confounding by socioeconomic, environmental and behavioural characteristics, and by reverse causation, than conventional observational studies^17,18^.

There are three core assumptions underlying MR:

1. The genetic instruments are robustly associated with the exposure of interest;
2. There is no confounding of the genetic instrument-outcome relationship;
3. The genetic instruments are only associated with the outcome via the exposure, and there are no direct effects between the genetic IV and the outcome

2SMR analyses used data obtained from GWAS of both the exposure and outcome of interest, whereas 1SMR analyses used GWAS data for the exposure of interest but individual level outcome data. Where data were available, we conducted both 2SMR analyses and 1SMR analyses. However, for some outcomes (hospital admissions, QALYs, multimorbidity, mortality and meaning in life) there were no GWAS available, or the data were only available in UK Biobank (resulting in full sample overlap with the exposure) and in this case, we only conducted 1SMR.

#### One-sample MR

For 1SMR analyses we used the Applied Econometrics with R (AER) package in R^71^ to conduct two-stage least squares regressions, except for where we used Cox proportional hazards models. For our continuous and binary outcomes we created genetic scores for our exposures of interest in UK Biobank and then used these in the two-stage least squares regression approach to estimate our effects. For the Cox proportional hazards models, we manually conducted the 1SMR using the ratio method^72^. 1SMR analyses were not bidirectional.

In our 1SMR analyses we adjusted for age, sex, the first 20 principal components (PCs) from PC analysis of genotype data, assessment centre and genotyping chip (Supplementary Table S4). Due to the exposure GWAS completely (social isolation) or partially (loneliness) being conducted in UK Biobank, and to thus avoid sample overlap which can bias estimates due to overfitting^73^, we used a split sample approach^74^. For this approach we split the sample into two random halves and in each half, we conducted a GWAS on social isolation and loneliness in UK Biobank (see Supplementary Methods Section 3). We used this GWAS data to create genetic scores in the other half of the sample, by using genetic variants and their weights with a p-value threshold of 1 × 10^−05^. We then used these to conduct our 1SMR analyses in each half of the sample. The results from the analyses in each half were then combined in a meta-analysis. Further details of this approach and sensitivity analyses conducted can be found in Supplementary Methods Section 7.

#### Two-sample MR

We conducted bidirectional 2SMR analyses (i.e., with loneliness/social isolation as exposures and outcomes) using the ‘TwoSampleMR’ R package^35^. We used summary data (including weights) of genetic variant-exposure genome-wide associations from published GWAS as described in Supplementary Table S1 as genetic instruments for the exposures in our analyses. These were selected based on a p-value threshold of 5×10^−08^ for all analyses, except where suicide attempt (1 × 10^−06^) and anxiety (1 × 10^−05^) were the exposures. The steps taken to prepare these data for analyses are described in Supplementary Methods Section 8. For the main analyses we used the inverse-variance weighted (IVW) method^75^. The sensitivity analyses assess various assumptions and potential biases in the MR approach. Inconsistent results may suggest bias from MR assumptions being violated. The sensitivity analyses included were MR-Egger^52^, weighted median^76^, MR using robust adjusted profile score (MR-RAPS)^77^, MR Pleiotropy RESidual Sum and Outlier (MR-PRESSO)^78^, MR lap^79^ and MR-Causal Analysis Using Summary Effect estimates (CAUSE)^53^. We also conducted Steiger filtering^54^ to further infer the most likely direction of effect. Supplementary Methods Section 8 further describes these sensitivity analyses.

#### Multivariable MR analyses

We additionally conducted multivariable MR (MVMR)^80^ analyses, using the ‘MVMR’ package in R, to estimate independent effects of loneliness and social isolation on health outcomes. MVMR estimates the direct effect of multiple exposures on an outcome in a single estimation and is not biased by the inclusion of genetic variants associated with many/all of the exposures in the model. Genetic instruments for both exposures were included to allow the effect of loneliness and social isolation on the outcome to be estimated, whilst conditioning on the other. Sensitivity analyses to detect and correct for potential horizontal pleiotropy were also included (see Supplementary Methods Section 9 for further details).

### Reporting summary

Further information on research design is available in the Nature Portfolio Reporting Summary linked to this article.

## Supplementary information

Supplementary Information

Reporting Summary

Transparent Peer Review file

## Data Availability

The UK Biobank data are available under restricted access for approved researchers, access can be obtained through a procedure described at http://www.ukbiobank.ac.uk/using-the-resource/. Access details for GWAS data used in this study are outlined below: Social isolation: GWAS summary statistics generated as part of this study are available from the University of Bristol’s Research Data Repository (http://data.bris.ac.uk/data/), at: https://data.bris.ac.uk/data/dataset/39m1awtj2tjtu2ugpbq5lg12hx^81^. Loneliness: GWAS summary statistics excluding 23andMe can be downloaded from https://t.co/ARgS84uwKl. Data for the top 10,000 genetic variants including 23andMe are available from the authors at request. Coronary artery disease (CAD): GWAS summary statistics are available here: http://ftp.ebi.ac.uk/pub/databases/gwas/summary_statistics/GCST90132001-GCST90133000/GCST90132314/ with accession GCST90132314. Heart failure: GWAS summary statistics are available here: https://cvd.hugeamp.org/dinspector.html?dataset=GWAS_HERMES_eu. Stroke: Details on the GWAS can be found here: https://www.megastroke.org/. For the European subset we contacted the authors of the paper. Systolic blood pressure: GWAS summary statistics are available here: https://www.ebi.ac.uk/gwas/publications/30224653 with accession GCST006624. Type 2 diabetes: GWAS summary statistics are available on request from dbGaP (T2D: Phs001672; Pha004945; T2D.EUR.MVP_Penn_DIAMANTE_Malmo.NatGen2020). Suicide attempts: The GWAS summary statistics can be requested here: https://docs.google.com/forms/d/e/1FAIpQLSc4hGOJ181WGfP3yTjMxUmdf4d6hK0dkXSwRO0ivcPdZIOslg/viewform. Depression: The GWAS summary statistics are available to download from the Psychiatric Genomics Consortium (PGC) (https://figshare.com/articles/dataset/mdd2018/14672085) after agreeing to abide by their data access conditions. Anxiety: The GWAS summary statistics are available to download from the PGC after agreeing to abide by their data access conditions. Wellbeing: The GWAS summary statistics excluding 23andMe are available to download from: https://www.ebi.ac.uk/gwas/publications/30643256.

## Code availability

Analysis code is available from the University of Bristol’s Research Data Repository (http://data.bris.ac.uk/data/), at: https://data.bris.ac.uk/data/dataset/39m1awtj2tjtu2ugpbq5lg12hx^81^.
