# Supplementary material for: Investigating relationships between loneliness, social isolation and health
Source: Nat Commun. 2026 Jul 15;17:5840. doi: 10.1038/s41467-026-74758-7 (PMC13373220; doi:10.1038/s41467-026-74758-7)
Supplement: Supplementary file 1 — Supplementary Information [file 41467_2026_74758_MOESM1_ESM.pdf]

Supplementary Information

Table of Contents

Supplementary Methods 3
1. Deviations from pre-registered analysis plan 3
2. Use of genetic data in UK Biobank 3
3. Genome wide association study (GWAS) of social isolation 4
4. Cox proportional hazards models 4
5. Additional sensitivity analyses 5
6. Details for identifying UK Biobank siblings 5
7. One sample Mendelian Randomisation details and sensitivity analyses 6
8. Two sample Mendelian Randomisation details and sensitivity analyses 6
9. Multivariable MR analyses 8

Supplementary Tables 10
Table S1. Genome-wide association study (GWAS) data for Mendelian Randomisation (MR) analyses 10
Table S2. Phenotypic data in UK Biobank for observational/sibling control analyses and one sample Mendelian Randomisation analyses 14
Table S3. Independent genome-wide significant genetic variants from social isolation[a] genome-wide association study in UK Biobank 21
Table S4. Potential confounders in UK Biobank for observational/sibling control analyses and one sample Mendelian Randomisation analyses 22
Table S5. Observational analysis results 24
Table S6. Observational sensitivity analysis results 37
Table S7. Observational analyses for social isolation measure; sensitivity analysis excluding children 38
Table S8. Sibling control analysis results 43
Table S9. One-sample Mendelian Randomisation analysis results from split sample approach 52
Table S10. One-sample Mendelian Randomisation analysis results from unweighted loneliness approach 56
Table S11. One-sample Mendelian Randomisation analysis sensitivity analyses 57
Table S12. I-squared values for Two-sample Mendelian Randomisation analyses 61
Table S13. Two-sample Mendelian Randomisation analysis results with loneliness and social isolation as exposures 63
Table S14. Two-sample Mendelian Randomisation analysis results with loneliness and social isolation as outcomes 91

Table S15. Multivariable Mendelian Randomisation analysis results with loneliness and social isolation as exposures 119
Supplementary Figures 128
Figure S1. (a) Manhattan plot and (b) Quantile-Quantile plot from social isolation GWAS (N= 457,086) 128
Supplementary Notes 129
STROBE-MR checklist of recommended items to address in reports of Mendelian randomization studies 129
References 141

Supplementary Methods

1. Deviations from pre-registered analysis plan

The analyses conducted deviated slightly from our pre-registered analysis plan (https://doi.org/10.17605/OSF.IO/GWPNY). These are listed below with the reason for the deviation.

- We did not conduct the Cox proportional hazards models using a piecewise regression or similar to examine the relationships between loneliness/social isolation and outcomes of coronary artery disease (CAD), heart failure, stroke and death over time. The main Cox proportional hazards models we conducted did not provide evidence of effects on these outcomes and therefore there was no clear value in conducting further analyses.
- We did not conduct multivariable Mendelian Randomisation (MVMR) within a one-sample Mendelian Randomisation (1SMR) context. This was due to a lack of strong evidence for effects with social isolation and the two-sample Mendelian Randomisation (2SMR) MVMR not revealing any strong results for social isolation.
- We did not conduct mediation MR. In our pre-registration we stated that we would prioritise physical health outcomes with mediation analyses, but given our lack of findings for physical health outcomes we did not conduct the mediation MR analyses.

2. Use of genetic data in UK Biobank

In UK Biobank there are 488,377 participants genotyped samples (49,979 genotyped using the UK BiLEVE array and 438,398 using the UK Biobank Axiom® Array). Pre-imputation quality control, phasing and imputation have been described elsewhere [1] and were already conducted on the data. To summarise multiallelic genetic variants and those with a minor allele frequency (MAF) ≤1% were removed. Phasing of genotype data was performed using a modified version of the SHAPEIT2 algorithm. Genetic variants were imputed to the Haplotype Reference Consortium (HRC) reference panel, using IMPUTE2 algorithms. A graded filtering with different imputation qualities for different MAF ranges was used (Info>0.3 for MAF>3%, Info>0.6 for MAF 1-3%, Info>0.8 for MAF 0.5-1% and Info>0.9 for MAF 0.1-0.5%), where MAF and info scores were recalculated on an in-house derived 'European' subset. Individuals with sex-mismatch or sex-chromosome aneuploidy were excluded. In-house quality control filtering of the UK Biobank data has been described in a published protocol [2]. In addition, we restricted our sample for 1SMR analyses to include only individuals who self-reported as 'White' and 'British' and who had very similar genetic ancestry based on a principal components analysis of genotypes. Self-reported responses were from touchscreen questionnaire questions asking, 'What is your ethnic group?' (response options: White; Mixed; Asian or Asian British; Black or

Black British, Chinese, Other ethnic group, Do not know; Prefer not to answer). If they selected 'White' then they were asked 'What is your ethnic background?' (response options: British; Irish; Any other white background; Prefer not to answer). We acknowledge that ethnicity is a complex social construct with different meanings across different contexts, and is distinct to, although often overlapping with, genetic ancestry [3]. This was done to ensure that the UK Biobank sample was similar in ancestry to the GWAS samples to avoid any bias in the results due to potential differences in associations by ancestry. Previously estimated kinship coefficients using the KING toolset [4] identified 107,162 pairs of related individuals (third degree or closer). We used this as a basis to remove related individuals in all UK Biobank observational, sibling control and 1SMR analyses as described in the published protocol [2].

3. Genome wide association study (GWAS) of social isolation

We conducted our social isolation GWAS using the MRC-IEU UK Biobank GWAS pipeline for 457,086 participants. We used BOLT-LMM software within the pipeline to conduct our GWAS, which used a linear mixed model accounting for relatedness and population stratification and restricted to individuals of 'European' ancestry. We adjusted for sex, genotyping chip, age and assessment centre. Filtering and quality control steps applied to the genetic data are described in **Supplementary Information Section 2** i.e., multiallelic genetic variants or those with MAF ≤1% were removed and rarer genetic variants were required to have a higher imputation INFO score (Info>0.3 for MAF >3%; Info>0.6 for MAF 1-3%; Info>0.8 for MAF 0.5-1%; Info>0.9 for MAF 0.1-0.5%). Therefore, no additional filtering was applied after the GWAS was conducted. To obtain a set of genome-wide significant genetic variants to use in our analyses we restricted the data to those with a p-value $<5x10^{-08}$, resulting in 1,534 genetic variants. We then clumped the data using the function in the 'TwoSampleMR' R package using an r2 of 0.001, window of 10,000 kb and the European 1000 genomes reference panel, resulting in 15 genetic variants to be used in subsequent analyses. A Manhattan plot and a Quantile-Quantile (QQ) plot of our GWAS is shown in **Supplementary Inforamtion Figure S1**. The QQ plot and lambda value of 1.2 suggests there may be some population structure. However, we examined the linkage disequilibrium score intercept and ratio, where a value close to 1 for the intercept and a value close to 0 for the ratio indicates that any inflation is unlikely to be due to causes other than polygenic heritability (e.g., population structure. Our intercept value was 1.04 and our ratio value was 0.11, both reasonably close to 1 and 0, respectively. Details of the 15 genetic variants used in analyses are provided in **Supplementary Information Table S3.**

4. Cox proportional hazards models

Cox proportional hazards models were used with the event defined as all-cause mortality or as incident CAD, heart failure or stroke. Follow-up began at the date of attending the assessment centre

(between 13th March 2006 and 1st October 2010) and individuals who had the event in question before this date were excluded. Follow-up ended when a person had the event, when they died (if the event was not death) or on 30/09/2021; whichever came first. The latter date is the end of availability of hospital episode statistics data in our data download from UK Biobank. Age was used as the time axis. We tested the proportional hazards assumption for each model using the cox.zph() function in the Survival R package.

5. Additional sensitivity analyses

Results for continuous outcomes were reported using robust standard errors, calculated using the 'coeftest' function from the 'lmtest' package in R. We also conducted sensitivity analyses for the outcome hospital admissions, depression trait and anxiety trait, where data were positively skewed, to assess whether alternative modelling approaches impacted the results. To assess this, we conducted sensitivity analyses using both Gamma and negative binomial models for the fully adjusted models. Finally, we also conducted additional analyses using a slightly different measure for social isolation. In this measure we attempted to account for number of children living in the household in case having children living in the home impacted the relationship between social isolation and health outcomes. To do this we used data on the number of children the person had given birth to (UK Biobank field ID: 2734) or fathered (UK Biobank field ID: 2405) and whether that person indicated that a child lived in their household (UK Biobank field ID: 6141). We subtracted the number of children the person had given birth to or fathered from the total household count if they indicated that a child lived at home. However, we ensured that this did not reduce the number of household members below the count of other people living in the household as reported (UK Biobank field ID: 6141).

We also report robust standard errors, calculated using the 'coeftest' function from the 'lmtest' package in R.

6. Details for identifying UK Biobank siblings

Siblings were defined as participants with a kinship coefficient (the probability that two alleles sampled at random from two individuals are identical by descent) between the probability of zero identical-by-state multiplied by –21 + 0.5 and 0.7, and a probability of zero identical-by-state (IBS0) (meaning there is a probability that the siblings have zero alleles in common at a particular locus) between 0.001 and 0.008. These parameters were previously determined from visual clustering [5] and are similar to those obtained elsewhere [1,4]. We also removed adoptees by excluding individuals who responded yes to the question "Were you adopted as a child?".

7. One sample Mendelian Randomisation details and sensitivity analyses

For the Cox Proportional hazards models in 1SMR we used the ratio method. In this approach we first used linear regression to estimate a mean difference in the exposure per unit of the genetic instrument, adjusted for covariates, providing the denominator for the ratio method. Then we used a Cox proportional hazards model to estimate the log(HR) of the outcome per unit of the genetic instrument, adjusted for covariates, providing the numerator for the ratio method. To get the MR estimate of the log(HR) we divided the numerator by the denominator. To obtain 95% confidence intervals we used the Taylor series formula [6].

When creating the genetic scores in the split sample approach, due to the low number of genome-wide significant genetic variants in each half sample for both loneliness and social isolation we used genetic variants and weights with a p-value threshold of $1x10^{-05}$ to create genetic scores in the other half of the sample. Using these genetic scores, we conducted our 1SMR analyses in each half and meta-analysed the results (the estimates from the 1SMR in each half of the data) using the 'meta' package in R (with the metagen() function for a common/fixed effects model) [7]. We also conducted sensitivity analyses for the 1SMR approach using weighted median and weighted mode analyses. To do this, in each half of the sample, we generated summary statistics for each of the outcomes by regressing the exposure genetic variant genotypes onto the MR outcome phenotypes, adjusting for age, sex, the first 20 PCs, assessment centre and genotyping chip. We then used the effect estimates from these regressions for the outcome along with the split sample GWAS output for the exposure as summary statistics for the weighted median and weighted mode sensitivity MR analyses using the 'TwoSampleMR' R package [8,9]. Where loneliness was our exposure, as this was partially overlapping with UK Biobank data, we also conducted 1SMR analyses using genetic variants from the published GWAS data but in unweighted genetic scores (i.e., all alleles assigned a weight of one) in an attempt to remove any bias from the weights being created in a largely overlapping sample. Using unweighted scores however assumes all genetic variants have equal influence and therefore we conducted these alongside the split sample analyses as an additional sensitivity analysis with the loneliness exposure. This additional analysis was not conducted for social isolation due to full sample overlap.

8. Two sample Mendelian Randomisation details and sensitivity analyses

Prior to analyses, as per guidance for the TwoSampleMR R package, we excluded genetic variants in linkage disequilibrium (LD) (using an r^2 of 0.001, window of 10,000 kb and the European 1000 genomes reference panel). If the resulting exposure genetic variants were unavailable in the

outcome GWAS data we attempted to identify proxy genetic variants using LDlink [10] at an LD r^2 threshold of >0.8. Once the final genetic variant set was established we harmonised the exposure and outcome data so that the genetic variant effects for exposure and outcome were all in the same direction using the TwoSampleMR harmonise_data() function, which attempted to infer the positive strand based on allele frequencies and aligned palindromic genetic variants using allele frequencies where possible (or excluded them if it was unable to do this).

For the main analyses we used an approach called the inverse-variance weighted (IVW) method, which assumes that there is no bias due to horizontal pleiotropy (i.e., where genetic variants influence the outcome via pathways other than through the exposure) by fitting a regression for the genetic variant-exposure and genetic variant-outcome associations and constraining the intercept to pass through zero. We also conducted sensitivity analyses to assess the assumptions of 2SMR. These were MR-Egger [11], weighted median [12], MR using robust adjusted profile score (MR-RAPS) [13], MR Pleiotropy RESidual Sum and Outlier (MR-PRESSO) [14], MR lap [15] and MR-Causal Analysis Using Summary Effect estimates (CAUSE) [16]. The MR-Egger estimate is valid even if horizontal pleiotropy is present, under the assumption that the pleiotropic effect of each genetic variant is independent of the strength of that genetic variant as an instrument, by allowing the intercept to not be constrained and allows the extent of a pleiotropic effect to be estimated if the intercept differs from zero. The weighted median method allows for up to 50% of the weight of the genetic variants used in the genetic instrument to be invalid. The MR-RAPS method uses a profile-likelihood function to downweigh outliers and assumes pleotropic effects are normally distributed and therefore provides an unbiased estimate even if there are weak instruments. The MR-PRESSO method can be used to detect and correct for horizontal pleiotropy by estimating the extent to which any outliers impact the effect estimate. The MR-PRESSO global test detects whether horizontal pleiotropy is present and the distortion test evaluates the distortion between the estimate before and after removal of outliers. MR lap accounts for any sample overlap and reduces bias which may occur when using overlapping samples in the exposure and outcome. MR-CAUSE can model both uncorrelated and correlated horizontal pleiotropy, avoiding false positives that may result from correlated pleiotropy. Correlated pleiotropy can occur where genetic variants influence both the exposure and outcome, for example via a heritable confounder.

To estimate the strength of our instruments we calculated first stage F-statistics, where a low value may indicate the presence of weak instruments that could bias results [12,17]. All MR methods (other than MR RAPS) assume that all genetic variants are strong instruments (i.e., there is no measurement

error in the genetic variant-exposure estimates) [12], known as the 'NO Measurement Error' (NOME) assumption. The extent to which the NOME assumption may be violated can be estimated using the F-statistic and the regression dilution I-squared statistics (in relation to MR-Egger), where lower values indicate greater violation. If violated, the IVW estimate may be prone to weak instrument bias and the MR-Egger estimate may be biased towards the null. We assessed the extent of any violation of the NOME assumption using the I-squared values, where an I-squared of less than 0.9 indicated that the MR-Egger estimates should be interpreted with caution due to regression dilution. Where this was the case, we additionally conducted simulation extrapolation (SIMEX) corrections as a sensitivity analysis. The SIMEX approach is a bias adjustment method that can be used when the NOME assumption may be violated, providing an estimate for the case where NOME has been satisfied. When the I-squared is less than 0.6 the SIMEX analysis may also be unreliable. Where loneliness and social isolation were the outcomes, for the exposures we used the genetic variants reported as genome-wide significant in the original publication, even when the full sample was not used (e.g., due to restrictions on the data available for depression and the three wellbeing GWAS, see **Supplementary Information Table S1**), but weights of these genetic variants in the sub-sample were used. In cases where there were less than 5 genetic variants we used a less strict p-value threshold ($1x10^{-06}$ where suicide attempt was the exposure and $1x10^{-05}$ where anxiety was the exposure). Finally, we conducted Steiger filtering [18] to further infer the most likely causal direction. This is achieved by calculating the variance explained in both the exposure and outcome by the genetic variants included in the instrument and assessing whether more variance is explained in the exposure than the outcome. Here we calculate the percentage of genetic variants that explain more variance in the exposure than the outcome. However, Steiger filtering is limited, in that measurement error, if present, can influence the inferred causal direction and provide inaccurate results, thus caution should be taken when interpreting these results.

9. Multivariable MR analyses

MVMR is an extension of MR that can be used to estimate the potential causal effects of multiple exposures (here loneliness and social isolation) on an outcome and assess whether effects of each exposure (conditioning on the other) are independent. In order to do this data for associations of each genetic variant in the instrument with each of the exposures was required. Analyses were conducted using the 'MVMR' package in R [19] using a 2SMR approach. Conditional F-statistics were also calculated with this R package. The main MVMR analysis was the IVW approach. We also conducted sensitivity analyses which included MR Egger, oriented on each exposure (i.e., ensuring the betas are positive for that exposure) to correct for measured and unmeasured horizontal

pleiotropy and MR-PRESSO (raw and outlier corrected) to detect outliers and correct for horizontal pleiotropy via removal of outliers. MR PRESSO also included the global and distortion tests, similar to in the main two-sample MR analyses. Finally, we included a pleiotropy test which measures SNP heterogeneity, which can indicate pleiotropy.

Supplementary Tables

Table S1. Genome-wide association study (GWAS) data for Mendelian Randomisation (MR) analyses

Phenotype	Description	Author	Number of individuals	Number of independent genome-wide significant genetic variants
Exposures				
Loneliness	A binary measure of loneliness was used for one of the cohorts (UKBB) and a continuous measures used for the other 6 cohorts. The binary measure was from a question asking 'Do you feel lonely?'. The continuous measure used varied across cohorts with 2 including a 3-item questionnaire[a], 3 asking 'Did you feel lonely during the past week' with a 4-point scale and another including a 9-item questionnaire[b].	Abdellaoui et al., 2019 [20]	573,604 obtained from 7 different cohorts (including UK Biobank). Where loneliness was the outcome 23andMe data were excluded (N=20,591) as only the top 10,000 genetic variants are available in the full sample.	16
Social isolation	We used data from a GWAS we have conducted in UK Biobank using a combination of two measures (same as in observational analyses):<br><br>1) Frequency of friend/family visits ("How often do you visit friends or family or have them visit you?"). Participants could respond with the following options: almost daily; 2-4 times a week; about once a week; about once a month; once every few months; never or almost never; no friends/family outside household. We combined the last two into the same category and applied the following values to each (with a higher value reflecting greater social isolation): 0, 0.2, 0.4, 0.6, 0.8 and 1, respectively.<br><br>2) Number of people in household ("Including yourself, how many people are living together in your household? (Include those who	NA – see Supplementary Information Section 2	457,086 from UK Biobank	15

Phenotype	Description	Author	Number of individuals	Number of independent genome-wide significant genetic variants
	usually live in the house such as students living away from home during term, partners in the armed forces or professions such as pilots)". Participants responded by providing a number. Answers less than one or more than 100 were rejected. Participants responding with an answer over 12 were asked to confirm. We coded responses as follows in a similar manner to the measure above: 1 in household (i.e., lived alone) = 1, 2 in household = 0.75, 3 in household = 0.5, 4 in household = 0.25, 5+ in household = 0.<br><br>We summed values for each measure to create a combined measure of social isolation.<br><br>Details of the GWAS are provided in the Supplementary Information.			
Physical health outcomes				
Coronary artery disease (CAD)	We used data from a predominantly European GWAS of CAD (from a mixture of sources including ICD9/10 codes and hospital records).	Aragam et al., 2022 [21]	Meta-analysis of 181,522 cases and 1,165,690 controls across 10 cohorts (including UK Biobank)	241
Heart failure	We used data from a European GWAS of heart failure. Cases of heart failure included participants with a clinical diagnosis of heart failure, of any aetiology. Definitions across the different studies included a combination of incidence and prevalence cases and are described in further detail in the GWAS paper.	Shah et al., 2020 [22]	47,309 cases and 930,014 across 26 cohorts (including UK Biobank)	11
Stroke	We used data from a GWAS of 'any stroke' which included ischaemic stroke, stroke of unknown or undetermined type, any ischaemic stroke regardless of subtype and ischaemic stroke subtypes.	Mishra et al., 2022 [23]	73,652 cases and 1,234,808 controls from 44 European cohorts	29 in European sample

Phenotype	Description	Author	Number of individuals	Number of independent genome-wide significant genetic variants
			(including UK Biobank)	
Systolic blood pressure	We used data from a European GWAS of systolic blood (details of the individual cohort measurements can be found in the GWAS paper).	Evangelou et al., 2018 [24]	Meta-analysis of 757,601 participants across 77 cohorts (including UK Biobank)	130
Type 2 diabetes	Case-control for type 2 diabetes diagnosis.	Vujkovic et al., 2020 [25]	148,726 cases and 965,732 controls of European ancestry (including UK Biobank)	425 in European sample
Mental health outcomes				
Suicide attempts	Case-control for suicide attempt in European subsample.	Mullins et al., 2022 [26]	26,590 cases and 492,022 controls (European sample) from the International Suicide Genetics Consortium (ISGC) (including UK Biobank)	2 in European sample
Depression	Case-control for major depressive disorder in a European sample GWAS.	Wray et al., 2018 [27]	45,396 cases and 97,250 controls (excluding 23andMe and UK Biobank)	44
Anxiety	Used data from five core anxiety disorders: generalised anxiety disorder, panic disorder, social phobia, agoraphobia and specific phobias. From this, a case-control phenotype was derived and used in a European sample GWAS.	Otowa et al., 2016 [28]	5,761 cases and 11,765 controls from 7 cohorts (including some sample overlap with loneliness GWAS)	1

Phenotype	Description	Author	Number of individuals	Number of independent genome-wide significant genetic variants
Wellbeing	Three GWAS were used (from European sample):<br>1) Positive affect<br>2) Life satisfaction<br>3) Wellbeing spectrum encompassing measures of life satisfaction, positive affect, neuroticism and depressive symptoms (N GWAMA version).	Baselmans et al., [29]	2,083,151 observations excluding 23andMe	1) 191<br>2) 148<br>3) 231

[a]3-item questionnaire asked the following with a. 3-point scale: 1) How often do you feel left out?, 2) How often do you feel isolated from others?, 3) How often do you feel that you lack companionship?

[b]9-item questionnaire asked the following with a 4-point scale response: 1) I feel in tune with the people around me, 2) There are people I can turn to, 3) I feel alone, 4) I feel part of a group of friends, 5) I have a lot in common with the people around me, 6) I feel isolated from others, 7) There are people who really understand me, 8) I am unhappy being so withdrawn, 9) There are people I can talk to.

Table S2. Phenotypic data in UK Biobank for observational/sibling control analyses and one sample Mendelian Randomisation analyses

Phenotype	Description	UK Biobank codes	Timepoint
Exposures			
Loneliness	Asked "Do you often feel lonely?" (Yes/No). Could also respond with do not know or prefer not to answer.	2020	Baseline between 2006 and 2010
Social isolation	We used data from two measures:<br><br>1) Frequency of friend/family visits ("How often do you visit friends or family or have them visit you?"). Participants could respond with the following options: almost daily; 2-4 times a week; about once a week; about once a month; once every few months; never or almost never; no friends/family outside household. We combined the last two into the same category and applied the following values to each (with a higher value reflecting greater social isolation): 0, 0.2, 0.4, 0.6, 0.8 and 1, respectively.<br><br>2) Number of people in household ("Including yourself, how many people are living together in your household? (Include those who usually live in the house such as students living away from home during term, partners in the armed forces or professions such as pilots)". Participants responded by providing a number. Answers less than one or more than 100 were rejected. Participants responding with an answer over 12 were asked to confirm. We coded responses as follows in a similar manner to the measure above: 1 in household (i.e., lived alone) = 1, 2 in household = 0.75, 3 in household = 0.5, 4 in household = 0.25, 5+ in household = 0.	1031<br><br>709	Baseline between 2006 and 2010

Phenotype	Description	UK Biobank codes	Timepoint
	We summed values for each measure to create a combined measure of social isolation.		
General health outcomes			
Hospital admissions	We also used HES for the number of hospital admissions for each participant in the same follow-up period as the QALYs measure. This was similar to the outcome used by Hazewinkel and colleagues [30].	HES admissions data	Follow-up until 30/09/2021
Quality-adjusted life years (QALYs)	QALYs were estimated based on data from hospital episode statistics (HES) which have been linked to all participants up to 30/09/2021, although we only included data from date of assessment for UK Biobank. This is similar data to that used previously by Harrison and colleagues [31]. Briefly, 240 health conditions (based on a study by Sullivan and colleagues [32] using ICD 9/10 codes) were used to predict QALYs for all participants from recruitment to the aforementioned date or in case of death. A value of 1 is indicative of a full year of perfect health whilst a value of 0 indicates no quality of life or death, although values can also be negative. To ensure consistency with the permitted value range of UK EQ-5D-3L value set, we coded it so that values over 1 were forced to have a value of 1 and values below -0.594 were forced to have a value of -0.594 [33].	HES data using ICD 9 and ICD 10 diagnosis codes for the 240 health conditions	Follow-up until 30/09/2021
Multimorbidity	The presence of two or more self-reported chronic conditions (out of 35 possible conditions). See previous work by North and colleagues for further information about the phenotypes included [34].	Self-reported conditions as per North and colleagues work: alcohol dependency (20002 – 1408); anorexia/bulimia/other eating disorder diagnosis (20002 – 1470); atrial fibrillation (20002 – 1471, 1483); bronchiectasis (20002 – 1114); liver disease and hepatitis (20002 – 1155, 1156, 1157, 1158, 1579, 1580, 1604, 1506); chronic sinusitis (20002 – 1416, 1413, 1417); chronic obstructive pulmonary disease (20002 – 1112,	Baseline between 2006 and 2010

Phenotype	Description	UK Biobank codes	Timepoint
		1113, 1472; and 6152); coronary heart disease (20002 – 1074, 1075, 1079, 1588, 1492, 1591, 1586, 1587; and 6150); dementia/Alzheimer's/cognitive impairment(20002 – 1263); diabetes (20002 – 1220, 1222, 1223, 1276, 1468, 1608; and 2443); diverticulitis (20002 – 1458); hearing loss (20002 – 1420, 1421; 2247; 3393; 2257); heart failure (20002 – 1076); hypertension (20002 – 1065, 1072; and 6150); inflammatory bowel disease (20002 – 1459, 1461, 1462, 1463); multiple sclerosis (20002 – 1261); Parkinson's disease (20002 – 1262); peptic ulcer disease (20002 – 1400, 1457, 1142); peripheral vascular disease (20002 – 1067, 1087); prostate disorders (20002 – 1207, 1396, 1516, 1517); psychoactive substance misuse (20002 – 1409, 1410); rheumatoid arthritis, other inflammatory polyarthropathies and systemic connective tissue disorders (20002 – 1373, 1464, 1381, 1477, 1313, 1377, 1322, 1383, 1480, 1481, 1384, 1372); stroke and transient ischaemic attack (20002 – 1081, 1082, 1583, 1491; and 6150); thyroid disorders (20002 – 1225, 1226, 1522, 1610); constipation (20002 – 1599; and 6154); migraine (20002 – 1265); epilepsy (20002 – 1264); asthma (20002 – 1111; and 6152); irritable bowel syndrome (20002 – 1154); psoriasis or eczema (20002 – 1452, 1453, 1669); anxiety and other neurotic, stress related and somatoform disorders or depression (20002 – 1286, 1287, 1288, 1469, 1615; and 2090; 2100); cancer diagnosis in last 5 years (20001); chronic kidney disease (20002 – 1192, 1193, 1194, 1519, 1607); painful condition (6154); schizophrenia (and other psychosis) or bipolar disorder (20002 – 1289, 1291)	
Mortality	We looked at death as a binary outcome in the same follow-up period as the QALYs measure. Death was coded as occurring for this outcome if there was a date of death recorded. Where there was no date of death recorded, we assumed that this	40000	Follow-up until 30/09/2021

Phenotype	Description	UK Biobank codes	Timepoint
	event had not occurred and the participant was coded as not having this outcome.		
Physical health outcomes			
Coronary artery disease (CAD)	CAD was defined similarly to the measures used in Patel and colleagues [35], but using linked data where we knew the dates of the event after their assessment visit. CAD was defined based on a combination of ICD 10, ICD 9, Office of Population Censuses and Surveys Classification of Interventions and Procedures, version 4 (OPCS4) and mortality data. Those with this event occurring prior to assessment were excluded from analyses (based on the same codes and also self-report data).	HES data using ICD 9 and ICD 10 diagnosis codes and OPCS4 procedure codes and mortality data.	Follow-up until 30/09/2021
Heart failure	Heart failure was defined in a similar manner to that for CAD. Heart failure was defined based on a combination of ICD 10 and ICD 9 codes and mortality data. Those with this event occurring prior to assessment were excluded from analyses (based on the same codes and also self-report data).	HES data using ICD 9 and ICD 10 diagnosis codes and mortality data.	Follow-up until 30/09/2021
Stroke	Stroke was defined in a similar manner to that for CAD. Stroke was defined based on a combination of ICD 10 and ICD 9 codes and mortality data. Those with this event occurring prior to assessment were excluded from analyses (based on the same codes and also self-report data).	HES data using ICD 9 and ICD 10 diagnosis codes and mortality data.	Follow-up until 30/09/2021
Systolic blood pressure	Mean systolic blood pressure (mmHg) was calculated from two readings a few moments apart. These were either automated, manual or a combination of both. If only a single measure was obtained, we used this instead. Automated readings were taken using an Omron 705 IT	4080; 93	Baseline between 2006 and 2010

Phenotype	Description	UK Biobank codes	Timepoint
	electronic blood pressure monitor with a range of 0-255. A manual monitor was used in cases where the automated device could not be used.		
Type 2 Diabetes	A possible or probable diagnosis of type 2 diabetes was used. This was defined as per the Eastwood algorithm. Details on the Eastwood algorithm can be found in the paper describing it [36]. However, to summarise, the algorithm is used to identify diabetes status using a combination of self-report, primary and secondary care data measures within UK Biobank. We used this to identify 'possible' and 'probable' cases of type 2 diabetes, where probable cases have stronger evidence of this being the case from primary care data and possible cases have weaker evidence. We also excluded individuals who had a possible or probable type 1 diabetes as per the Eastwood algorithm.	A combination of variables were used to derive this measure using the Eastwood algorithm: insulin, metformin, sulfonylureas, meglitinides, glitazones, non-metformin oral anti-diabetic, and other oral anti-diabetic medication from the UK Biobank self-reported medications list (20003); gestational diabetes, diabetes not otherwise specified, type 1 diabetes and type 2 diabetes from the self-reported non-cancer illness codes list (20002) and corresponding ages (20009); other diabetes related self-reported measures (2443, 4041; 2976; 6177; 6153; 2986)	Baseline between 2006 and 2010
Mental health outcomes			
Self-harm	Self-harm: Asked "Have you deliberately harmed yourself, whether or not you meant to end your life?". Could respond with no, yes or prefer not to answer. We used yes/no data.	20480	Online questionnaire between 2016 and 2017
Suicide attempts	Suicide attempt: Those you responded yes to the above question were then asked "Have you harmed yourself with the intention to end your life?". Similarly, they could respond with no, yes or prefer not to answer. We used yes/no data.	20483	Online questionnaire between 2016 and 2017
Depression	Binary: Diagnosis of depression obtained from a combination of self-reported data and ICD10 codes.	Binary outcome was obtained from a number of diagnosis variables: depression diagnosis from self-reported non-cancer illness code list (20002 - 1286 variable); ICD 10 codes from primary and secondary diagnoses lists (41202 - F32, F320,	Baseline between 2006 and 2010 and online questionnaire between

Phenotype	Description	UK Biobank codes	Timepoint
		F321, F322, F323, F328, F329, F33, F330, F331, F332, F333, F334, F338, F339 and 41204 – same F codes) and a question in the online mental health questionnaire asking “Have you been diagnosed with one or more of the following mental health problems by a professional, even if you don't have it currently? (tick all that apply)” (20544).	2016 and 2017
	Continuous trait: The Patient Health Questionnaire 9-question version (PHQ-9)	The continuous outcome was obtained from the PHQ-9 (20514; 20510; 20517; 20519; 20511; 20507; 20508; 20518; 20513)	Online questionnaire between 2016 and 2017
Anxiety	Binary: Diagnosis of anxiety (all types) obtained from a combination of self-reported data and ICD10 codes.	Binary outcome was obtained from a number of diagnosis variables: anxiety diagnosis from self-reported non-cancer illness code list (20002 - 1287 variable); ICD 10 codes from primary and secondary diagnoses lists (41202 – F40, F400, F401, F402, F408, F409, F41, F410, F411, F412, F413, F418, F419 and 41204 – same F codes) and a question in the online mental health questionnaire asking “Have you been diagnosed with one or more of the following mental health problems by a professional, even if you don't have it currently? (tick all that apply)” (20544).	Baseline between 2006 and 2010 and online questionnaire between 2016 and 2017
	Continuous trait: The Generalized Anxiety Disorder 7-question version (GAD-7-7)	The continuous outcome was obtained from the GAD-7-7 (20506; 20509; 20520; 20515; 20516; 20505; 20512)	Online questionnaire between 2016 and 2017
Wellbeing	Two measures of wellbeing:<br><br>1) Positive affect* – Asked “In general how happy are you?”. Could respond with extremely happy, very happy, moderately happy, moderately unhappy, very unhappy, extremely unhappy, or they could respond with do not know or prefer not to answer. This was asked at both the initial assessment visit and in an online follow-up. Therefore, we have used data where this is available for a	 <br>4526; 20458	 <br>Baseline between 2006 and 2010 and online questionnaire between 2016 and 2017

Phenotype	Description	UK Biobank codes	Timepoint
	participant from the initial measure and if this is missing then we have used data from the follow-up where data is available.<br><br>2) Meaning in life – Asked “To what extent do you feel your life to be meaningful?”. Could respond with not at all, a little, a moderate amount, very much, an extreme amount or they could respond with do not know or prefer not to answer.	 <br><br><br><br><br>20460	 <br><br><br><br><br>Online questionnaire between 2016 and 2017

**Here we have labelled this measure as positive affect in line with the variables included in the positive affect GWAS, although note that happiness is not the same as positive affect more broadly*

Table S3. Independent genome-wide significant genetic variants from social isolation[a] genome-wide association study in UK Biobank

Genetic variant	Chromosome	Effect allele	Other allele	EAF	Beta	SE	P
rs35393280	20	A	C	0.75	-0.006	0.0008	$1.90x10^{-15}$
rs10099728	8	C	T	0.82	0.007	0.0009	$5.00x10^{-15}$
rs2044725	3	C	T	0.35	-0.005	0.0007	$7.50x10^{-13}$
rs2568955	1	T	C	0.26	-0.005	0.0008	$1.70x10^{-10}$
rs2188541	7	A	G	0.37	0.004	0.0007	$1.00x10^{-09}$
rs4587178	6	T	C	0.62	0.004	0.0007	$3.00x10^{-09}$
rs12155844	8	T	C	0.81	0.005	0.0009	$4.90x10^{-09}$
rs112221661	17	A	G	0.30	0.004	0.0008	$5.50x10^{-09}$
rs1421699	5	G	A	0.35	0.004	0.0007	$7.60x10^{-09}$
rs17499351	5	T	C	0.95	-0.008	0.0015	$1.60x10^{-08}$
rs9907261	17	C	T	0.49	0.004	0.0007	$1.90x10^{-08}$
rs2410941	5	C	A	0.47	-0.004	0.0007	$2.00x10^{-08}$
rs2721940	8	A	C	0.40	-0.004	0.0007	$3.40x10^{-08}$
rs1851919	3	C	T	0.43	0.004	0.0007	$3.80x10^{-08}$
rs6691624	1	C	T	0.34	-0.004	0.0007	$4.30x10^{-08}$

EAF=effect allele frequency, SE=standard error

[a]Social isolation was measured on a scale from 0 to 2 based on household size and number of visits to/from friends or family (Supplementary Table S2)

The genome-wide association study was conducted using BOLT-LMM software using a linear mixed model. Tests were two-tailed and a p-value threshold of $5x10^{-08}$ is indicative of genome-wide significant genetic variants.

Table S4. Potential confounders in UK Biobank for observational/sibling control analyses and one sample Mendelian Randomisation analyses

**Phenotype**	**Description**	**UK Biobank codes**
Age	Derived from date of birth and date of attending assessment centre, rounded to a whole year and modelled as a continuous variable.	21003
Sex	Obtained from NHS records (i.e., sex assigned at birth), but may have been updated by the participant as self-reported sex. (Male or female)	31
Assessment centre	The assessment centre that the participant attended. (Categorical, 22 categories)	54
Townsend deprivation index	Calculated based on postcode from the preceding national census output areas. (Continuous variable)	22189
Education	Asked "Which of the following qualifications do you have?". Could respond with College or University degree; A levels/AS levels or equivalent; O levels/GCSEs or equivalent; CSEs or equivalent; NVQ or HND or HNC or equivalent; Other professional qualifications e.g., nursing, teaching; None of the above; Prefer not to answer. They could select multiple responses.<br><br>A/AS levels are UK subject specific qualifications typically completed over 2 years between the ages of 16 and 18. 'GCSEs (General Certificate of Secondary Education) or O levels are UK subject specific qualifications typically completed over 3 years towards the end of secondary school education.<br><br>We converted this to years of education as per Okbay et al [37] and modelled it as a continuous variable.	6138
Income	Asked "What is the average total income before tax received by your household?". Could respond with less than 18,000; 18,000 to 30,999; 31,000 to 51,999; 52,000 to 100,000, greater than 100,000; Do not know; Prefer not to answer.	738
Ethnicity	Self-reported responses were from touchscreen questionnaire questions asking, 'What is your ethnic group?' Could respond: White; Mixed; Asian or Asian British; Black or Black British, Chinese, Other ethnic group, Do not know; Prefer not to answer. Branching questions were then asked from this.<br><br>We coded this as 4 groups.	21000
Disability	Asked "Do you have any long-standing illness, disability or infirmity?" (Yes/No). Could also respond with do not know or prefer not to answer.	2188

Phenotype	Description	UK Biobank codes
Adverse childhood events	In the mental well-being online questionnaire participants were asked each of the following questions:<br><br>1) "When I was growing up: People in my family hit me so hard that it left me with bruises or marks."<br><br>2) "When I was growing up: Someone molested me (sexually)."<br><br>3) "When I was growing up: I felt that someone in my family hated me."<br><br>4) "When I was growing up: I felt loved."<br><br>5) "When I was growing up: There was someone to take me to the doctor if I needed it."<br><br>They could respond: never true; rarely true; sometimes true; often true; very often true; prefer not to answer.<br><br>We combined these into a single measure, summing whether each of the above was present (1 for present, 0 for absent) as per Soares et al[38]. The first three were considered present if they responded as rarely true or above. The fourth was considered present if they reported as never true or rarely true. The fifth was considered present if they responded with sometimes true or below. The combined measure was modelled as a continuous variable.	29077; 29079; 29078; 29076; 29080
Principal components	We used the first 20 principal components (PCs) from PC analysis of genotype data. (Continuous variables)	Derived as described previously [2].
Genotyping chip	Two genotyping chips were used in UK Biobank; the UKBB axiom array (90% of participants were genotyped with this) and the UK BiLEVE array. (Categorical, 2 categories)	22000

Table S5. Observational analysis results

Exposure	Outcome	Model	Level of adjustment	N	HR, OR or mean difference (95% CI)	p-value	Proportional hazards test (CoxPH)
Loneliness	CAD	CoxPH	unadjusted	392,132	1.27 (1.23 to 1.31)	9.77e-57	5.06e-11
Loneliness	CAD	CoxPH	adjusted1[a]	321,985	1.26 (1.21 to 1.30)	1.86e-39	2.07e-05
Loneliness	CAD	CoxPH	adjusted1 with disability[a]	315,593	1.20 (1.16 to 1.24)	4.59e-24	1.65e-04
Loneliness	CAD	CoxPH	adjusted1 with ACEs[a]	117,412	1.14 (1.06 to 1.23)	7.81e-04	0.37
Loneliness	CAD	CoxPH	adjusted2[a]	115,557	1.10 (1.02 to 1.19)	0.02	0.61
Loneliness	HF	CoxPH	unadjusted	412,220	1.54 (1.48 to 1.60)	8.89e-93	5.13e-08
Loneliness	HF	CoxPH	adjusted1[a]	337,606	1.52 (1.45 to 1.59)	3.65e-64	6.94e-05
Loneliness	HF	CoxPH	adjusted1 with disability[a]	330,811	1.40 (1.34 to 1.47)	2.11e-41	9.19e-04
Loneliness	HF	CoxPH	adjusted1 with ACEs[a]	120,617	1.40 (1.21 to 1.61)	5.75e-06	0.68
Loneliness	HF	CoxPH	adjusted2[a]	118,693	1.29 (1.12 to 1.50)	6.34e-04	0.53
Loneliness	Stroke	CoxPH	unadjusted	407,082	1.36 (1.29 to 1.44)	3.78e-30	8.37e-04
Loneliness	Stroke	CoxPH	adjusted1[a]	333,578	1.37 (1.28 to 1.45)	1.09e-22	8.51e-03
Loneliness	Stroke	CoxPH	adjusted1 with disability[a]	326,922	1.32 (1.24 to 1.41)	4.18e-18	0.03
Loneliness	Stroke	CoxPH	adjusted1 with ACEs[a]	119,791	1.13 (0.95 to 1.36)	0.17	0.23
Loneliness	Stroke	CoxPH	adjusted2[a]	117,889	1.11 (0.92 to 1.33)	0.29	0.27

Exposure	Outcome	Model	Level of adjustment	N	HR, OR or mean difference (95% CI)	p-value	Proportional hazards test (CoxPH)
Loneliness	Death	CoxPH	unadjusted	414,431	1.44 (1.40 to 1.48)	6.77e-153	1.53e-15
Loneliness	Death	CoxPH	adjusted1[a]	339,324	1.41 (1.36 to 1.45)	7.27e-98	7.84e-10
Loneliness	Death	CoxPH	adjusted2[a,b]	332,507	1.34 (1.30 to 1.39)	1.03e-70	9.99e-08
Loneliness	Multimorbidity	logistic	unadjusted	414,329	2.25 (2.21 to 2.29)	0.00	
Loneliness	Multimorbidity	logistic	adjusted1	339,253	2.14 (2.10 to 2.18)	0.00	
Loneliness	Multimorbidity	logistic	adjusted1 with disability	332,439	1.98 (1.94 to 2.02)	0.00	
Loneliness	Multimorbidity	logistic	adjusted1 with ACEs	120,875	1.94 (1.87 to 2.00)	0.00	
Loneliness	Multimorbidity	logistic	adjusted2	118,947	1.85 (1.78 to 1.91)	7.30e-250	
Loneliness	T2D	logistic	unadjusted	414,432	1.45 (1.40 to 1.49)	3.25e-102	
Loneliness	T2D	logistic	adjusted1	339,325	1.41 (1.35 to 1.46)	2.92e-61	
Loneliness	T2D	logistic	adjusted1 with disability	332,508	1.15 (1.10 to 1.20)	2.43e-10	
Loneliness	T2D	logistic	adjusted1 with ACEs	120,893	1.25 (1.13 to 1.38)	1.25e-05	
Loneliness	T2D	logistic	adjusted2	118,965	1.04 (0.93 to 1.15)	0.50	
Loneliness	Hospital admissions	linear	unadjusted	414,432	0.86 (0.74 to 0.97)	2.52e-48	
Loneliness	Hospital admissions	linear	adjusted1	339,325	0.70 (0.58 to 0.81)	8.52e-33	
Loneliness	Hospital admissions	linear	adjusted1 with disability	332,508	0.44 (0.32 to 0.55)	5.46e-13	
Loneliness	Hospital admissions	linear	adjusted1 with ACEs	120,893	0.44 (0.34 to 0.55)	9.61e-17	

Exposure	Outcome	Model	Level of adjustment	N	HR, OR or mean difference (95% CI)	p-value	Proportional hazards test (CoxPH)
Loneliness	Hospital admissions	linear	adjusted2	118,965	0.33 (0.22 to 0.43)	7.98e-10	
Loneliness	SBP	linear	unadjusted	413,588	-2.92 (-3.06 to -2.77)	0.00	
Loneliness	SBP	linear	adjusted1	338,736	-1.61 (-1.76 to -1.45)	1.85e-94	
Loneliness	SBP	linear	adjusted1 with disability	331,948	-1.55 (-1.71 to -1.40)	2.76e-85	
Loneliness	SBP	linear	adjusted1 with ACEs	120,801	-1.54 (-1.81 to -1.28)	2.79e-30	
Loneliness	SBP	linear	adjusted2	118,876	-1.55 (-1.82 to -1.28)	7.57e-30	
Loneliness	QALYs	linear	unadjusted	336,799	-0.05 (-0.05 to -0.05)	0.00	
Loneliness	QALYs	linear	adjusted1	273,910	-0.04 (-0.04 to -0.04)	1.98e-323	
Loneliness	QALYs	linear	adjusted1 with disability	268,055	-0.03 (-0.03 to -0.02)	4.04e-166	
Loneliness	QALYs	linear	adjusted1 with ACEs	94,950	-0.02 (-0.02 to -0.02)	9.45e-59	
Loneliness	QALYs	linear	adjusted2	93,307	-0.01 (-0.02 to -0.01)	3.72e-32	
Loneliness	Self-harm	logistic	unadjusted	131,523	3.01 (2.85 to 3.19)	0.00	
Loneliness	Self-harm	logistic	adjusted1	114,019	2.43 (2.28 to 2.58)	5.38e-171	
Loneliness	Self-harm	logistic	adjusted1 with disability	112,132	2.28 (2.14 to 2.43)	1.27e-141	
Loneliness	Self-harm	logistic	adjusted1 with ACEs	80,247	1.96 (1.81 to 2.12)	8.26e-64	
Loneliness	Self-harm	logistic	adjusted2	79,000	1.86 (1.72 to 2.02)	6.33e-53	
Loneliness	Suicide attempt	logistic	unadjusted	131,343	3.38 (3.13 to 3.64)	2.07e-220	

Exposure	Outcome	Model	Level of adjustment	N	HR, OR or mean difference (95% CI)	p-value	Proportional hazards test (CoxPH)
Loneliness	Suicide attempt	logistic	adjusted1	113,863	2.62 (2.41 to 2.84)	9.77e-114	
Loneliness	Suicide attempt	logistic	adjusted1 with disability	111,982	2.41 (2.21 to 2.62)	2.92e-91	
Loneliness	Suicide attempt	logistic	adjusted1 with ACEs	80,147	2.02 (1.82 to 2.25)	1.29e-38	
Loneliness	Suicide attempt	logistic	adjusted2	78,904	1.89 (1.70 to 2.11)	8.76e-31	
Loneliness	Depression diagnosis	logistic	unadjusted	414,432	3.15 (3.09 to 3.21)	0.00	
Loneliness	Depression diagnosis	logistic	adjusted1	339,325	2.79 (2.73 to 2.85)	0.00	
Loneliness	Depression diagnosis	logistic	adjusted1 with disability	332,508	2.63 (2.57 to 2.69)	0.00	
Loneliness	Depression diagnosis	logistic	adjusted1 with ACEs	120,893	2.63 (2.53 to 2.72)	0.00	
Loneliness	Depression diagnosis	logistic	adjusted2	118,965	2.54 (2.44 to 2.63)	0.00	
Loneliness	Anxiety diagnosis	logistic	unadjusted	414,432	2.15 (2.11 to 2.21)	0.00	
Loneliness	Anxiety diagnosis	logistic	adjusted1	339,325	1.97 (1.92 to 2.03)	0.00	
Loneliness	Anxiety diagnosis	logistic	adjusted1 with disability	332,508	1.88 (1.83 to 1.93)	0.00	
Loneliness	Anxiety diagnosis	logistic	adjusted1 with ACEs	120,893	1.96 (1.88 to 2.05)	2.10e-205	
Loneliness	Anxiety diagnosis	logistic	adjusted2	118,965	1.90 (1.82 to 1.98)	1.73e-180	

Exposure	Outcome	Model	Level of adjustment	N	HR, OR or mean difference (95% CI)	p-value	Proportional hazards test (CoxPH)
Loneliness	Depression trait	linear	unadjusted	129,518	2.90 (2.82 to 2.97)	0.00	
Loneliness	Depression trait	linear	adjusted1	112,498	2.58 (2.50 to 2.66)	0.00	
Loneliness	Depression trait	linear	adjusted1 with disability	110,678	2.46 (2.39 to 2.54)	0.00	
Loneliness	Depression trait	linear	adjusted1 with ACEs	79,432	2.23 (2.14 to 2.31)	0.00	
Loneliness	Depression trait	linear	adjusted2	78,213	2.14 (2.05 to 2.22)	0.00	
Loneliness	Anxiety trait	linear	unadjusted	130,109	2.24 (2.17 to 2.30)	0.00	
Loneliness	Anxiety trait	linear	adjusted1	112,969	1.96 (1.89 to 2.03)	0.00	
Loneliness	Anxiety trait	linear	adjusted1 with disability	111,146	1.89 (1.82 to 1.96)	0.00	
Loneliness	Anxiety trait	linear	adjusted1 with ACEs	79,741	1.65 (1.57 to 1.73)	0.00	
Loneliness	Anxiety trait	linear	adjusted2	78,513	1.60 (1.52 to 1.68)	0.00	
Loneliness	Happy	linear	unadjusted	225,943	-0.61 (-0.62 to -0.61)	0.00	
Loneliness	Happy	linear	adjusted1	189,685	-0.57 (-0.58 to -0.56)	0.00	
Loneliness	Happy	linear	adjusted1 with disability	186,126	-0.56 (-0.57 to -0.55)	0.00	
Loneliness	Happy	linear	adjusted1 with ACEs	94,265	-0.53 (-0.54 to -0.51)	0.00	
Loneliness	Happy	linear	adjusted2	92,761	-0.52 (-0.53 to -0.51)	0.00	
Loneliness	Meaning in life	linear	unadjusted	128,845	-0.51 (-0.52 to -0.49)	0.00	
Loneliness	Meaning in life	linear	adjusted1	111,843	-0.48 (-0.49 to -0.46)	0.00	

Exposure	Outcome	Model	Level of adjustment	N	HR, OR or mean difference (95% CI)	p-value	Proportional hazards test (CoxPH)
Loneliness	Meaning in life	linear	adjusted1 with disability	110,019	-0.47 (-0.48 to -0.45)	0.00	
Loneliness	Meaning in life	linear	adjusted1 with ACEs	78,790	-0.44 (-0.46 to -0.42)	0.00	
Loneliness	Meaning in life	linear	adjusted2	77,577	-0.43 (-0.45 to -0.41)	0.00	
Social isolation	CAD	CoxPH	unadjusted	390,606	1.13 (1.09 to 1.18)	5.30e-10	7.28e-03
Social isolation	CAD	CoxPH	adjusted1[a]	325,212	0.95 (0.91 to 0.99)	0.02	0.03
Social isolation	CAD	CoxPH	adjusted1 with disability[a]	318,510	0.94 (0.90 to 0.98)	7.18e-03	0.16
Social isolation	CAD	CoxPH	adjusted1 with ACEs[a]	118,431	0.84 (0.77 to 0.92)	1.14e-04	0.84
Social isolation	CAD	CoxPH	adjusted2[a]	116,512	0.84 (0.77 to 0.92)	1.75e-04	0.50
Social isolation	HF	CoxPH	unadjusted	410,475	1.43 (1.35 to 1.51)	1.83e-32	6.44e-04
Social isolation	HF	CoxPH	adjusted1[a]	341,000	1.15 (1.08 to 1.23)	1.55e-05	0.03
Social isolation	HF	CoxPH	adjusted1 with disability[a]	333,885	1.15 (1.07 to 1.23)	4.06e-05	0.12
Social isolation	HF	CoxPH	adjusted1 with ACEs[a]	121,662	1.01 (0.85 to 1.20)	0.91	0.29

Exposure	Outcome	Model	Level of adjustment	N	HR, OR or mean difference (95% CI)	p-value	Proportional hazards test (CoxPH)
Social isolation	HF	CoxPH	adjusted2[a]	119,673	1.00 (0.84 to 1.19)	0.98	0.45
Social isolation	Stroke	CoxPH	unadjusted	405,408	1.22 (1.13 to 1.31)	7.91e-08	0.02
Social isolation	Stroke	CoxPH	adjusted1[a]	336,937	1.03 (0.95 to 1.12)	0.43	0.20
Social isolation	Stroke	CoxPH	adjusted1 with disability[a]	329,964	1.05 (0.96 to 1.13)	0.29	0.37
Social isolation	Stroke	CoxPH	adjusted1 with ACEs[a]	120,833	0.95 (0.77 to 1.17)	0.64	0.45
Social isolation	Stroke	CoxPH	adjusted2[a]	118,866	0.97 (0.78 to 1.19)	0.74	0.42
Social isolation	Death	CoxPH	unadjusted	412,649	1.69 (1.63 to 1.76)	3.50e-165	3.98e-11
Social isolation	Death	CoxPH	adjusted1[a]	342,733	1.34 (1.29 to 1.40)	1.20e-42	2.59e-05
Social isolation	Death	CoxPH	adjusted2[a,b]	335,593	1.34 (1.29 to 1.40)	1.04e-41	3.30e-03
Social isolation	Multimorbidity	logistic	unadjusted	412,549	1.45 (1.43 to 1.48)	0.00	
Social isolation	Multimorbidity	logistic	adjusted1	342,661	1.07 (1.05 to 1.10)	4.08e-11	
Social isolation	Multimorbidity	logistic	adjusted1 with disability	335,524	1.04 (1.02 to 1.07)	5.76e-04	

Exposure	Outcome	Model	Level of adjustment	N	HR, OR or mean difference (95% CI)	p-value	Proportional hazards test (CoxPH)
Social isolation	Multimorbidity	logistic	adjusted1 with ACEs	121,922	1.06 (1.02 to 1.09)	3.51e-03	
Social isolation	Multimorbidity	logistic	adjusted2	119,929	1.05 (1.01 to 1.09)	0.02	
Social isolation	T2D	logistic	unadjusted	412,650	1.71 (1.64 to 1.78)	5.01e-132	
Social isolation	T2D	logistic	adjusted1	342,734	1.12 (1.06 to 1.18)	1.80e-05	
Social isolation	T2D	logistic	adjusted1 with disability	335,594	1.07 (1.01 to 1.13)	0.01	
Social isolation	T2D	logistic	adjusted1 with ACEs	121,940	1.25 (1.11 to 1.41)	2.24e-04	
Social isolation	T2D	logistic	adjusted2	119,947	1.22 (1.08 to 1.38)	1.56e-03	
Social isolation	Hospital admissions	linear	unadjusted	412,650	1.10 (0.99 to 1.21)	5.07e-85	
Social isolation	Hospital admissions	linear	adjusted1	342,734	-0.12 (-0.25 to 0.02)	0.09	
Social isolation	Hospital admissions	linear	adjusted1 with disability	335,594	-0.19 (-0.32 to -0.05)	5.70e-03	
Social isolation	Hospital admissions	linear	adjusted1 with ACEs	121,940	-0.28 (-0.39 to -0.18)	6.45e-08	
Social isolation	Hospital admissions	linear	adjusted2	119,947	-0.30 (-0.41 to -0.20)	9.19e-09	

Exposure	Outcome	Model	Level of adjustment	N	HR, OR or mean difference (95% CI)	p-value	Proportional hazards test (CoxPH)
Social isolation	SBP	linear	unadjusted	411,812	4.60 (4.43 to 4.77)	0.00	
Social isolation	SBP	linear	adjusted1	342,135	0.11 (-0.07 to 0.29)	0.23	
Social isolation	SBP	linear	adjusted1 with disability	335,027	0.13 (-0.05 to 0.31)	0.14	
Social isolation	SBP	linear	adjusted1 with ACEs	121,844	0.44 (0.15 to 0.73)	2.76e-03	
Social isolation	SBP	linear	adjusted2	119,854	0.46 (0.17 to 0.76)	1.89e-03	
Social isolation	QALYs	linear	unadjusted	334,902	-0.06 (-0.06 to -0.06)	0.00	
Social isolation	QALYs	linear	adjusted1	276,687	-4.44e-03 (-6.58e-03 to -2.31e-03)	4.38e-05	
Social isolation	QALYs	linear	adjusted1 with disability	270,553	-5.16e-04 (-2.56e-03 to 1.53e-03)	0.62	
Social isolation	QALYs	linear	adjusted1 with ACEs	95,783	5.20e-03 (2.72e-03 to 7.68e-03)	4.04e-05	
Social isolation	QALYs	linear	adjusted2	94,086	6.16e-03 (3.79e-03 to 8.54e-03)	3.62e-07	
Social isolation	Self-harm	logistic	unadjusted	131,455	1.49 (1.38 to 1.62)	8.08e-23	
Social isolation	Self-harm	logistic	adjusted1	114,972	1.61 (1.48 to 1.75)	2.06e-28	

Exposure	Outcome	Model	Level of adjustment	N	HR, OR or mean difference (95% CI)	p-value	Proportional hazards test (CoxPH)
Social isolation	Self-harm	logistic	adjusted1 with disability	113,013	1.57 (1.44 to 1.71)	8.27e-25	
Social isolation	Self-harm	logistic	adjusted1 with ACEs	80,890	1.36 (1.23 to 1.50)	4.62e-09	
Social isolation	Self-harm	logistic	adjusted2	79,596	1.33 (1.20 to 1.48)	5.03e-08	
Social isolation	Suicide attempt	logistic	unadjusted	131,270	1.90 (1.70 to 2.12)	8.18e-30	
Social isolation	Suicide attempt	logistic	adjusted1	114,810	1.76 (1.56 to 1.97)	4.21e-21	
Social isolation	Suicide attempt	logistic	adjusted1 with disability	112,857	1.69 (1.50 to 1.90)	3.05e-18	
Social isolation	Suicide attempt	logistic	adjusted1 with ACEs	80,791	1.39 (1.20 to 1.60)	8.34e-06	
Social isolation	Suicide attempt	logistic	adjusted2	79,500	1.35 (1.17 to 1.56)	5.07e-05	
Social isolation	Depression diagnosis	logistic	unadjusted	412,650	1.42 (1.38 to 1.45)	1.13e-151	
Social isolation	Depression diagnosis	logistic	adjusted1	342,734	1.38 (1.34 to 1.42)	4.98e-98	
Social isolation	Depression diagnosis	logistic	adjusted1 with disability	335,594	1.35 (1.31 to 1.39)	7.96e-81	
Social isolation	Depression diagnosis	logistic	adjusted1 with ACEs	121,940	1.24 (1.18 to 1.30)	1.84e-18	

Exposure	Outcome	Model	Level of adjustment	N	HR, OR or mean difference (95% CI)	p-value	Proportional hazards test (CoxPH)
Social isolation	Depression diagnosis	logistic	adjusted2	119,947	1.23 (1.17 to 1.29)	6.06e-17	
Social isolation	Anxiety diagnosis	logistic	unadjusted	412,650	1.30 (1.26 to 1.34)	7.01e-62	
Social isolation	Anxiety diagnosis	logistic	adjusted1	342,734	1.26 (1.22 to 1.31)	2.49e-36	
Social isolation	Anxiety diagnosis	logistic	adjusted1 with disability	335,594	1.24 (1.20 to 1.29)	7.31e-31	
Social isolation	Anxiety diagnosis	logistic	adjusted1 with ACEs	121,940	1.12 (1.06 to 1.19)	6.19e-05	
Social isolation	Anxiety diagnosis	logistic	adjusted2	119,947	1.12 (1.06 to 1.18)	1.31e-04	
Social isolation	Depression trait	linear	unadjusted	129,422	0.27 (0.20 to 0.33)	1.06e-15	
Social isolation	Depression trait	linear	adjusted1	113,391	0.42 (0.35 to 0.49)	1.19e-30	
Social isolation	Depression trait	linear	adjusted1 with disability	111,508	0.38 (0.31 to 0.45)	1.14e-25	
Social isolation	Depression trait	linear	adjusted1 with ACEs	80,040	0.28 (0.20 to 0.36)	5.67e-12	
Social isolation	Depression trait	linear	adjusted2	78,782	0.25 (0.17 to 0.33)	3.85e-10	
Social isolation	Anxiety trait	linear	unadjusted	129,997	-0.07 (-0.13 to -0.01)	0.02	

Exposure	Outcome	Model	Level of adjustment	N	HR, OR or mean difference (95% CI)	p-value	Proportional hazards test (CoxPH)
Social isolation	Anxiety trait	linear	adjusted1	113,874	0.10 (0.04 to 0.16)	2.33e-03	
Social isolation	Anxiety trait	linear	adjusted1 with disability	111,980	0.07 (6.83e-03 to 0.14)	0.03	
Social isolation	Anxiety trait	linear	adjusted1 with ACEs	80,355	-0.05 (-0.12 to 0.03)	0.21	
Social isolation	Anxiety trait	linear	adjusted2	79,081	-0.06 (-0.14 to 6.86e-03)	0.08	
Social isolation	Happy	linear	unadjusted	226,040	-0.21 (-0.22 to -0.20)	0.00	
Social isolation	Happy	linear	adjusted1	191,383	-0.22 (-0.23 to -0.21)	0.00	
Social isolation	Happy	linear	adjusted1 with disability	187,679	-0.22 (-0.23 to -0.21)	0.00	
Social isolation	Happy	linear	adjusted1 with ACEs	95,039	-0.16 (-0.18 to -0.15)	4.64e-102	
Social isolation	Happy	linear	adjusted2	93,483	-0.16 (-0.17 to -0.14)	2.27e-97	
Social isolation	Meaning in life	linear	unadjusted	128,746	-0.33 (-0.35 to -0.32)	0.00	
Social isolation	Meaning in life	linear	adjusted1	112,752	-0.32 (-0.34 to -0.31)	0.00	
Social isolation	Meaning in life	linear	adjusted1 with disability	110,865	-0.32 (-0.34 to -0.30)	0.00	

Exposure	Outcome	Model	Level of adjustment	N	HR, OR or mean difference (95% CI)	p-value	Proportional hazards test (CoxPH)
Social isolation	Meaning in life	linear	adjusted1 with ACEs	79,402	-0.30 (-0.32 to -0.28)	2.61e-225	
Social isolation	Meaning in life	linear	adjusted2	78,148	-0.30 (-0.32 to -0.28)	3.90e-220	

HR=hazard ratio, OR=odds ratio, CI=confidence interval, CoxPH=Cox proportional hazards, CAD=coronary artery disease, HF=heart failure, T2D=type 2 diabetes, SBP=systolic blood pressure, QALYs=quality-adjusted life years

Statistical tests used are indicated in the table and all were two-tailed tests.

Adjusted 1: age when attended assessment centre, sex, assessment centre, Townsend deprivation index, education years based on qualifications, household income before tax and ethnicity

Adjusted 2: adjusted 1 as well as long-standing illness/disability/infirmity and a score for adverse childhood experiences (ACEs)

[a] *For all Cox proportional hazards models the covariate 'centre' was not included due to issues with fitting the model*

[b] *For all Cox proportional hazards models with death as an outcome the covariate ACEs was also not included due to issues with fitting the model*

Table S6. Observational sensitivity analysis results

Exposure	Outcome	Model	Level of adjustment	N	Mean difference (95% CI)	p-value
Loneliness	Hospital admissions	Gamma	adjusted2	118,965	0.08 (0.06 to 0.10)	1.90e-12
Loneliness	Hospital admissions	Negative binomial	adjusted2	118,965	0.12 (0.09 to 0.16)	1.09e-13
Loneliness	Depression trait	Gamma	adjusted2	78,213	0.47 (0.46 to 0.49)	0.00
Loneliness	Depression trait	Negative binomial	adjusted2	78,213	0.63 (0.61 to 0.65)	0.00
Loneliness	Anxiety trait	Gamma	adjusted2	78,513	0.43 (0.41 to 0.45)	0.00
Loneliness	Anxiety trait	Negative binomial	adjusted2	78,513	0.62 (0.59 to 0.64)	0.00
Social isolation	Hospital admissions	Gamma	adjusted2	119,947	-0.06 (-0.09 to -0.04)	1.13e-06
Social isolation	Hospital admissions	Negative binomial	adjusted2	119,947	-0.09 (-0.12 to -0.05)	8.77e-06
Social isolation	Depression trait	Gamma	adjusted2	78,782	0.06 (0.04 to 0.08)	1.40e-08
Social isolation	Depression trait	Negative binomial	adjusted2	78,782	0.08 (0.05 to 0.11)	1.86e-08
Social isolation	Anxiety trait	Gamma	adjusted2	79,081	-0.02 (-0.04 to 4.89e-05)	0.05
Social isolation	Anxiety trait	Negative binomial	adjusted2	79,081	-0.03 (-0.07 to 7.64e-04)	0.06

OR=odds ratio, CI=confidence intervals
Statistical tests used are indicated in the table and all were two-tailed tests.
Adjusted 2: age when attended assessment centre, sex, assessment centre, Townsend deprivation index, education years based on qualifications, household income before tax and ethnicity, long-standing illness/disability/infirmity and a score for adverse childhood experiences (ACEs)

Table S7. Observational analyses for social isolation measure; sensitivity analysis excluding children

Exposure	Outcome	Model	Level of adjustment	N	HR, OR or mean difference (95% CI)	p-value	Proportional hazards test (CoxPH)
Social isolation	CAD	CoxPH	unadjusted	390,216	1.11 (1.06 to 1.16)	9.74e-06	0.49
Social isolation	CAD	CoxPH	adjusted1[a]	324,974	0.91 (0.86 to 0.96)	2.31e-04	0.26
Social isolation	CAD	CoxPH	adjusted2[a]	116,463	0.76 (0.68 to 0.85)	1.87e-06	0.68
Social isolation	HF	CoxPH	unadjusted	410,050	1.46 (1.37 to 1.56)	1.08e-28	0.40
Social isolation	HF	CoxPH	adjusted1[a]	340,739	1.16 (1.08 to 1.25)	1.06e-04	0.60
Social isolation	HF	CoxPH	adjusted2[a]	119,621	1.01 (0.82 to 1.25)	0.90	0.26
Social isolation	Stroke	CoxPH	unadjusted	404,985	1.24 (1.14 to 1.35)	5.16e-07	0.27
Social isolation	Stroke	CoxPH	adjusted1[a]	336,679	1.04 (0.95 to 1.15)	0.36	0.37
Social isolation	Stroke	CoxPH	adjusted2[a]	118,815	0.96 (0.74 to 1.23)	0.74	0.64
Social isolation	Death	CoxPH	unadjusted	412,221	1.70 (1.63 to 1.77)	4.71e-128	1.35e-03
Social isolation	Death	CoxPH	adjusted1[a,b]	342,471	1.38 (1.31 to 1.44)	6.88e-39	0.02

Exposure	Outcome	Model	Level of adjustment	N	HR, OR or mean difference (95% CI)	p-value	Proportional hazards test (CoxPH)
Social isolation	Death	CoxPH	adjusted2[a,b]	335,351	1.39 (1.32 to 1.46)	1.93e-40	0.08
Social isolation	Multimorbidity	logistic	unadjusted	412,122	1.04 (1.01 to 1.06)	1.43e-03	
Social isolation	Multimorbidity	logistic	adjusted1	342,399	1.06 (1.03 to 1.09)	4.47e-05	
Social isolation	Multimorbidity	logistic	adjusted2	119,877	1.04 (0.99 to 1.10)	0.10	
Social isolation	T2D	logistic	unadjusted	412,222	1.20 (1.14 to 1.27)	6.73e-11	
Social isolation	T2D	logistic	adjusted1	342,472	1.08 (1.02 to 1.15)	0.01	
Social isolation	T2D	logistic	adjusted2	119,895	1.20 (1.03 to 1.39)	0.02	
Social isolation	Hospital admissions	linear	unadjusted	412,222	-0.34 (-0.49 to -0.19)	7.43e-06	
Social isolation	Hospital admissions	linear	adjusted1	342,472	-0.23 (-0.39 to -0.07)	5.88e-03	
Social isolation	Hospital admissions	linear	adjusted2	119,895	-0.41 (-0.56 to -0.27)	2.18e-08	
Social isolation	SBP	linear	unadjusted	411,404	-2.34 (-2.56 to -2.12)	2.41e-95	
Social isolation	SBP	linear	adjusted1	341,882	-0.82 (-1.05 to -0.59)	3.22e-12	

Exposure	Outcome	Model	Level of adjustment	N	HR, OR or mean difference (95% CI)	p-value	Proportional hazards test (CoxPH)
Social isolation	SBP	linear	adjusted2	119,802	-0.64 (-1.04 to -0.25)	1.40e-03	
Social isolation	QALYs	linear	unadjusted	334,549	6.93e-04 (-2.04e-03 to 3.43e-03)	0.62	
Social isolation	QALYs	linear	adjusted1	276,476	1.40e-03 (-1.45e-03 to 4.24e-03)	0.34	
Social isolation	QALYs	linear	adjusted2	94,045	0.01 (0.01 to 0.02)	4.27e-16	
Social isolation	Self-harm	logistic	unadjusted	131,411	2.44 (2.20 to 2.71)	4.85e-64	
Social isolation	Self-harm	logistic	adjusted1	114,932	1.80 (1.61 to 2.01)	2.44e-24	
Social isolation	Self-harm	logistic	adjusted2	79,574	1.36 (1.19 to 1.57)	1.28e-05	
Social isolation	Suicide attempt	logistic	unadjusted	131,226	2.67 (2.32 to 3.07)	3.51e-42	
Social isolation	Suicide attempt	logistic	adjusted1	114,770	1.84 (1.58 to 2.14)	5.03e-15	
Social isolation	Suicide attempt	logistic	adjusted2	79,478	1.24 (1.03 to 1.50)	0.03	
Social isolation	Depression diagnosis	logistic	unadjusted	412,222	1.52 (1.47 to 1.57)	1.98e-132	
Social isolation	Depression diagnosis	logistic	adjusted1	342,472	1.40 (1.34 to 1.45)	4.02e-66	

Exposure	Outcome	Model	Level of adjustment	N	HR, OR or mean difference (95% CI)	p-value	Proportional hazards test (CoxPH)
Social isolation	Depression diagnosis	logistic	adjusted2	119,895	1.23 (1.15 to 1.31)	5.42e-10	
Social isolation	Anxiety diagnosis	logistic	unadjusted	412,222	1.23 (1.18 to 1.28)	5.20e-23	
Social isolation	Anxiety diagnosis	logistic	adjusted1	342,472	1.24 (1.18 to 1.30)	9.87e-20	
Social isolation	Anxiety diagnosis	logistic	adjusted2	119,895	1.07 (0.99 to 1.15)	0.09	
Social isolation	Depression trait	linear	unadjusted	129,379	0.84 (0.75 to 0.93)	8.86e-74	
Social isolation	Depression trait	linear	adjusted1	113,351	0.55 (0.46 to 0.65)	2.06e-30	
Social isolation	Depression trait	linear	adjusted2	78,759	0.33 (0.22 to 0.43)	7.94e-10	
Social isolation	Anxiety trait	linear	unadjusted	129,952	0.35 (0.27 to 0.43)	3.97e-17	
Social isolation	Anxiety trait	linear	adjusted1	113,833	0.15 (0.07 to 0.24)	4.25e-04	
Social isolation	Anxiety trait	linear	adjusted2	79,058	-0.05 (-0.15 to 0.05)	0.32	
Social isolation	Happy	linear	unadjusted	225,839	-0.42 (-0.43 to -0.41)	0.00	
Social isolation	Happy	linear	adjusted1	191,255	-0.37 (-0.38 to -0.35)	0.00	

Exposure	Outcome	Model	Level of adjustment	N	HR, OR or mean difference (95% CI)	p-value	Proportional hazards test (CoxPH)
Social isolation	Happy	linear	adjusted2	93,449	-0.31 (-0.33 to -0.29)	1.88e-212	
Social isolation	Meaning in life	linear	unadjusted	128,700	-0.45 (-0.47 to -0.44)	0.00	
Social isolation	Meaning in life	linear	adjusted1	112,711	-0.40 (-0.42 to -0.38)	1.07e-313	
Social isolation	Meaning in life	linear	adjusted2	78,125	-0.37 (-0.40 to -0.35)	1.58e-189	

HR=hazards ratio, OR=odds ratio, CI=confidence intervals, CoxPH=Cox proportional hazards, CAD=coronary artery disease, HF=heart failure, T2D=type 2 diabetes, SBP=systolic blood pressure, QALYs=quality-adjusted life years

Statistical tests used are indicated in the table and all were two-tailed tests.

Adjusted 1: age when attended assessment centre, sex, assessment centre, Townsend deprivation index, education years based on qualifications, household income before tax and ethnicity

Adjusted 2: adjusted 1 as well as long-standing illness/disability/infirmity and a score for adverse childhood experiences (ACEs)

[a] *For all Cox proportional hazards models the covariate 'centre' was not included due to issues with fitting the model*

[b] *For all Cox proportional hazards models with death as an outcome the covariate ACEs was also not included due to issues with fitting the model*

Table S8. Sibling control analysis results

Exposure	Outcome	Model	Level of adjustment	N	Within-family HR, OR or mean difference (95% CI)	Within-family p-value	Within-family proportional hazards test (CoxPH)	Between-family HR, OR or mean difference (95% CI)	Between-family p-value	Between-family proportional hazards test (CoxPH)
Loneliness	CAD	CoxPH	unadjusted	37,899	1.11 (0.97 to 1.28)	0.13	0.39	1.37 (1.20 to 1.56)	4.55e-06	3.31e-03
Loneliness	CAD	CoxPH	adjusted1[a]	31,845	1.14 (0.98 to 1.33)	0.09	0.60	1.32 (1.13 to 1.53)	3.70e-04	0.12
Loneliness	CAD	CoxPH	adjusted2[a]	11,959	1.27 (0.92 to 1.75)	0.14	0.47	1.30 (0.94 to 1.78)	0.11	0.14
Loneliness	HF	CoxPH	unadjusted	39,736	1.33 (1.08 to 1.63)	6.24e-03	0.36	1.86 (1.53 to 2.26)	3.65e-10	0.07
Loneliness	HF	CoxPH	adjusted1[a]	33,350	1.39 (1.10 to 1.75)	5.56e-03	0.43	1.81 (1.46 to 2.26)	1.09e-07	0.24
Loneliness	HF	CoxPH	adjusted2[a]	32,667	1.26 (1.00 to 1.59)	0.05	0.36	1.58 (1.26 to 1.97)	6.18e-05	0.23
Loneliness	Stroke	CoxPH	unadjusted	39,286	1.22 (0.93 to 1.59)	0.15	0.37	1.30 (1.01 to 1.68)	0.05	0.04
Loneliness	Stroke	CoxPH	adjusted1[a]	32,983	1.32 (0.98 to 1.78)	0.07	0.11	1.41 (1.06 to 1.88)	0.02	8.68e-03
Loneliness	Stroke	CoxPH	adjusted2[a]	32,310	1.28 (0.94 to 1.73)	0.12	0.10	1.27 (0.95 to 1.71)	0.11	0.01
Loneliness	Death	CoxPH	unadjusted	39,950	1.34 (1.17 to 1.53)	2.49e-05	0.02	1.81 (1.59 to 2.05)	1.10e-19	1.02e-03
Loneliness	Death	CoxPH	adjusted1[a,b]	33,515	1.28 (1.10 to 1.50)	1.43e-03	4.54e-03	1.68 (1.45 to 1.95)	2.94e-12	6.72e-03
Loneliness	Death	CoxPH	adjusted2[a,b]	32,828	1.25 (1.07 to 1.46)	5.93e-03	8.11e-03	1.53 (1.32 to 1.78)	2.00e-08	4.87e-03
Loneliness	Multimorbidity	logistic	Unadjusted	39,942	1.93 (1.79 to 2.08)	3.74e-62		2.77 (2.57 to 2.99)	7.44e-155	
Loneliness	Multimorbidity	logistic	adjusted1	33,508	1.88 (1.72 to 2.04)	1.29e-46		2.55 (2.34 to 2.77)	5.69e-103	
Loneliness	Multimorbidity	logistic	adjusted2	12,319	1.73 (1.49 to 2.02)	1.52e-12		2.05 (1.76 to 2.39)	2.93e-20	

Exposure	Outcome	Model	Level of adjustment	N	Within-family HR, OR or mean difference (95% CI)	Within-family p-value	Within-family proportional hazards test (CoxPH)	Between-family HR, OR or mean difference (95% CI)	Between-family p-value	Between-family proportional hazards test (CoxPH)
Loneliness	T2D	logistic	unadjusted	39,950	1.17 (0.99 to 1.39)	0.06		1.80 (1.53 to 2.11)	6.82e-13	
Loneliness	T2D	logistic	adjusted1	33,515	1.27 (1.04 to 1.54)	0.02		1.80 (1.49 to 2.17)	9.73e-10	
Loneliness	T2D	logistic	adjusted2	12,322	0.97 (0.63 to 1.48)	0.87		1.33 (0.87 to 2.04)	0.18	
Loneliness	Hospital admissions	linear	unadjusted	39,950	0.49 (0.14 to 0.84)	6.12e-03		0.90 (0.53 to 1.27)	1.85e-06	
Loneliness	Hospital admissions	linear	adjusted1	33,515	0.42 (0.07 to 0.77)	0.02		0.91 (0.52 to 1.30)	4.10e-06	
Loneliness	Hospital admissions	linear	adjusted2	12,322	0.15 (-0.27 to 0.57)	0.50		0.52 (0.02 to 1.03)	0.04	
Loneliness	SBP	linear	unadjusted	39,922	-2.41 (-3.10 to -1.73)	3.65e-12		-3.31 (-3.96 to -2.66)	1.88e-23	
Loneliness	SBP	linear	adjusted1	33,492	-1.37 (-2.07 to -0.68)	1.13e-04		-1.71 (-2.39 to -1.03)	7.85e-07	
Loneliness	SBP	linear	adjusted2	12,317	-1.43 (-2.59 to -0.27)	0.02		-1.95 (-3.11 to -0.80)	9.40e-04	
Loneliness	QALYs	linear	unadjusted	32,873	-0.03 (-0.04 to -0.02)	1.47e-13		-0.07 (-0.07 to -0.06)	2.69e-55	
Loneliness	QALYs	linear	adjusted1	27,455	-0.02 (-0.03 to -0.01)	3.92e-08		-0.06 (-0.06 to -0.05)	2.75e-38	
Loneliness	QALYs	linear	adjusted2	9,837	-0.01 (-0.02 to -1.37e-03)	0.03		-0.03 (-0.04 to -0.02)	4.12e-08	
Loneliness	Self-harm	logistic	unadjusted	13,113	2.17 (1.61 to 2.92)	3.06e-07		3.49 (2.62 to 4.65)	1.66e-17	
Loneliness	Self-harm	logistic	adjusted1	11,558	1.92 (1.39 to 2.64)	7.18e-05		2.75 (2.01 to 3.77)	2.53e-10	

Exposure	Outcome	Model	Level of adjustment	N	Within-family HR, OR or mean difference (95% CI)	Within-family p-value	Within-family proportional hazards test (CoxPH)	Between-family HR, OR or mean difference (95% CI)	Between-family p-value	Between-family proportional hazards test (CoxPH)
Loneliness	Self-harm	logistic	adjusted2	8,154	1.69 (1.13 to 2.54)	0.01		1.56 (1.04 to 2.33)	0.03	
Loneliness	Suicide attempt	logistic	unadjusted	13,099	2.02 (1.36 to 3.02)	5.44e-04		4.36 (2.97 to 6.41)	6.44e-14	
Loneliness	Suicide attempt	logistic	adjusted1	11,547	1.75 (1.14 to 2.70)	0.01		3.55 (2.34 to 5.38)	2.83e-09	
Loneliness	Suicide attempt	logistic	adjusted2	8,146	1.85 (1.06 to 3.24)	0.03		1.90 (1.10 to 3.31)	0.02	
Loneliness	Depression diagnosis	logistic	unadjusted	39,950	2.66 (2.41 to 2.92)	9.97e-89		3.76 (3.43 to 4.12)	1.41e-174	
Loneliness	Depression diagnosis	logistic	adjusted1	33,515	2.34 (2.11 to 2.61)	4.30e-56		3.27 (2.95 to 3.63)	1.62e-110	
Loneliness	Depression diagnosis	logistic	adjusted2	12,322	2.25 (1.89 to 2.67)	8.17e-20		2.66 (2.23 to 3.17)	4.64e-28	
Loneliness	Anxiety diagnosis	logistic	unadjusted	39,950	1.89 (1.68 to 2.12)	3.54e-27		2.45 (2.20 to 2.74)	2.26e-57	
Loneliness	Anxiety diagnosis	logistic	adjusted1	33,515	1.77 (1.56 to 2.01)	1.15e-18		2.17 (1.92 to 2.46)	1.49e-34	
Loneliness	Anxiety diagnosis	logistic	adjusted2	12,322	1.69 (1.39 to 2.06)	1.93e-07		2.01 (1.65 to 2.46)	5.56e-12	
Loneliness	Depression trait	linear	unadjusted	12,921	2.29 (2.00 to 2.58)	9.24e-53		3.51 (3.22 to 3.80)	9.03e-121	

Exposure	Outcome	Model	Level of adjustment	N	Within-family HR, OR or mean difference (95% CI)	Within-family p-value	Within-family proportional hazards test (CoxPH)	Between-family HR, OR or mean difference (95% CI)	Between-family p-value	Between-family proportional hazards test (CoxPH)
Loneliness	Depression trait	linear	adjusted1	11,400	2.07 (1.76 to 2.38)	1.92e-39		3.12 (2.81 to 3.42)	1.91e-87	
Loneliness	Depression trait	linear	adjusted2	8,077	1.77 (1.41 to 2.12)	1.39e-22		2.56 (2.22 to 2.90)	1.20e-48	
Loneliness	Anxiety trait	linear	unadjusted	12,985	1.59 (1.33 to 1.85)	5.11e-33		2.70 (2.43 to 2.96)	2.22e-86	
Loneliness	Anxiety trait	linear	adjusted1	11,454	1.36 (1.09 to 1.64)	4.64e-22		2.44 (2.15 to 2.72)	1.29e-63	
Loneliness	Anxiety trait	linear	adjusted2	8,100	1.27 (0.94 to 1.59)	2.51e-14		1.95 (1.64 to 2.27)	2.61e-33	
Loneliness	Happy	linear	unadjusted	21,059	-0.51 (-0.55 to -0.47)	1.13e-156		-0.68 (-0.72 to -0.65)	2.51e-285	
Loneliness	Happy	linear	adjusted1	18,077	-0.49 (-0.53 to -0.45)	2.98e-124		-0.63 (-0.67 to -0.59)	2.46e-203	
Loneliness	Happy	linear	adjusted2	9,464	-0.44 (-0.50 to -0.39)	9.56e-52		-0.56 (-0.62 to -0.51)	5.58e-85	
Loneliness	Meaning in life	linear	unadjusted	12,842	-0.41 (-0.47 to -0.35)	5.95e-43		-0.58 (-0.64 to -0.53)	2.78e-87	
Loneliness	Meaning in life	linear	adjusted1	11,341	-0.39 (-0.45 to -0.33)	6.13e-35		-0.55 (-0.61 to -0.49)	1.95e-67	
Loneliness	Meaning in life	linear	adjusted2	8,004	-0.34 (-0.41 to -0.26)	2.67e-19		-0.49 (-0.56 to -0.42)	4.59e-40	
Social isolation	CAD	CoxPH	unadjusted	38,369	1.13 (0.94 to 1.36)	0.21	0.69	1.15 (0.97 to 1.36)	0.11	0.50
Social isolation	CAD	CoxPH	adjusted1[a]	32,355	0.90 (0.73 to 1.10)	0.29	0.95	0.84 (0.70 to 1.01)	0.07	0.42
Social isolation	CAD	CoxPH	adjusted2[a]	12,090	0.75 (0.50 to 1.12)	0.16	0.97	0.85 (0.59 to 1.24)	0.40	0.93

Exposure	Outcome	Model	Level of adjustment	N	Within-family HR, OR or mean difference (95% CI)	Within-family p-value	Within-family proportional hazards test (CoxPH)	Between-family HR, OR or mean difference (95% CI)	Between-family p-value	Between-family proportional hazards test (CoxPH)
Social isolation	HF	CoxPH	unadjusted	40,232	1.29 (0.95 to 1.75)	0.10	0.10	0.86 (0.65 to 1.13)	0.28	0.07
Social isolation	HF	CoxPH	adjusted1[a]	33,888	1.00 (0.72 to 1.39)	1.00	0.04	0.69 (0.51 to 0.94)	0.02	0.16
Social isolation	HF	CoxPH	adjusted2[a]	33,163	1.08 (0.78 to 1.50)	0.65	0.11	0.68 (0.50 to 0.92)	0.01	0.16
Social isolation	Stroke	CoxPH	unadjusted	39,770	0.95 (0.66 to 1.38)	0.79	0.31	0.77 (0.55 to 1.08)	0.12	0.89
Social isolation	Stroke	CoxPH	adjusted1[a]	33,510	0.85 (0.56 to 1.27)	0.42	0.29	0.74 (0.51 to 1.07)	0.11	0.94
Social isolation	Stroke	CoxPH	adjusted2[a]	32,796	0.96 (0.64 to 1.45)	0.85	0.25	0.74 (0.51 to 1.08)	0.11	0.85
Social isolation	Death	CoxPH	unadjusted	40,440	1.71 (1.40 to 2.08)	1.17e-07	0.13	1.07 (0.89 to 1.28)	0.47	0.05
Social isolation	Death	CoxPH	adjusted1[a,b]	34,053	1.57 (1.27 to 1.95)	4.16e-05	0.02	0.96 (0.78 to 1.17)	0.66	0.01
Social isolation	Death	CoxPH	adjusted2[a,b]	33,325	1.67 (1.34 to 2.08)	5.23e-06	0.04	0.94 (0.77 to 1.15)	0.54	0.01
Social isolation	Multimorbidity	logistic	unadjusted	40,431	1.18 (1.08 to 1.30)	3.95e-04		1.72 (1.59 to 1.86)	2.42e-40	
Social isolation	Multimorbidity	logistic	adjusted1	34,046	0.99 (0.90 to 1.10)	0.88		1.14 (1.04 to 1.26)	4.99e-03	

Exposure	Outcome	Model	Level of adjustment	N	Within-family HR, OR or mean difference (95% CI)	Within-family p-value	Within-family proportional hazards test (CoxPH)	Between-family HR, OR or mean difference (95% CI)	Between-family p-value	Between-family proportional hazards test (CoxPH)
Social isolation	Multimorbidity	logistic	adjusted2	12,451	1.05 (0.88 to 1.25)	0.61		1.15 (0.98 to 1.36)	0.09	
Social isolation	T2D	logistic	unadjusted	40,440	1.62 (1.29 to 2.04)	2.91e-05		2.16 (1.77 to 2.63)	4.43e-14	
Social isolation	T2D	logistic	adjusted1	34,053	1.14 (0.88 to 1.48)	0.33		1.00 (0.79 to 1.28)	0.99	
Social isolation	T2D	logistic	adjusted2	12,454	0.94 (0.53 to 1.65)	0.82		0.95 (0.57 to 1.59)	0.85	
Social isolation	Hospital admissions	linear	unadjusted	40,440	0.41 (-0.10 to 0.91)	0.11		1.74 (1.34 to 2.14)	7.86e-18	
Social isolation	Hospital admissions	linear	adjusted1	34,053	-0.23 (-0.74 to 0.28)	0.38		-0.03 (-0.45 to 0.38)	0.87	
Social isolation	Hospital admissions	linear	adjusted2	12,454	-0.26 (-0.75 to 0.24)	0.31		-0.19 (-0.56 to 0.19)	0.33	
Social isolation	SBP	linear	unadjusted	40,410	1.29 (0.44 to 2.14)	3.06e-03		7.48 (6.76 to 8.20)	3.30e-92	
Social isolation	SBP	linear	adjusted1	34,028	-0.25 (-1.12 to 0.62)	0.58		0.23 (-0.55 to 1.00)	0.57	
Social isolation	SBP	linear	adjusted2	12,449	0.69 (-0.65 to 2.03)	0.31		1.02 (-0.21 to 2.24)	0.10	
Social isolation	QALYs	linear	unadjusted	33,251	-0.04 (-0.05 to -0.03)	5.97e-14		-0.08 (-0.09 to -0.07)	6.32e-80	

Exposure	Outcome	Model	Level of adjustment	N	Within-family HR, OR or mean difference (95% CI)	Within-family p-value	Within-family proportional hazards test (CoxPH)	Between-family HR, OR or mean difference (95% CI)	Between-family p-value	Between-family proportional hazards test (CoxPH)
Social isolation	QALYs	linear	adjusted1	27,882	-5.30e-03 (-0.02 to 4.69e-03)	0.30		-5.34e-03 (-0.01 to 3.76e-03)	0.25	
Social isolation	QALYs	linear	adjusted2	9,950	2.10e-03 (-8.64e-03 to 0.01)	0.70		0.01 (1.07e-03 to 0.02)	0.03	
Social isolation	Self-harm	logistic	unadjusted	13,226	2.45 (1.63 to 3.69)	1.85e-05		1.02 (0.71 to 1.46)	0.92	
Social isolation	Self-harm	logistic	adjusted1	11,702	1.77 (1.16 to 2.70)	7.78e-03		1.71 (1.16 to 2.51)	6.71e-03	
Social isolation	Self-harm	logistic	adjusted2	8,228	1.22 (0.73 to 2.02)	0.45		1.37 (0.85 to 2.18)	0.19	
Social isolation	Suicide attempt	logistic	unadjusted	13,213	2.86 (1.61 to 5.06)	3.20e-04		1.39 (0.84 to 2.30)	0.20	
Social isolation	Suicide attempt	logistic	adjusted1	11,692	1.79 (0.99 to 3.23)	0.05		1.83 (1.07 to 3.14)	0.03	
Social isolation	Suicide attempt	logistic	adjusted2	8,221	1.43 (0.68 to 2.98)	0.35		1.42 (0.72 to 2.80)	0.31	
Social isolation	Depression diagnosis	logistic	unadjusted	40,440	1.42 (1.24 to 1.61)	2.01e-07		1.41 (1.26 to 1.58)	3.09e-09	
Social isolation	Depression diagnosis	logistic	adjusted1	34,053	1.28 (1.11 to 1.48)	8.96e-04		1.49 (1.31 to 1.70)	3.35e-09	
Social isolation	Depression diagnosis	logistic	adjusted2	12,454	1.21 (0.97 to 1.52)	0.10		1.36 (1.10 to 1.67)	4.00e-03	

Exposure	Outcome	Model	Level of adjustment	N	Within-family HR, OR or mean difference (95% CI)	Within-family p-value	Within-family proportional hazards test (CoxPH)	Between-family HR, OR or mean difference (95% CI)	Between-family p-value	Between-family proportional hazards test (CoxPH)
Social isolation	Anxiety diagnosis	logistic	unadjusted	40,440	1.30 (1.11 to 1.52)	1.06e-03		1.42 (1.24 to 1.62)	6.14e-07	
Social isolation	Anxiety diagnosis	logistic	adjusted1	34,053	1.30 (1.10 to 1.55)	2.61e-03		1.49 (1.27 to 1.74)	9.41e-07	
Social isolation	Anxiety diagnosis	logistic	adjusted2	12,454	1.21 (0.94 to 1.57)	0.15		1.26 (0.99 to 1.60)	0.06	
Social isolation	Depression trait	linear	unadjusted	13,027	0.44 (0.13 to 0.74)	4.89e-03		-0.07 (-0.34 to 0.21)	0.63	
Social isolation	Depression trait	linear	adjusted1	11,540	0.24 (-0.08 to 0.55)	0.14		0.36 (0.06 to 0.66)	0.02	
Social isolation	Depression trait	linear	adjusted2	8,153	0.24 (-0.10 to 0.59)	0.17		0.13 (-0.19 to 0.45)	0.42	
Social isolation	Anxiety trait	linear	unadjusted	13,092	0.04 (-0.23 to 0.32)	0.76		-0.46 (-0.70 to -0.21)	2.17e-04	
Social isolation	Anxiety trait	linear	adjusted1	11,593	-0.12 (-0.40 to 0.17)	0.43		-0.12 (-0.39 to 0.14)	0.36	
Social isolation	Anxiety trait	linear	adjusted2	8,176	2.55e-03 (-0.31 to 0.31)	0.99		-0.29 (-0.59 to 8.94e-03)	0.06	
Social isolation	Happy	linear	unadjusted	21,282	-0.20 (-0.24 to -0.15)	1.31e-16		-0.16 (-0.20 to -0.12)	6.68e-14	
Social isolation	Happy	linear	adjusted1	18,324	-0.16 (-0.21 to -0.11)	1.37e-10		-0.23 (-0.28 to -0.18)	7.64e-23	

Exposure	Outcome	Model	Level of adjustment	N	Within-family HR, OR or mean difference (95% CI)	Within-family p-value	Within-family proportional hazards test (CoxPH)	Between-family HR, OR or mean difference (95% CI)	Between-family p-value	Between-family proportional hazards test (CoxPH)
Social isolation	Happy	linear	adjusted2	9,561	-0.14 (-0.21 to -0.08)	2.39e-05		-0.20 (-0.26 to -0.14)	7.74e-11	
Social isolation	Meaning in life	linear	unadjusted	12,953	-0.32 (-0.39 to -0.25)	1.25e-20		-0.30 (-0.36 to -0.24)	2.91e-24	
Social isolation	Meaning in life	linear	adjusted1	11,481	-0.30 (-0.37 to -0.23)	3.47e-16		-0.35 (-0.42 to -0.29)	2.18e-26	
Social isolation	Meaning in life	linear	adjusted2	8,075	-0.30 (-0.38 to -0.22)	1.40e-13		-0.33 (-0.40 to -0.25)	1.25e-17	

HR=hazards ratio, OR=odds ratio, CI=confidence intervals, CoxPH=Cox proportional hazards, CAD=coronary artery disease, HF=heart failure, T2D=type 2 diabetes, SBP=systolic blood pressure, QALYs=quality-adjusted life years

Statistical tests used are indicated in the table and all were two-tailed tests.

Adjusted 1: age when attended assessment centre, sex, assessment centre, Townsend deprivation index, education years based on qualifications, household income before tax and ethnicity

Adjusted 2: adjusted 1 as well as long-standing illness/disability/infirmity and a score for adverse childhood experiences (ACEs)

[a] *For all Cox proportional hazards models the covariate 'centre' was not included due to issues with fitting the model*

[b] *For all Cox proportional hazards models with death as an outcome the covariate ACEs was also not included due to issues with fitting the model*

Table S9. One-sample Mendelian Randomisation analysis results from split sample approach

Exposure	Outcome	N	HR, RD or mean difference (MD) (95% CI)	p-value	Meta-analysis heterogeneity test	F statistic
Loneliness	CAD	314,205	HR: 3.65 (0.40 to 33.72)	0.25	1.33; p=0.25	Split sample 1: 26.09; Split sample 2: 54.68
Loneliness	HF	330,211	HR: 18.51 (0.67 to 509.34)	0.08	0.74; p=0.39	Split sample 1: 26.40; Split sample 2: 53.30
Loneliness	Stroke	326,048	HR: 0.18 (3.28e-03 to 9.87)	0.40	1.75e-03; p=0.97	Split sample 1: 25.67; Split sample 2: 54.96
Loneliness	Death	331,995	HR: 1.26 (0.16 to 9.79)	0.83	0.05; p=0.83	Split sample 1: 27.57; Split sample 2: 55.98
Loneliness	Multimorbidity	331,918	RD: 0.71 (0.41 to 1.01)	3.94e-06	2.37; p=0.12	Split sample 1: 28.19; Split sample 2: 55.13
Loneliness	T2D	331,996	RD: 0.22 (0.10 to 0.35)	3.60e-04	2.34; p=0.13	Split sample 1: 28.07; Split sample 2: 55.18
Loneliness	Hospital admissions	331,996	MD: 4.17 (-2.98 to 11.33)	0.25	0.97; p=0.33	Split sample 1: 28.07; Split sample 2: 55.18
Loneliness	SBP	331,696	MD: -0.59 (-10.51 to 9.33)	0.91	0.07; p=0.78	Split sample 1: 27.99; Split sample 2: 54.08
Loneliness	QALYs	269,905	MD: -0.25 (-0.38 to -0.13)	1.00e-04	0.46; p=0.50	Split sample 1: 21.68; Split sample 2: 42.47
Loneliness	Self-harm	108,966	RD: 0.31 (0.08 to 0.54)	7.51e-03	0.06; p=0.81	Split sample 1: 14.65; Split sample 2: 13.13
Loneliness	Suicide attempt	108,827	RD: 0.15 (-7.43e-03 to 0.31)	0.06	1.14; p=0.29	Split sample 1: 14.55; Split sample 2: 13.46

Exposure	Outcome	N	HR, RD or mean difference (MD) (95% CI)	p-value	Meta-analysis heterogeneity test	F statistic
Loneliness	Depression diagnosis	331,996	RD: 0.35 (0.15 to 0.55)	5.18e-04	0.62; p=0.43	Split sample 1: 28.07; Split sample 2: 55.18
Loneliness	Anxiety diagnosis	331,996	RD: 0.13 (-0.04 to 0.29)	0.13	4.77e-03; p=0.94	Split sample 1: 28.07; Split sample 2: 55.18
Loneliness	Depression trait	107,357	MD: 13.00 (7.62 to 18.37)	2.16e-06	6.43e-04; p=0.98	Split sample 1: 12.77; Split sample 2: 13.66
Loneliness	Anxiety trait	107,853	MD: 7.81 (3.88 to 11.74)	9.81e-05	0.11; p=0.73	Split sample 1: 16.07; Split sample 2: 12.49
Loneliness	Happy	178,666	MD: -1.76 (-2.40 to -1.12)	7.98e-08	0.27; p=0.61	Split sample 1: 20.04; Split sample 2: 25.30
Loneliness	Meaning in life	106,828	MD: -0.92 (-1.74 to -0.10)	0.03	5.14e-03; p=0.94	Split sample 1: 14.00; Split sample 2: 15.50
Social isolation	CAD	315,599	HR: 7.43 (0.20 to 277.95)	0.28	2.12; p=0.15	Split sample 1: 32.21; Split sample 2: 9.98
Social isolation	HF	331,588	HR: 0.03 (1.55e-04 to 5.49)	0.19	0.13; p=0.72	Split sample 1: 33.62; Split sample 2: 9.54
Social isolation	Stroke	321,451	HR: 0.53 (9.13e-04 to 302.48)	0.84	0.03; p=0.85	Split sample 1: 32.20; Split sample 2: 10.66
Social isolation	Death	333,357	HR: 9.90 (0.31 to 314.75)	0.19	1.49; p=0.22	Split sample 1: 33.38; Split sample 2: 9.46
Social isolation	Multimorbidity	333,280	RD: 0.02 (-0.41 to 0.45)	0.92	0.11; p=0.74	Split sample 1: 38.74; Split sample 2: 11.30
Social isolation	T2D	333,358	RD: 0.05 (-0.13 to 0.23)	0.58	1.07; p=0.30	Split sample 1: 39.26; Split sample 2: 11.20

Exposure	Outcome	N	HR, RD or mean difference (MD) (95% CI)	p-value	Meta-analysis heterogeneity test	F statistic
Social isolation	Hospital admissions	333,358	MD: -0.25 (-12.18 to 11.67)	0.97	3.66; p=0.06	Split sample 1: 39.26; Split sample 2: 11.20
Social isolation	SBP	333,057	MD: 11.11 (-4.40 to 26.62)	0.16	0.32; p=0.57	Split sample 1: 39.13; Split sample 2: 11.17
Social isolation	QALYs	270,935	MD: -0.05 (-0.24 to 0.14)	0.62	1.11; p=0.29	Split sample 1: 28.16; Split sample 2: 8.69
Social isolation	Self-harm	109,432	RD: 0.14 (-0.08 to 0.37)	0.20	0.16; p=0.69	Split sample 1: 23.20; Split sample 2: 9.02
Social isolation	Suicide attempt	109,291	RD: 0.07 (-0.09 to 0.23)	0.39	1.58; p=0.21	Split sample 1: 23.53; Split sample 2: 9.21
Social isolation	Depression diagnosis	333,358	RD: 0.06 (-0.24 to 0.37)	0.69	0.86; p=0.35	Split sample 1: 39.26; Split sample 2: 11.20
Social isolation	Anxiety diagnosis	333,358	RD: -0.12 (-0.38 to 0.13)	0.35	2.69e-03; p=0.96	Split sample 1: 39.26; Split sample 2: 11.20
Social isolation	Depression trait	107,790	MD: 0.12 (-3.75 to 4.00)	0.95	0.14; p=0.71	Split sample 1: 22.04; Split sample 2: 10.56
Social isolation	Anxiety trait	108,274	MD: -1.29 (-4.95 to 2.37)	0.49	0.27; p=0.60	Split sample 1: 22.74; Split sample 2: 9.46
Social isolation	Happy	179,367	MD: -1.11 (-1.88 to -0.35)	4.42e-03	0.05; p=0.83	Split sample 1: 27.53; Split sample 2: 10.60
Social isolation	Meaning in life	107,258	MD: -1.42 (-2.42 to -0.42)	5.35e-03	0.13; p=0.72	Split sample 1: 19.29; Split sample 2: 10.36

HR=hazards ratio, RD=risk difference, CI=confidence intervals, CAD=coronary artery disease, HF=heart failure, T2D=type 2 diabetes, SBP=systolic blood pressure, QALYs=quality-adjusted life years

One-sample Mendelian randomisation analyses were used and all tests were two-tailed tests.
Adjusted for age, sex, the first 20 principal components (PCs) from PC analysis of genotype data, assessment centre and genotyping chip

Table S10. One-sample Mendelian Randomisation analysis results from unweighted loneliness approach

Exposure	Outcome	N	HR, RD or mean difference (95% CI)	p-value	F statistic
Loneliness	CAD	314,205	1.25 (0.44 to 3.51)	0.68	360.30
Loneliness	HF	330,211	1.81 (0.41 to 7.92)	0.43	385.74
Loneliness	Stroke	326,048	2.40 (0.38 to 15.24)	0.35	377.52
Loneliness	Death	331,995	1.04 (0.40 to 2.71)	0.93	387.44
Loneliness	Multimorbidity	331,918	0.44 (0.31 to 0.57)	2.29e-11	388.26
Loneliness	T2D	331,996	0.05 (-3.68e-04 to 0.10)	0.05	389.09
Loneliness	Hospital admissions	331,996	1.00 (-2.33 to 4.33)	0.56	389.09
Loneliness	SBP	331,696	-10.76 (-15.42 to -6.11)	5.82e-06	387.04
Loneliness	QALYs	269,905	-0.14 (-0.20 to -0.09)	2.47e-07	310.99
Loneliness	Self-harm	108,966	0.23 (0.13 to 0.32)	2.27e-06	147.69
Loneliness	Suicide attempt	108,827	0.16 (0.09 to 0.22)	8.29e-06	147.61
Loneliness	Depression diagnosis	331,996	0.34 (0.25 to 0.43)	3.73e-13	389.09
Loneliness	Anxiety diagnosis	331,996	0.19 (0.11 to 0.26)	1.55e-06	389.09
Loneliness	Depression trait	107,357	7.06 (5.29 to 8.83)	5.72e-15	137.24
Loneliness	Anxiety trait	107,853	4.55 (3.04 to 6.06)	3.30e-09	147.86
Loneliness	Happy	178,666	-1.15 (-1.40 to -0.90)	1.15e-19	235.58
Loneliness	Meaning in life	106,828	-0.97 (-1.34 to -0.60)	2.35e-07	146.14

HR=hazards ratio, RD=risk difference, CI=confidence intervals, CAD=coronary artery disease, HF=heart failure, T2D=type 2 diabetes, SBP=systolic blood pressure, QALYs=quality-adjusted life years
One-sample Mendelian randomisation analyses were used and all tests were two-tailed tests.
Adjusted for age, sex, the first 20 principal components (PCs) from PC analysis of genotype data, assessment centre and genotyping chip

Table S11. One-sample Mendelian Randomisation analysis sensitivity analyses

Exposure	Outcome	Method	HR, OR or mean difference (95% CI)	p-value
Loneliness	CAD	Weighted median	1.09 (0.99 to 1.20)	0.09
Loneliness	CAD	Weighted mode	1.07 (0.86 to 1.34)	0.52
Loneliness	HF	Weighted median	1.05 (0.91 to 1.21)	0.50
Loneliness	HF	Weighted mode	0.91 (0.65 to 1.28)	0.60
Loneliness	Stroke	Weighted median	0.95 (0.80 to 1.12)	0.53
Loneliness	Stroke	Weighted mode	0.89 (0.59 to 1.34)	0.58
Loneliness	Death	Weighted median	1.02 (0.93 to 1.12)	0.65
Loneliness	Death	Weighted mode	1.06 (0.87 to 1.31)	0.56
Loneliness	Multimorbidity	Weighted median	1.06 (1.01 to 1.12)	0.03
Loneliness	Multimorbidity	Weighted mode	1.05 (0.93 to 1.20)	0.41
Loneliness	T2D	Weighted median	1.25 (1.10 to 1.41)	3.73e-04
Loneliness	T2D	Weighted mode	1.30 (0.97 to 1.74)	0.07
Loneliness	Hospital admissions	Weighted median	0.09 (-0.22 to 0.40)	0.56
Loneliness	Hospital admissions	Weighted mode	-0.03 (-0.78 to 0.72)	0.94
Loneliness	SBP	Weighted median	0.01 (-0.43 to 0.45)	0.96
Loneliness	SBP	Weighted mode	-0.26 (-1.23 to 0.71)	0.60
Loneliness	QALYs	Weighted median	-5.78e-03 (-0.01 to -4.10e-04)	0.03
Loneliness	QALYs	Weighted mode	-1.35e-03 (-0.01 to 0.01)	0.84
Loneliness	Self-harm	Weighted median	1.30 (1.04 to 1.62)	0.02
Loneliness	Self-harm	Weighted mode	1.36 (0.77 to 2.41)	0.30
Loneliness	Suicide attempt	Weighted median	1.38 (1.02 to 1.86)	0.04
Loneliness	Suicide attempt	Weighted mode	1.72 (0.80 to 3.73)	0.17

Exposure	Outcome	Method	HR, OR or mean difference (95% CI)	p-value
Loneliness	Depression diagnosis	Weighted median	1.07 (1.00 to 1.15)	0.05
Loneliness	Depression diagnosis	Weighted mode	1.04 (0.87 to 1.24)	0.65
Loneliness	Anxiety diagnosis	Weighted median	1.08 (0.99 to 1.18)	0.07
Loneliness	Anxiety diagnosis	Weighted mode	1.03 (0.83 to 1.26)	0.81
Loneliness	Depression trait	Weighted median	0.34 (0.19 to 0.50)	2.09e-05
Loneliness	Depression trait	Weighted mode	0.55 (0.16 to 0.93)	5.33e-03
Loneliness	Anxiety trait	Weighted median	0.25 (0.11 to 0.39)	3.95e-04
Loneliness	Anxiety trait	Weighted mode	0.25 (-0.07 to 0.58)	0.13
Loneliness	Happy	Weighted median	-0.04 (-0.07 to -0.02)	3.23e-04
Loneliness	Happy	Weighted mode	-0.04 (-0.11 to 0.02)	0.16
Loneliness	Meaning in life	Weighted median	-0.02 (-0.06 to 0.02)	0.34
Loneliness	Meaning in life	Weighted mode	1.85e-03 (-0.09 to 0.09)	0.97
Social isolation	CAD	Weighted median	2.21 (0.99 to 4.95)	0.05
Social isolation	CAD	Weighted mode	3.98 (0.55 to 28.64)	0.17
Social isolation	HF	Weighted median	0.98 (0.32 to 3.04)	0.98
Social isolation	HF	Weighted mode	1.10 (0.09 to 13.14)	0.94
Social isolation	Stroke	Weighted median	1.05 (0.26 to 4.19)	0.94
Social isolation	Stroke	Weighted mode	1.34 (0.05 to 36.94)	0.86
Social isolation	Death	Weighted median	1.26 (0.60 to 2.64)	0.53
Social isolation	Death	Weighted mode	0.48 (0.09 to 2.66)	0.40
Social isolation	Multimorbidity	Weighted median	1.04 (0.68 to 1.58)	0.85

Exposure	Outcome	Method	HR, OR or mean difference (95% CI)	p-value
Social isolation	Multimorbidity	Weighted mode	0.96 (0.37 to 2.51)	0.93
Social isolation	T2D	Weighted median	0.66 (0.24 to 1.83)	0.42
Social isolation	T2D	Weighted mode	0.24 (0.03 to 2.18)	0.20
Social isolation	Hospital admissions	Weighted median	1.08 (-1.44 to 3.59)	0.40
Social isolation	Hospital admissions	Weighted mode	0.09 (-6.11 to 6.30)	0.98
Social isolation	SBP	Weighted median	0.56 (-3.30 to 4.41)	0.78
Social isolation	SBP	Weighted mode	2.13 (-6.05 to 10.30)	0.61
Social isolation	QALYs	Weighted median	-0.01 (-0.06 to 0.03)	0.60
Social isolation	QALYs	Weighted mode	-0.01 (-0.12 to 0.09)	0.78
Social isolation	Self-harm	Weighted median	1.33 (0.22 to 8.03)	0.76
Social isolation	Self-harm	Weighted mode	1.02 (0.02 to 59.08)	0.99
Social isolation	Suicide attempt	Weighted median	2.47 (0.19 to 31.99)	0.49
Social isolation	Suicide attempt	Weighted mode	63.66 (0.16 to 25471.05)	0.17
Social isolation	Depression diagnosis	Weighted median	1.04 (0.57 to 1.88)	0.90
Social isolation	Depression diagnosis	Weighted mode	0.80 (0.18 to 3.69)	0.78
Social isolation	Anxiety diagnosis	Weighted median	0.89 (0.43 to 1.84)	0.76
Social isolation	Anxiety diagnosis	Weighted mode	1.20 (0.26 to 5.57)	0.81
Social isolation	Depression trait	Weighted median	-0.13 (-1.48 to 1.22)	0.85
Social isolation	Depression trait	Weighted mode	-0.99 (-4.19 to 2.21)	0.55

Exposure	Outcome	Method	HR, OR or mean difference (95% CI)	p-value
Social isolation	Anxiety trait	Weighted median	-0.68 (-1.85 to 0.50)	0.26
Social isolation	Anxiety trait	Weighted mode	-0.86 (-3.68 to 1.96)	0.55
Social isolation	Happy	Weighted median	-0.24 (-0.45 to -0.03)	0.02
Social isolation	Happy	Weighted mode	-0.37 (-0.89 to 0.15)	0.16
Social isolation	Meaning in life	Weighted median	-0.15 (-0.46 to 0.15)	0.31
Social isolation	Meaning in life	Weighted mode	0.23 (-0.45 to 0.91)	0.51

HR=hazards ratio, OR=odds ratio, CI=confidence intervals, CAD=coronary artery disease, HF=heart failure, T2D=type 2 diabetes, SBP=systolic blood pressure, QALYs=quality-adjusted life years.
One-sample Mendelian randomisation analyses were used and all tests were two-tailed tests.

Table S12. I-squared values for Two-sample Mendelian Randomisation analyses

Exposure	Outcome	Unweighted I squared	Weighted I squared	Mean F statistic
Loneliness	CAD	0.30	0.00	35.55
Loneliness	Systolic blood pressure	0.30	0.00	35.55
Loneliness	Heart failure	0.30	0.43	35.55
Loneliness	Stroke	0.30	0.00	35.55
Loneliness	T2D	0.30	0.00	35.55
Loneliness	Suicide attempt	0.38	0.00	36.31
Loneliness	Depression	0.30	0.00	35.55
Loneliness	Anxiety	0.30	0.83	35.55
Loneliness	Wellbeing spectrum	0.00	0.00	34.94
Loneliness	Positive affect	0.00	0.00	34.94
Loneliness	Life satisfaction	0.00	0.00	34.94
Social isolation	CAD	0.48	0.00	38.43
Social isolation	Systolic blood pressure	0.51	0.00	38.66
Social isolation	Heart failure	0.48	0.54	38.43
Social isolation	Stroke	0.48	0.24	38.43
Social isolation	T2D	0.48	0.00	38.43
Social isolation	Suicide attempt	0.55	0.00	40.07
Social isolation	Depression	0.48	0.00	38.43
Social isolation	Anxiety	0.48	0.68	38.43
Social isolation	Wellbeing spectrum	0.52	0.00	38.51
Social isolation	Positive affect	0.52	0.00	38.51
Social isolation	Life satisfaction	0.52	0.00	38.51
CAD	Loneliness	0.95	0.94	84.91
Systolic blood pressure	Loneliness	0.66	0.73	44.63
Heart failure	Loneliness	0.73	0.18	42.34
Stroke	Loneliness	0.64	0.58	23.16
T2D	Loneliness	0.96	0.97	103.11

Exposure	Outcome	Unweighted I squared	Weighted I squared	Mean F statistic
Suicide attempt	Loneliness	0.97	1.00	7573.13
Depression	Loneliness	0.20	0.08	11.83
Anxiety	Loneliness	0.38	0.81	21.75
Wellbeing spectrum	Loneliness	0.39	0.02	41.00
Positive affect	Loneliness	0.16	0.36	13.20
Life satisfaction	Loneliness	0.00	0.00	3.49
CAD	Social isolation	0.95	0.94	85.02
Systolic blood pressure	Social isolation	0.65	0.17	44.51
Heart failure	Social isolation	0.73	0.11	42.34
Stroke	Social isolation	0.64	0.59	23.16
T2D	Social isolation	0.96	0.96	102.51
Suicide attempt	Social isolation	0.97	1.00	7573.13
Depression	Social isolation	0.20	0.08	11.83
Anxiety	Social isolation	0.55	0.00	21.84
Wellbeing spectrum	Social isolation	0.39	0.00	41.00
Positive affect	Social isolation	0.15	0.06	13.28
Life satisfaction	Social isolation	0.00	0.00	3.49

CAD=coronary artery disease, HF=heart failure, T2D=type 2 diabetes, SBP=systolic blood pressure, QALYs=quality-adjusted life years

Table S13. Two-sample Mendelian Randomisation analysis results with loneliness and social isolation as exposures

Exposure	Outcome	Method	N genetic variants	OR or mean difference (95% CI)	p-value	Heterogeneity test p-value	Directional pleiotropy intercept (95% CI; p-value)	Additional tests for MR approaches	Steiger filtering - percentage of genetic variants
Loneliness	CAD	Inverse variance weighted	16	1.15 (0.92 to 1.44)	0.22	4.68e-04			100.00
Loneliness	CAD	MR-Egger	16	2.14 (0.67 to 6.86)	0.22	7.58e-04	-0.01 (-0.04 to 0.01, 0.31)		
Loneliness	CAD	SIMEX adjusted MR-Egger	16	1.75 (0.58 to 5.23)	0.33		-8.47e-03 (-0.03 to 0.02, 0.51)		
Loneliness	CAD	Weighted median	16	1.19 (0.95 to 1.49)	0.14				
Loneliness	CAD	MR-RAPS	16	1.21 (0.96 to 1.51)	0.10				
Loneliness	CAD	PRESSO - raw	16	1.15 (0.92 to 1.44)	0.24			Global = 45.23; p=1.50e-03, Distortion = NA; p=NA	
Loneliness	CAD	PRESSO - outlier corrected	15 (1 removed)	NA (NA to NA)					
Loneliness	CAD	MRLAP - observed	14	1.04 (0.94 to 1.15)	0.43				

Exposure	Outcome	Method	N genetic variants	OR or mean difference (95% CI)	p-value	Heterogeneity test p-value	Directional pleiotropy intercept (95% CI; p-value)	Additional tests for MR approaches	Steiger filtering - percentage of genetic variants
Loneliness	CAD	MRLAP - corrected	14	1.05 (0.90 to 1.23)	0.52			Corrected vs observed estimate difference test = -0.41; p=0.68	
Loneliness	CAD	MR-CAUSE[a]		1.23 (1.07 to 1.42)				Causal vs sharing model ELPD: delta (SE) = -3.12 (1.90); p=0.05	
Loneliness	Systolic blood pressure	Inverse variance weighted	16	-2.63 (-5.90 to 0.63)	0.11	2.00e-40			87.50
Loneliness	Systolic blood pressure	MR-Egger	16	-6.72 (-24.10 to 10.67)	0.46	2.60e-40	0.09 (-0.27 to 0.44, 0.65)		
Loneliness	Systolic blood pressure	SIMEX adjusted MR-Egger	16	-2.66 (-19.64 to 14.33)	0.76		-7.27e-04 (-0.38 to 0.38, 1.00)		
Loneliness	Systolic blood pressure	Weighted median	16	-2.07 (-3.80 to -0.34)	0.02				
Loneliness	Systolic blood pressure	MR-RAPS	16	-3.03 (-6.26 to 0.19)	0.07				

Exposure	Outcome	Method	N genetic variants	OR or mean difference (95% CI)	p-value	Heterogeneity test p-value	Directional pleiotropy intercept (95% CI; p-value)	Additional tests for MR approaches	Steiger filtering - percentage of genetic variants
Loneliness	Systolic blood pressure	PRESSO - raw	16	-2.63 (-5.90 to 0.63)	0.13			Global = 259.59; p=<5e-04, Distortion = -45.25; p=<5e-04	
Loneliness	Systolic blood pressure	PRESSO - outlier corrected	9 (7 removed)	-1.81 (-3.62 to -6.28e-03)	0.08				
Loneliness	Systolic blood pressure	MRLAP - observed	8	-0.27 (-0.71 to 0.16)	0.22				
Loneliness	Systolic blood pressure	MRLAP - corrected	8	-0.44 (-1.17 to 0.28)	0.23			Corrected vs observed estimate difference test = 1.18; p=0.24	
Loneliness	Systolic blood pressure	MR-CAUSE[a]		-0.46 (-1.78 to 0.86)				Causal vs sharing model ELPD: delta (SE) = 0.49 (0.48); p=0.85	
Loneliness	Heart failure	Inverse variance weighted	16	0.95 (0.73 to 1.24)	0.72	0.13			100.00
Loneliness	Heart failure	MR-Egger	16	1.51 (0.37 to 6.12)	0.57	0.11	-9.67e-03 (-0.04 to 0.02, 0.52)		

Exposure	Outcome	Method	N genetic variants	OR or mean difference (95% CI)	p-value	Heterogeneity test p-value	Directional pleiotropy intercept (95% CI; p-value)	Additional tests for MR approaches	Steiger filtering - percentage of genetic variants
Loneliness	Heart failure	SIMEX adjusted MR-Egger	16	1.95 (0.26 to 14.44)	0.52		-0.01 (-0.06 to 0.03, 0.49)		
Loneliness	Heart failure	Weighted median	16	1.02 (0.75 to 1.39)	0.89				
Loneliness	Heart failure	MR-RAPS	16	1.01 (0.79 to 1.29)	0.95				
Loneliness	Heart failure	PRESSO - raw	16	0.95 (0.73 to 1.24)	0.72			Global = 24.05; p=0.14, Distortion = NA; p=NA	
Loneliness	Heart failure	PRESSO - outlier corrected	16 (0 removed)	NA (NA to NA)					
Loneliness	Heart failure	MRLAP - observed	15	0.98 (0.90 to 1.07)	0.70				
Loneliness	Heart failure	MRLAP - corrected	15	0.97 (0.85 to 1.10)	0.61			Corrected vs observed estimate difference test = 0.74; p=0.46	
Loneliness	Heart failure	MR-CAUSE[a]		1.12 (0.96 to 1.30)				Causal vs sharing model ELPD: delta (SE) = -0.40 (1.25); p=0.38	

Exposure	Outcome	Method	N genetic variants	OR or mean difference (95% CI)	p-value	Heterogeneity test p-value	Directional pleiotropy intercept (95% CI; p-value)	Additional tests for MR approaches	Steiger filtering - percentage of genetic variants
Loneliness	Stroke	Inverse variance weighted	16	0.90 (0.61 to 1.31)	0.58	9.25e-03			50.00
Loneliness	Stroke	MR-Egger	16	1.54 (0.20 to 12.10)	0.69	7.06e-03	-0.01 (-0.05 to 0.03, 0.61)		
Loneliness	Stroke	SIMEX adjusted MR-Egger	16	0.77 (0.11 to 5.19)	0.79		3.66e-03 (-0.04 to 0.05, 0.87)		
Loneliness	Stroke	Weighted median	16	1.09 (0.73 to 1.61)	0.68				
Loneliness	Stroke	MR-RAPS	16	0.95 (0.66 to 1.36)	0.78				
Loneliness	Stroke	PRESSO - raw	16	0.90 (0.61 to 1.31)	0.58			Global = 35.03; p=0.01, Distortion = -1049.68; p=0.04	
Loneliness	Stroke	PRESSO - outlier corrected	15 (1 removed)	1.01 (0.74 to 1.38)	0.94				
Loneliness	Stroke	MRLAP - observed	8	0.92 (0.83 to 1.04)	0.18				
Loneliness	Stroke	MRLAP - corrected	8	0.88 (0.73 to 1.06)	0.17			Corrected vs observed estimate difference test = 1.39; p=0.16	

Exposure	Outcome	Method	N genetic variants	OR or mean difference (95% CI)	p-value	Heterogeneity test p-value	Directional pleiotropy intercept (95% CI; p-value)	Additional tests for MR approaches	Steiger filtering - percentage of genetic variants
Loneliness	Stroke	MR-CAUSE[a]		1.09 (0.88 to 1.38)				Causal vs sharing model ELPD: delta (SE) = 0.62 (0.68); p=0.82	
Loneliness	T2D	Inverse variance weighted	16	1.19 (0.85 to 1.67)	0.31	2.60e-14			100.00
Loneliness	T2D	MR-Egger	16	2.83 (0.47 to 17.18)	0.28	1.38e-13	-0.02 (-0.06 to 0.02, 0.35)		
Loneliness	T2D	SIMEX adjusted MR-Egger	16	2.68 (0.43 to 16.78)	0.31		-0.02 (-0.06 to 0.02, 0.45)		
Loneliness	T2D	Weighted median	16	1.00 (0.80 to 1.25)	0.98				
Loneliness	T2D	MR-RAPS	16	1.23 (0.89 to 1.71)	0.21				
Loneliness	T2D	PRESSO - raw	16	1.19 (0.85 to 1.67)	0.33			Global = 111.57; p=<5e-04, Distortion = 76.99; p=0.15	
Loneliness	T2D	PRESSO - outlier corrected	13 (3 removed)	1.10 (0.90 to 1.36)	0.37				

Exposure	Outcome	Method	N genetic variants	OR or mean difference (95% CI)	p-value	Heterogeneity test p-value	Directional pleiotropy intercept (95% CI; p-value)	Additional tests for MR approaches	Steiger filtering - percentage of genetic variants
Loneliness	T2D	MRLAP - observed	14	1.16 (1.00 to 1.34)	0.05				
Loneliness	T2D	MRLAP - corrected	14	1.23 (0.99 to 1.54)	0.06			Corrected vs observed estimate difference test = -1.69; p=0.09	
Loneliness	T2D	MR-CAUSE[a]		1.26 (1.04 to 1.51)				Causal vs sharing model ELPD: delta (SE) = -2.12 (1.70); p=0.11	
Loneliness	Suicide attempt	Inverse variance weighted	14	2.42 (1.46 to 3.99)	5.57e-04	2.58e-04			100.00
Loneliness	Suicide attempt	MR-Egger	14	7.10 (0.52 to 97.31)	0.17	2.94e-04	-0.02 (-0.08 to 0.03, 0.43)		
Loneliness	Suicide attempt	SIMEX adjusted MR-Egger	14	4.18 (0.29 to 61.01)	0.32		-0.01 (-0.07 to 0.05, 0.72)		
Loneliness	Suicide attempt	Weighted median	14	1.76 (1.05 to 2.93)	0.03				
Loneliness	Suicide attempt	MR-RAPS	14	2.60 (1.52 to 4.45)	4.84e-04				

Exposure	Outcome	Method	N genetic variants	OR or mean difference (95% CI)	p-value	Heterogeneity test p-value	Directional pleiotropy intercept (95% CI; p-value)	Additional tests for MR approaches	Steiger filtering - percentage of genetic variants
Loneliness	Suicide attempt	PRESSO - raw	14	2.42 (1.46 to 3.99)	4.30e-03			Global = 44.86; p=<5e-04, Distortion = NA; p=NA	
Loneliness	Suicide attempt	PRESSO - outlier corrected	13 (1 removed)	NA (NA to NA)					
Loneliness	Suicide attempt	MRLAP - observed	13	1.30 (1.11 to 1.53)	1.41e-03				
Loneliness	Suicide attempt	MRLAP - corrected	13	1.48 (1.15 to 1.90)	2.25e-03			Corrected vs observed estimate difference test = -2.99; p=2.82e-03	
Loneliness	Suicide attempt	MR-CAUSE[a]		1.72 (1.28 to 2.29)				Causal vs sharing model ELPD: delta (SE) = -1.87 (1.81); p=0.15	
Loneliness	Depression	Inverse variance weighted	16	2.13 (1.42 to 3.20)	2.74e-04	4.77e-04			87.50
Loneliness	Depression	MR-Egger	16	0.52 (0.07 to 4.11)	0.55	1.37e-03	0.03 (-0.01 to 0.07, 0.20)		

Exposure	Outcome	Method	N genetic variants	OR or mean difference (95% CI)	p-value	Heterogeneity test p-value	Directional pleiotropy intercept (95% CI; p-value)	Additional tests for MR approaches	Steiger filtering - percentage of genetic variants
Loneliness	Depression	SIMEX adjusted MR-Egger	16	0.14 (0.02 to 0.99)	0.07		0.06 (0.01 to 0.10, 0.02)		
Loneliness	Depression	Weighted median	16	2.08 (1.40 to 3.09)	3.06e-04				
Loneliness	Depression	MR-RAPS	16	1.95 (1.20 to 3.18)	7.33e-03				
Loneliness	Depression	PRESSO - raw	16	2.13 (1.42 to 3.20)	2.43e-03			Global = 45.25; p=2.00e-03, Distortion = 20.86; p=0.30	
Loneliness	Depression	PRESSO - outlier corrected	15 (1 removed)	1.87 (1.33 to 2.62)	2.71e-03				
Loneliness	Depression	MRLAP - observed	14	1.61 (1.21 to 2.15)	1.23e-03				
Loneliness	Depression	MRLAP - corrected	14	2.07 (1.33 to 3.21)	1.25e-03			Corrected vs observed estimate difference test = -3.30; p=9.81e-04	
Loneliness	Depression	MR-CAUSE[a]		1.72 (1.39 to 2.12)				Causal vs sharing model ELPD: delta (SE) = -5.37 (1.83); p=1.63e-03	

Exposure	Outcome	Method	N genetic variants	OR or mean difference (95% CI)	p-value	Heterogeneity test p-value	Directional pleiotropy intercept (95% CI; p-value)	Additional tests for MR approaches	Steiger filtering - percentage of genetic variants
Loneliness	Anxiety	Inverse variance weighted	16	1.91 (0.84 to 4.36)	0.12	0.38			43.75
Loneliness	Anxiety	MR-Egger	16	0.50 (5.26e-03 to 47.21)	0.77	0.33	0.03 (-0.06 to 0.12, 0.56)		
Loneliness	Anxiety	SIMEX adjusted MR-Egger	16	0.34 (4.44e-04 to 260.60)	0.75		0.04 (-0.10 to 0.17, 0.61)		
Loneliness	Anxiety	Weighted median	16	0.99 (0.33 to 2.99)	0.98				
Loneliness	Anxiety	MR-RAPS	16	1.88 (0.79 to 4.46)	0.15				
Loneliness	Anxiety	PRESSO - raw	16	1.91 (0.84 to 4.36)	0.14			Global = 18.30; p=0.39, Distortion = NA; p=NA	
Loneliness	Anxiety	PRESSO - outlier corrected	16 (0 removed)	NA (NA to NA)					
Loneliness	Anxiety	MRLAP - observed	15	1.44 (0.85 to 2.41)	0.17				
Loneliness	Anxiety	MRLAP - corrected	15	1.76 (0.80 to 3.86)	0.16			Corrected vs observed estimate difference test = -1.52; p=0.13	

Exposure	Outcome	Method	N genetic variants	OR or mean difference (95% CI)	p-value	Heterogeneity test p-value	Directional pleiotropy intercept (95% CI; p-value)	Additional tests for MR approaches	Steiger filtering - percentage of genetic variants
Loneliness	Anxiety	MR-CAUSE[a]		1.55 (0.91 to 2.51)				Causal vs sharing model ELPD: delta (SE) = -0.70 (1.27); p=0.29	
Loneliness	Wellbeing spectrum	Inverse variance weighted	12	-0.28 (-0.32 to -0.23)	4.55e-33	6.50e-03			100.00
Loneliness	Wellbeing spectrum	MR-Egger	12	-0.25 (-0.56 to 0.06)	0.14	3.85e-03	-5.19e-04 (-6.62e-03 to 5.58e-03, 0.87)		
Loneliness	Wellbeing spectrum	SIMEX adjusted MR-Egger	12	-0.46 (-0.93 to 8.80e-03)	0.08		3.52e-03 (-5.65e-03 to 0.01, 0.47)		
Loneliness	Wellbeing spectrum	Weighted median	12	-0.25 (-0.30 to -0.19)	1.68e-17				
Loneliness	Wellbeing spectrum	MR-RAPS	12	-0.29 (-0.33 to -0.24)	0.00				
Loneliness	Wellbeing spectrum	PRESSO - raw	12	-0.28 (-0.32 to -0.23)	1.18e-07			Global = 30.85; p=0.22, Distortion = NA; p=NA	
Loneliness	Wellbeing spectrum	PRESSO - outlier corrected	12 (0 removed)	NA (NA to NA)					

Exposure	Outcome	Method	N genetic variants	OR or mean difference (95% CI)	p-value	Heterogeneity test p-value	Directional pleiotropy intercept (95% CI; p-value)	Additional tests for MR approaches	Steiger filtering - percentage of genetic variants
Loneliness	Wellbeing spectrum	MRLAP - observed	9	-0.42 (-0.51 to -0.33)	4.26e-21				
Loneliness	Wellbeing spectrum	MRLAP - corrected	9	-0.57 (-0.72 to -0.43)	1.58e-15			Corrected vs observed estimate difference test = 5.40; p=6.71e-08	
Loneliness	Wellbeing spectrum	MR-CAUSE[a]		-0.23 (-0.27 to -0.19)				Causal vs sharing model ELPD: delta (SE) = -7.87 (1.29); p=5.23e-10	
Loneliness	Positive affect	Inverse variance weighted	12	-0.35 (-0.46 to -0.24)	3.37e-10	6.02e-03			91.67
Loneliness	Positive affect	MR-Egger	12	-0.34 (-1.08 to 0.39)	0.38	3.45e-03	-6.41e-05 (-0.01 to 0.01, 0.99)		
Loneliness	Positive affect	SIMEX adjusted MR-Egger	12	-0.62 (-1.75 to 0.51)	0.31		5.25e-03 (-0.02 to 0.03, 0.65)		
Loneliness	Positive affect	Weighted median	12	-0.30 (-0.40 to -0.20)	3.89e-09				
Loneliness	Positive affect	MR-RAPS	12	-0.31 (-0.39 to -0.23)	7.77e-15				

Exposure	Outcome	Method	N genetic variants	OR or mean difference (95% CI)	p-value	Heterogeneity test p-value	Directional pleiotropy intercept (95% CI; p-value)	Additional tests for MR approaches	Steiger filtering - percentage of genetic variants
Loneliness	Positive affect	PRESSO - raw	12	-0.35 (-0.46 to -0.24)	6.00e-05			Global = 30.81; p=0.03, Distortion = -15.38; p=0.04	
Loneliness	Positive affect	PRESSO - outlier corrected	11 (1 removed)	-0.30 (-0.36 to -0.25)	9.17e-07				
Loneliness	Positive affect	MRLAP - observed	9	-0.61 (-0.81 to -0.41)	3.43e-09				
Loneliness	Positive affect	MRLAP - corrected	9	-0.87 (-1.20 to -0.55)	1.15e-07			Corrected vs observed estimate difference test = 3.97; p=7.26e-05	
Loneliness	Positive affect	MR-CAUSE[a]		-0.23 (-0.29 to -0.16)				Causal vs sharing model ELPD: delta (SE) = -5.89 (1.78); p=4.85e-04	
Loneliness	Life satisfaction	Inverse variance weighted	12	-0.47 (-0.69 to -0.24)	4.28e-05	0.02			58.33
Loneliness	Life satisfaction	MR-Egger	12	0.28 (-1.17 to 1.73)	0.71	0.03	-0.01 (-0.04 to 0.01, 0.33)		
Loneliness	Life satisfaction	SIMEX adjusted MR-Egger	12	0.51 (-1.72 to 2.75)	0.66		-0.02 (-0.06 to 0.02, 0.40)		

Exposure	Outcome	Method	N genetic variants	OR or mean difference (95% CI)	p-value	Heterogeneity test p-value	Directional pleiotropy intercept (95% CI; p-value)	Additional tests for MR approaches	Steiger filtering - percentage of genetic variants
Loneliness	Life satisfaction	Weighted median	12	-0.29 (-0.52 to -0.05)	0.02				
Loneliness	Life satisfaction	MR-RAPS	12	-0.46 (-0.70 to -0.22)	1.61e-04				
Loneliness	Life satisfaction	PRESSO - raw	12	-0.47 (-0.69 to -0.24)	1.78e-03			Global = 26.48; p=0.04, Distortion = -16.63; p=0.37	
Loneliness	Life satisfaction	PRESSO - outlier corrected	11 (1 removed)	-0.40 (-0.60 to -0.21)	2.28e-03				
Loneliness	Life satisfaction	MRLAP - observed	9	-0.77 (-1.16 to -0.38)	1.14e-04				
Loneliness	Life satisfaction	MRLAP - corrected	9	-1.26 (-1.88 to -0.63)	8.06e-05			Corrected vs observed estimate difference test = 3.79; p=1.49e-04	
Loneliness	Life satisfaction	MR-CAUSE[a]		-0.22 (-0.34 to -0.08)				Causal vs sharing model ELPD: delta (SE) = -1.58 (1.79); p=0.19	
Social isolation	CAD	Inverse variance weighted	15	0.66 (0.25 to 1.79)	0.42	7.01e-04			100.00

Exposure	Outcome	Method	N genetic variants	OR or mean difference (95% CI)	p-value	Heterogeneity test p-value	Directional pleiotropy intercept (95% CI; p-value)	Additional tests for MR approaches	Steiger filtering - percentage of genetic variants
Social isolation	CAD	MR-Egger	15	0.03 (3.64e-04 to 2.93)	0.16	1.95e-03	0.01 (-6.50e-03 to 0.03, 0.20)		
Social isolation	CAD	SIMEX adjusted MR-Egger	15	0.01 (2.26e-04 to 0.79)	0.06		0.02 (-1.07e-03 to 0.04, 0.09)		
Social isolation	CAD	Weighted median	15	0.42 (0.17 to 1.06)	0.07				
Social isolation	CAD	MR-RAPS	15	0.57 (0.20 to 1.58)	0.28				
Social isolation	CAD	PRESSO - raw	15	0.66 (0.25 to 1.79)	0.43			Global = 42.92; p=<5e-04, Distortion = -13.91; p=0.87	
Social isolation	CAD	PRESSO - outlier corrected	13 (2 removed)	0.70 (0.29 to 1.68)	0.44				
Social isolation	CAD	MRLAP - observed	16	0.96 (0.88 to 1.04)	0.30				
Social isolation	CAD	MRLAP - corrected	16	0.94 (0.84 to 1.05)	0.29			Corrected vs observed estimate difference test = 1.00; p=0.32	

Exposure	Outcome	Method	N genetic variants	OR or mean difference (95% CI)	p-value	Heterogeneity test p-value	Directional pleiotropy intercept (95% CI; p-value)	Additional tests for MR approaches	Steiger filtering - percentage of genetic variants
Social isolation	CAD	MR-CAUSE[a]		1.00 (0.53 to 1.84)				Causal vs sharing model ELPD: delta (SE) = 0.89 (0.07); p=1.00	
Social isolation	Systolic blood pressure	Inverse variance weighted	14	1.80 (-10.71 to 14.31)	0.78	2.70e-23			100.00
Social isolation	Systolic blood pressure	MR-Egger	14	-30.28 (-85.70 to 25.14)	0.31	5.36e-21	0.15 (-0.11 to 0.41, 0.27)		
Social isolation	Systolic blood pressure	SIMEX adjusted MR-Egger	14	-50.99 (-102.65 to 0.68)	0.08		0.25 (-0.01 to 0.51, 0.09)		
Social isolation	Systolic blood pressure	Weighted median	14	1.44 (-5.73 to 8.62)	0.69				
Social isolation	Systolic blood pressure	MR-RAPS	14	-2.64 (-16.77 to 11.49)	0.71				
Social isolation	Systolic blood pressure	PRESSO - raw	14	1.80 (-10.71 to 14.31)	0.78			Global = 166.46; p=<5e-04, Distortion = -71.24; p=0.64	

Exposure	Outcome	Method	N genetic variants	OR or mean difference (95% CI)	p-value	Heterogeneity test p-value	Directional pleiotropy intercept (95% CI; p-value)	Additional tests for MR approaches	Steiger filtering - percentage of genetic variants
Social isolation	Systolic blood pressure	PRESSO - outlier corrected	10 (4 removed)	6.26 (-0.96 to 13.48)	0.12				
Social isolation	Systolic blood pressure	MRLAP - observed	15	-5.76e-04 (-0.22 to 0.21)	1.00				
Social isolation	Systolic blood pressure	MRLAP - corrected	15	3.81e-04 (-0.31 to 0.31)	1.00			Corrected vs observed estimate difference test = -0.02; p=0.98	
Social isolation	Systolic blood pressure	MR-CAUSE[a]		2.13 (-3.66 to 7.91)				Causal vs sharing model ELPD: delta (SE) = 0.41 (0.55); p=0.77	
Social isolation	Heart failure	Inverse variance weighted	15	0.63 (0.18 to 2.22)	0.48	0.07			100.00
Social isolation	Heart failure	MR-Egger	15	0.16 (4.28e-04 to 58.58)	0.55	0.06	6.67e-03 (-0.02 to 0.03, 0.65)		
Social isolation	Heart failure	SIMEX adjusted MR-Egger	15	0.07 (2.59e-05 to 204.68)	0.53		0.01 (-0.03 to 0.05, 0.60)		
Social isolation	Heart failure	Weighted median	15	0.70 (0.18 to 2.75)	0.61				

Exposure	Outcome	Method	N genetic variants	OR or mean difference (95% CI)	p-value	Heterogeneity test p-value	Directional pleiotropy intercept (95% CI; p-value)	Additional tests for MR approaches	Steiger filtering - percentage of genetic variants
Social isolation	Heart failure	MR-RAPS	15	0.66 (0.19 to 2.28)	0.51				
Social isolation	Heart failure	PRESSO - raw	15	0.63 (0.18 to 2.22)	0.49			Global = 25.28; p=0.08, Distortion = NA; p=NA	
Social isolation	Heart failure	PRESSO - outlier corrected	15 (0 removed)	NA (NA to NA)					
Social isolation	Heart failure	MRLAP - observed	15	0.98 (0.91 to 1.05)	0.52				
Social isolation	Heart failure	MRLAP - corrected	15	0.96 (0.86 to 1.07)	0.48			Corrected vs observed estimate difference test = 0.81; p=0.42	
Social isolation	Heart failure	MR-CAUSE[a]		0.88 (0.41 to 1.88)				Causal vs sharing model ELPD: delta (SE) = 0.76 (0.29); p=1.00	
Social isolation	Stroke	Inverse variance weighted	15	1.32 (0.42 to 4.15)	0.64	0.92			50.00
Social isolation	Stroke	MR-Egger	15	4.25 (0.02 to 786.29)	0.60	0.89	-5.57e-03 (-0.03 to 0.02, 0.66)		

Exposure	Outcome	Method	N genetic variants	OR or mean difference (95% CI)	p-value	Heterogeneity test p-value	Directional pleiotropy intercept (95% CI; p-value)	Additional tests for MR approaches	Steiger filtering - percentage of genetic variants
Social isolation	Stroke	SIMEX adjusted MR-Egger	15	11.02 (0.28 to 431.28)	0.22		-9.82e-03 (-0.03 to 8.38e-03, 0.31)		
Social isolation	Stroke	Weighted median	15	0.76 (0.17 to 3.39)	0.71				
Social isolation	Stroke	MR-RAPS	15	1.29 (0.38 to 4.35)	0.68				
Social isolation	Stroke	PRESSO - raw	15	1.32 (0.57 to 3.04)	0.53			Global = 8.73; p=0.92, Distortion = NA; p=NA	
Social isolation	Stroke	PRESSO - outlier corrected	15 (0 removed)	NA (NA to NA)					
Social isolation	Stroke	MRLAP - observed	15	1.01 (0.96 to 1.06)	0.63				
Social isolation	Stroke	MRLAP - corrected	15	1.02 (0.95 to 1.09)	0.67			Corrected vs observed estimate difference test = -0.31; p=0.76	
Social isolation	Stroke	MR-CAUSE[a]		1.07 (0.48 to 2.39)				Causal vs sharing model ELPD: delta (SE) = 0.88 (0.11); p=1.00	

Exposure	Outcome	Method	N genetic variants	OR or mean difference (95% CI)	p-value	Heterogeneity test p-value	Directional pleiotropy intercept (95% CI; p-value)	Additional tests for MR approaches	Steiger filtering - percentage of genetic variants
Social isolation	T2D	Inverse variance weighted	15	0.95 (0.14 to 6.31)	0.96	3.66e-23			100.00
Social isolation	T2D	MR-Egger	15	1.68 (1.86e-04 to 15287.64)	0.91	1.17e-23	-2.67e-03 (-0.04 to 0.04, 0.90)		
Social isolation	T2D	SIMEX adjusted MR-Egger	15	0.04 (1.24e-05 to 135.00)	0.45		0.01 (-0.03 to 0.05, 0.50)		
Social isolation	T2D	Weighted median	15	1.30 (0.48 to 3.47)	0.61				
Social isolation	T2D	MR-RAPS	15	0.76 (0.14 to 4.23)	0.75				
Social isolation	T2D	PRESSO - raw	15	0.95 (0.14 to 6.31)	0.96			Global = 160.73; p=<5e-04, Distortion = -254.40; p=0.04	
Social isolation	T2D	PRESSO - outlier corrected	13 (2 removed)	1.03 (0.36 to 2.96)	0.95				
Social isolation	T2D	MRLAP - observed	16	0.99 (0.84 to 1.17)	0.90				
Social isolation	T2D	MRLAP - corrected	16	0.98 (0.77 to 1.24)	0.88			Corrected vs observed estimate	

Exposure	Outcome	Method	N genetic variants	OR or mean difference (95% CI)	p-value	Heterogeneity test p-value	Directional pleiotropy intercept (95% CI; p-value)	Additional tests for MR approaches	Steiger filtering - percentage of genetic variants
								difference test = 0.20; p=0.84	
Social isolation	T2D	MR-CAUSE[a]		0.87 (0.41 to 1.82)				Causal vs sharing model ELPD: delta (SE) = 0.70 (0.31); p=0.99	
Social isolation	Suicide attempt	Inverse variance weighted	12	4.85 (0.26 to 89.71)	0.29	1.16e-07			100.00
Social isolation	Suicide attempt	MR-Egger	12	3.51e-03 (8.10e-09 to 1519.03)	0.41	6.17e-07	0.04 (-0.03 to 0.10, 0.29)		
Social isolation	Suicide attempt	SIMEX adjusted MR-Egger	12	0.27 (2.13e-06 to 34280.17)	0.83		0.02 (-0.05 to 0.08, 0.63)		
Social isolation	Suicide attempt	Weighted median	12	1.22 (0.16 to 8.98)	0.85				
Social isolation	Suicide attempt	MR-RAPS	12	2.90 (0.32 to 26.58)	0.35				
Social isolation	Suicide attempt	PRESSO - raw	12	4.85 (0.26 to 89.71)	0.31			Global = 65.25; p=<5e-04, Distortion = 16.17; p=0.72	

Exposure	Outcome	Method	N genetic variants	OR or mean difference (95% CI)	p-value	Heterogeneity test p-value	Directional pleiotropy intercept (95% CI; p-value)	Additional tests for MR approaches	Steiger filtering - percentage of genetic variants
Social isolation	Suicide attempt	PRESSO - outlier corrected	10 (2 removed)	3.89 (0.72 to 21.02)	0.15				
Social isolation	Suicide attempt	MRLAP - observed	15	1.11 (0.94 to 1.31)	0.23				
Social isolation	Suicide attempt	MRLAP - corrected	15	1.15 (0.90 to 1.47)	0.26			Corrected vs observed estimate difference test = -0.99; p=0.32	
Social isolation	Suicide attempt	MR-CAUSE[a]		2.94 (1.02 to 8.33)				Causal vs sharing model ELPD: delta (SE) = -1.27 (1.55); p=0.21	
Social isolation	Depression	Inverse variance weighted	15	1.42 (0.22 to 9.21)	0.72	1.91e-04			100.00
Social isolation	Depression	MR-Egger	15	0.12 (1.71e-05 to 826.61)	0.65	1.47e-04	0.01 (-0.03 to 0.05, 0.58)		
Social isolation	Depression	SIMEX adjusted MR-Egger	15	0.30 (3.58e-05 to 2501.49)	0.80		8.01e-03 (-0.04 to 0.05, 0.73)		
Social isolation	Depression	Weighted median	15	2.18 (0.44 to 10.88)	0.34				

Exposure	Outcome	Method	N genetic variants	OR or mean difference (95% CI)	p-value	Heterogeneity test p-value	Directional pleiotropy intercept (95% CI; p-value)	Additional tests for MR approaches	Steiger filtering - percentage of genetic variants
Social isolation	Depression	MR-RAPS	15	1.66 (0.26 to 10.75)	0.60				
Social isolation	Depression	PRESSO - raw	15	1.42 (0.22 to 9.21)	0.72			Global = 48.09; p=<5e-04, Distortion = -30.19; p=0.85	
Social isolation	Depression	PRESSO - outlier corrected	13 (2 removed)	1.65 (0.38 to 7.09)	0.52				
Social isolation	Depression	MR-CAUSE[a]		1.97 (0.77 to 5.37)				Causal vs sharing model ELPD: delta (SE) = -0.14 (1.21); p=0.45	
Social isolation	Anxiety	Inverse variance weighted	15	0.42 (0.01 to 13.32)	0.62	0.73			80.00
Social isolation	Anxiety	MR-Egger	15	0.02 (1.97e-09 to 124753.93)	0.62	0.67	0.02 (-0.06 to 0.09, 0.68)		
Social isolation	Anxiety	SIMEX adjusted MR-Egger	15	2.28e-03 (1.84e-11 to 283669.57)	0.53		0.02 (-0.06 to 0.11, 0.58)		
Social isolation	Anxiety	Weighted median	15	0.30 (3.25e-03 to 27.96)	0.60				

Exposure	Outcome	Method	N genetic variants	OR or mean difference (95% CI)	p-value	Heterogeneity test p-value	Directional pleiotropy intercept (95% CI; p-value)	Additional tests for MR approaches	Steiger filtering - percentage of genetic variants
Social isolation	Anxiety	MR-RAPS	15	0.45 (0.01 to 17.50)	0.67				
Social isolation	Anxiety	PRESSO - raw	15	0.42 (0.02 to 8.30)	0.58			Global = 11.70; p=0.74, Distortion = NA; p=NA	
Social isolation	Anxiety	PRESSO - outlier corrected	15 (0 removed)	NA (NA to NA)					
Social isolation	Anxiety	MR-CAUSE[a]		0.31 (0.02 to 4.57)				Causal vs sharing model ELPD: delta (SE) = 0.43 (0.83); p=0.70	
Social isolation	Wellbeing spectrum	Inverse variance weighted	14	-0.30 (-0.65 to 0.04)	0.08	6.39e-18			100.00
Social isolation	Wellbeing spectrum	MR-Egger	14	-1.01 (-2.55 to 0.52)	0.22	6.12e-17	3.38e-03 (-3.78e-03 to 0.01, 0.37)		
Social isolation	Wellbeing spectrum	SIMEX adjusted MR-Egger	14	-0.82 (-2.23 to 0.59)	0.27		2.62e-03 (-4.40e-03 to 9.65e-03, 0.48)		
Social isolation	Wellbeing spectrum	Weighted median	14	-0.30 (-0.52 to -0.08)	8.06e-03				

Exposure	Outcome	Method	N genetic variants	OR or mean difference (95% CI)	p-value	Heterogeneity test p-value	Directional pleiotropy intercept (95% CI; p-value)	Additional tests for MR approaches	Steiger filtering - percentage of genetic variants
Social isolation	Wellbeing spectrum	MR-RAPS	14	-0.35 (-0.64 to -0.05)	0.02				
Social isolation	Wellbeing spectrum	PRESSO - raw	14	-0.30 (-0.65 to 0.04)	0.11			Global = 129.90; p=<5e-04, Distortion = 21.15; p=0.49	
Social isolation	Wellbeing spectrum	PRESSO - outlier corrected	9 (5 removed)	-0.39 (-0.63 to -0.14)	0.01				
Social isolation	Wellbeing spectrum	MRLAP - observed	14	-0.10 (-0.21 to 0.01)	0.08				
Social isolation	Wellbeing spectrum	MRLAP - corrected	14	-0.13 (-0.29 to 0.04)	0.14			Corrected vs observed estimate difference test = 1.05; p=0.30	
Social isolation	Wellbeing spectrum	MR-CAUSE[a]		-0.26 (-0.39 to -0.12)				Causal vs sharing model ELPD: delta (SE) = -4.44 (2.05); p=0.02	
Social isolation	Positive affect	Inverse variance weighted	14	-0.69 (-1.28 to -0.11)	0.02	1.47e-07			100.00
Social isolation	Positive affect	MR-Egger	14	-2.02 (-4.62 to 0.58)	0.15	4.33e-07	6.32e-03 (-5.79e-03 to 0.02, 0.33)		

Exposure	Outcome	Method	N genetic variants	OR or mean difference (95% CI)	p-value	Heterogeneity test p-value	Directional pleiotropy intercept (95% CI; p-value)	Additional tests for MR approaches	Steiger filtering - percentage of genetic variants
Social isolation	Positive affect	SIMEX adjusted MR-Egger	14	-0.85 (-3.73 to 2.02)	0.57		1.16e-03 (-0.01 to 0.02, 0.88)		
Social isolation	Positive affect	Weighted median	14	-0.30 (-0.75 to 0.14)	0.18				
Social isolation	Positive affect	MR-RAPS	14	-0.39 (-0.74 to -0.03)	0.03				
Social isolation	Positive affect	PRESSO - raw	14	-0.69 (-1.28 to -0.11)	0.04			Global = 69.75; p=<5e-04, Distortion = -78.10; p=0.02	
Social isolation	Positive affect	PRESSO - outlier corrected	13 (1 removed)	-0.39 (-0.78 to -2.34e-03)	0.07				
Social isolation	Positive affect	MRLAP - observed	14	-0.22 (-0.41 to -0.04)	0.02				
Social isolation	Positive affect	MRLAP - corrected	14	-0.29 (-0.57 to -0.02)	0.03			Corrected vs observed estimate difference test = 1.47; p=0.14	
Social isolation	Positive affect	MR-CAUSE[a]		-0.46 (-0.69 to -0.22)				Causal vs sharing model ELPD: delta (SE) = -4.58 (2.05); p=0.01	

Exposure	Outcome	Method	N genetic variants	OR or mean difference (95% CI)	p-value	Heterogeneity test p-value	Directional pleiotropy intercept (95% CI; p-value)	Additional tests for MR approaches	Steiger filtering - percentage of genetic variants
Social isolation	Life satisfaction	Inverse variance weighted	14	-0.40 (-1.03 to 0.23)	0.21	0.44			100.00
Social isolation	Life satisfaction	MR-Egger	14	-2.37 (-5.18 to 0.44)	0.12	0.51	9.37e-03 (-3.67e-03 to 0.02, 0.18)		
Social isolation	Life satisfaction	SIMEX adjusted MR-Egger	14	-1.57 (-4.40 to 1.26)	0.30		5.98e-03 (-8.06e-03 to 0.02, 0.42)		
Social isolation	Life satisfaction	Weighted median	14	-0.70 (-1.57 to 0.17)	0.12				
Social isolation	Life satisfaction	MR-RAPS	14	-0.46 (-1.12 to 0.21)	0.18				
Social isolation	Life satisfaction	PRESSO - raw	14	-0.40 (-1.03 to 0.23)	0.23			Global = 15.43; p=0.44, Distortion = NA; p=NA	
Social isolation	Life satisfaction	PRESSO - outlier corrected	14 (0 removed)	NA (NA to NA)					
Social isolation	Life satisfaction	MR-CAUSE[a]		-0.42 (-0.82 to -0.03)				Causal vs sharing model ELPD: delta (SE) = -1.32 (1.66); p=0.21	

OR=odds ratio, CI=confidence intervals, MR=Mendelian Randomisation, SIMEX= simulation extrapolation, MR-RAPS= MR using robust adjusted profile score, PRESSO=Pleiotropy RESidual Sum and Outlier, CAD=coronary artery disease, HF=heart failure, T2D=type 2 diabetes, SBP=systolic blood pressure, ELPD=expected log pointwise posterior density, SE=standard error

[a] *MR-CAUSE is a Bayesian method so the values reported are the posterior median as the estimate and the posterior credible interval as the CI.*

The two-sample Mendelian Randomisation approach used is indicated in the table and all tests were two-tailed.

Table S14. Two-sample Mendelian Randomisation analysis results with loneliness and social isolation as outcomes

Exposure	Outcome	Method	N genetic variants	OR or mean difference (95% CI)	p-value	Heterogeneity test p-value	Directional pleiotropy intercept (95% CI; p-value)	Additional tests for MR approaches	Steiger filtering - percentage of genetic variants
CAD	Loneliness	Inverse variance weighted	137	1.00 (0.99 to 1.02)	0.57	1.11e-06			100.00
CAD	Loneliness	MR-Egger	137	1.00 (0.97 to 1.02)	0.80	9.72e-07	5.00e-04 (-1.04e-03 to 2.04e-03, 0.53)		
CAD	Loneliness	SIMEX adjusted MR-Egger	137	1.01 (0.98 to 1.03)	0.54		1.48e-04 (-1.55e-03 to 1.85e-03, 0.86)		
CAD	Loneliness	Weighted median	137	1.00 (0.98 to 1.02)	0.78				
CAD	Loneliness	MR-RAPS	137	1.00 (0.99 to 1.02)	0.54				
CAD	Loneliness	PRESSO - raw	137	1.00 (0.99 to 1.02)	0.57			Global = 232.12; p=<0.0003, Distortion = -45.73; p=0.70	
CAD	Loneliness	PRESSO - outlier corrected	134 (3 removed)	1.01 (0.99 to 1.02)	0.27				
CAD	Loneliness	MRLAP - observed	177	1.02 (0.99 to 1.05)	0.17				
CAD	Loneliness	MRLAP - corrected	177	1.02 (0.98 to 1.06)	0.30			Corrected vs observed estimate	

Exposure	Outcome	Method	N genetic variants	OR or mean difference (95% CI)	p-value	Heterogeneity test p-value	Directional pleiotropy intercept (95% CI; p-value)	Additional tests for MR approaches	Steiger filtering - percentage of genetic variants
								difference test = 1.19; p=0.23	
CAD	Loneliness	MR-CAUSE[a]		1.01 (1.00 to 1.03)				Causal vs sharing model ELPD: delta (SE) = -0.83 (1.39); p=0.28	
CAD	Social isolation	Inverse variance weighted	138	-6.70e-04 (-3.51e-03 to 2.17e-03)	0.64	1.46e-03			100.00
CAD	Social isolation	MR-Egger	138	3.48e-03 (-2.16e-03 to 9.11e-03)	0.23	2.21e-03	-2.70e-04 (-5.87e-04 to 4.73e-05, 0.10)		
CAD	Social isolation	SIMEX adjusted MR-Egger	138	2.92e-03 (-2.06e-03 to 7.91e-03)	0.25		-2.07e-04 (-5.50e-04 to 1.36e-04, 0.24)		
CAD	Social isolation	Weighted median	138	1.27e-03 (-3.13e-03 to 5.67e-03)	0.57				
CAD	Social isolation	MR-RAPS	138	-5.53e-04 (-3.29e-03 to 2.19e-03)	0.69				
CAD	Social isolation	PRESSO - raw	138	-6.70e-04 (-3.51e-03 to 2.17e-03)	0.64			Global = 194.40; p=3.33e-04, Distortion = -13.97; p=0.95	

Exposure	Outcome	Method	N genetic variants	OR or mean difference (95% CI)	p-value	Heterogeneity test p-value	Directional pleiotropy intercept (95% CI; p-value)	Additional tests for MR approaches	Steiger filtering - percentage of genetic variants
CAD	Social isolation	PRESSO - outlier corrected	136 (2 removed)	-5.88e-04 (-3.25e-03 to 2.08e-03)	0.67				
CAD	Social isolation	MRLAP - observed	177	-3.46e-03 (-0.03 to 0.03)	0.83				
CAD	Social isolation	MRLAP - corrected	177	-4.28e-03 (-0.04 to 0.03)	0.82			Corrected vs observed estimate difference test = 2.79; p=5.20e-03	
CAD	Social isolation	MR-CAUSE[a]		0.00 (0.00 to 0.00)				Causal vs sharing model ELPD: delta (SE) = 0.97 (0.36); p=1.00	
Systolic blood pressure	Loneliness	Inverse variance weighted	94	1.00 (1.00 to 1.00)	0.78	3.59e-04			100.00
Systolic blood pressure	Loneliness	MR-Egger	94	1.00 (0.99 to 1.02)	0.58	3.22e-04	-1.08e-03 (-4.27e-03 to 2.11e-03, 0.51)		
Systolic blood pressure	Loneliness	SIMEX adjusted MR-Egger	94	1.01 (0.99 to 1.02)	0.54		-1.35e-03 (-5.25e-03 to 2.55e-03, 0.50)		

Exposure	Outcome	Method	N genetic variants	OR or mean difference (95% CI)	p-value	Heterogeneity test p-value	Directional pleiotropy intercept (95% CI; p-value)	Additional tests for MR approaches	Steiger filtering - percentage of genetic variants
Systolic blood pressure	Loneliness	Weighted median	94	1.00 (1.00 to 1.00)	0.90				
Systolic blood pressure	Loneliness	MR-RAPS	94	1.00 (1.00 to 1.00)	0.95				
Systolic blood pressure	Loneliness	PRESSO - raw	94	1.00 (1.00 to 1.00)	0.78			Global = 149.16; p=<5e-04, Distortion = NA; p=NA	
Systolic blood pressure	Loneliness	PRESSO - outlier corrected	93 (1 removed)	NA (NA to NA)					
Systolic blood pressure	Loneliness	MRLAP - observed	400	0.99 (0.97 to 1.00)	0.16				
Systolic blood pressure	Loneliness	MRLAP - corrected	400	0.99 (0.97 to 1.01)	0.29			Corrected vs observed estimate difference test = -0.70; p=0.48	
Systolic blood pressure	Loneliness	MR-CAUSE[a]		1.00 (1.00 to 1.00)				Causal vs sharing model ELPD: delta (SE) = 0.53 (0.79); p=0.75	

Exposure	Outcome	Method	N genetic variants	OR or mean difference (95% CI)	p-value	Heterogeneity test p-value	Directional pleiotropy intercept (95% CI; p-value)	Additional tests for MR approaches	Steiger filtering - percentage of genetic variants
Systolic blood pressure	Social isolation	Inverse variance weighted	96	-7.03e-04 (-1.50e-03 to 9.54e-05)	0.08	2.76e-03			100.00
Systolic blood pressure	Social isolation	MR-Egger	96	-1.78e-04 (-3.29e-03 to 2.94e-03)	0.91	2.30e-03	-1.30e-04 (-8.74e-04 to 6.14e-04, 0.73)		
Systolic blood pressure	Social isolation	SIMEX adjusted MR-Egger	96	-1.65e-03 (-3.56e-03 to 2.58e-04)	0.09		2.22e-04 (-3.29e-04 to 7.73e-04, 0.43)		
Systolic blood pressure	Social isolation	Weighted median	96	-8.82e-04 (-1.83e-03 to 6.98e-05)	0.07				
Systolic blood pressure	Social isolation	MR-RAPS	96	-8.56e-04 (-1.56e-03 to -1.50e-04)	0.02				
Systolic blood pressure	Social isolation	PRESSO - raw	96	-7.03e-04 (-1.50e-03 to 9.54e-05)	0.09			Global = 140.83; p=2.00e-03, Distortion = 8.50; p=0.87	
Systolic blood pressure	Social isolation	PRESSO - outlier corrected	94 (2 removed)	-7.69e-04 (-1.47e-03 to -6.96e-05)	0.03				

Exposure	Outcome	Method	N genetic variants	OR or mean difference (95% CI)	p-value	Heterogeneity test p-value	Directional pleiotropy intercept (95% CI; p-value)	Additional tests for MR approaches	Steiger filtering - percentage of genetic variants
Systolic blood pressure	Social isolation	MRLAP - observed	441	0.02 (-1.77e-03 to 0.03)	0.08				
Systolic blood pressure	Social isolation	MRLAP - corrected	441	0.02 (-1.59e-03 to 0.04)	0.07			Corrected vs observed estimate difference test = -1.63; p=0.10	
Systolic blood pressure	Social isolation	MR-CAUSE[a]		0.00 (0.00 to 0.00)				Causal vs sharing model ELPD: delta (SE) = 0.66 (0.55); p=0.89	
Heart failure	Loneliness	Inverse variance weighted	9	1.00 (0.94 to 1.06)	0.87	0.02			100.00
Heart failure	Loneliness	MR-Egger	9	0.89 (0.73 to 1.08)	0.26	0.03	7.66e-03 (-4.68e-03 to 0.02, 0.26)		
Heart failure	Loneliness	SIMEX adjusted MR-Egger	9	0.96 (0.79 to 1.17)	0.70		1.40e-03 (-0.01 to 0.01, 0.84)		
Heart failure	Loneliness	Weighted median	9	0.98 (0.93 to 1.04)	0.56				
Heart failure	Loneliness	MR-RAPS	9	0.99 (0.94 to 1.04)	0.68				

Exposure	Outcome	Method	N genetic variants	OR or mean difference (95% CI)	p-value	Heterogeneity test p-value	Directional pleiotropy intercept (95% CI; p-value)	Additional tests for MR approaches	Steiger filtering - percentage of genetic variants
Heart failure	Loneliness	PRESSO - raw	9	1.00 (0.94 to 1.06)	0.87			Global = 22.64; p=0.03, Distortion = -222.74; p=0.12	
Heart failure	Loneliness	PRESSO - outlier corrected	7 (2 removed)	1.00 (0.96 to 1.04)	0.94				
Heart failure	Loneliness	MRLAP - observed	12	1.01 (0.82 to 1.25)	0.91				
Heart failure	Loneliness	MRLAP - corrected	12	1.01 (0.75 to 1.35)	0.96			Corrected vs observed estimate difference test = 0.11; p=0.91	
Heart failure	Loneliness	MR-CAUSE[a]		1.00 (0.97 to 1.04)				Causal vs sharing model ELPD: delta (SE) = 0.87 (0.18); p=1.00	
Heart failure	Social isolation	Inverse variance weighted	9	4.39e-03 (-4.30e-03 to 0.01)	0.32	0.68			100.00
Heart failure	Social isolation	MR-Egger	9	4.61e-03 (-0.03 to 0.03)	0.77	0.57	-1.44e-05 (-1.89e-03 to 1.86e-03, 0.99)		

Exposure	Outcome	Method	N genetic variants	OR or mean difference (95% CI)	p-value	Heterogeneity test p-value	Directional pleiotropy intercept (95% CI; p-value)	Additional tests for MR approaches	Steiger filtering - percentage of genetic variants
Heart failure	Social isolation	SIMEX adjusted MR-Egger	9	2.90e-03 (-0.02 to 0.03)	0.81		1.87e-05 (-1.55e-03 to 1.59e-03, 0.98)		
Heart failure	Social isolation	Weighted median	9	4.13e-03 (-7.25e-03 to 0.02)	0.48				
Heart failure	Social isolation	MR-RAPS	9	3.08e-03 (-5.11e-03 to 0.01)	0.46				
Heart failure	Social isolation	PRESSO - raw	9	4.39e-03 (-2.96e-03 to 0.01)	0.28			Global = 7.00; p=0.71, Distortion = NA; p=NA	
Heart failure	Social isolation	PRESSO - outlier corrected	9 (0 removed)	NA (NA to NA)					
Heart failure	Social isolation	MRLAP - observed	12	0.09 (-0.04 to 0.22)	0.16				
Heart failure	Social isolation	MRLAP - corrected	12	0.12 (-0.05 to 0.29)	0.18			Corrected vs observed estimate difference test = -1.19; p=0.23	
Heart failure	Social isolation	MR-CAUSE[a]		0.01 (0.00 to 0.01)				Causal vs sharing model ELPD: delta (SE) = -0.98 (1.51); p=0.26	

Exposure	Outcome	Method	N genetic variants	OR or mean difference (95% CI)	p-value	Heterogeneity test p-value	Directional pleiotropy intercept (95% CI; p-value)	Additional tests for MR approaches	Steiger filtering - percentage of genetic variants
Stroke	Loneliness	Inverse variance weighted	25	0.99 (0.95 to 1.02)	0.52	5.73e-03			92.00
Stroke	Loneliness	MR-Egger	25	0.95 (0.85 to 1.06)	0.35	5.47e-03	2.30e-03 (-3.38e-03 to 7.98e-03, 0.44)		
Stroke	Loneliness	SIMEX adjusted MR-Egger	25	0.91 (0.78 to 1.06)	0.26		4.28e-03 (-3.89e-03 to 0.01, 0.32)		
Stroke	Loneliness	Weighted median	25	0.99 (0.96 to 1.03)	0.77				
Stroke	Loneliness	MR-RAPS	25	0.99 (0.95 to 1.03)	0.62				
Stroke	Loneliness	PRESSO - raw	25	0.99 (0.95 to 1.02)	0.53			Global = 49.43; p=6.00e-03, Distortion = -250.78; p=0.19	
Stroke	Loneliness	PRESSO - outlier corrected	23 (2 removed)	1.00 (0.97 to 1.02)	0.81				
Stroke	Loneliness	MRLAP - observed	25	0.95 (0.80 to 1.13)	0.56				
Stroke	Loneliness	MRLAP - corrected	25	0.93 (0.74 to 1.17)	0.56			Corrected vs observed estimate	

Exposure	Outcome	Method	N genetic variants	OR or mean difference (95% CI)	p-value	Heterogeneity test p-value	Directional pleiotropy intercept (95% CI; p-value)	Additional tests for MR approaches	Steiger filtering - percentage of genetic variants
								difference test = 0.85; p=0.40	
Stroke	Loneliness	MR-CAUSE[a]		1.01 (0.97 to 1.04)				Causal vs sharing model ELPD: delta (SE) = 0.71 (0.31); p=0.99	
Stroke	Social isolation	Inverse variance weighted	25	9.54e-04 (-5.75e-03 to 7.66e-03)	0.78	0.16			96.00
Stroke	Social isolation	MR-Egger	25	-8.60e-03 (-0.03 to 0.01)	0.41	0.16	5.29e-04 (-5.13e-04 to 1.57e-03, 0.33)		
Stroke	Social isolation	SIMEX adjusted MR-Egger	25	-8.49e-03 (-0.04 to 0.02)	0.55		4.83e-04 (-9.72e-04 to 1.94e-03, 0.52)		
Stroke	Social isolation	Weighted median	25	2.75e-03 (-6.07e-03 to 0.01)	0.54				
Stroke	Social isolation	MR-RAPS	25	1.37e-03 (-5.85e-03 to 8.59e-03)	0.71				
Stroke	Social isolation	PRESSO - raw	25	9.54e-04 (-5.75e-03 to 7.66e-03)	0.78			Global = 33.68; p=0.15, Distortion = NA; p=NA	
Stroke	Social isolation	PRESSO - outlier corrected	25 (0 removed)	NA (NA to NA)					

Exposure	Outcome	Method	N genetic variants	OR or mean difference (95% CI)	p-value	Heterogeneity test p-value	Directional pleiotropy intercept (95% CI; p-value)	Additional tests for MR approaches	Steiger filtering - percentage of genetic variants
Stroke	Social isolation	MRLAP - observed	25	0.02 (-0.13 to 0.18)	0.77				
Stroke	Social isolation	MRLAP - corrected	25	0.03 (-0.18 to 0.23)	0.80			Corrected vs observed estimate difference test = -0.15; p=0.88	
Stroke	Social isolation	MR-CAUSE[a]		0.00 (-0.01 to 0.01)				Causal vs sharing model ELPD: delta (SE) = 0.74 (0.17); p=1.00	
T2D	Loneliness	Inverse variance weighted	226	1.01 (1.00 to 1.02)	0.06	2.66e-21			99.12
T2D	Loneliness	MR-Egger	226	0.98 (0.95 to 1.00)	0.05	8.61e-19	2.23e-03 (9.20e-04 to 3.54e-03, 9.90e-04)		
T2D	Loneliness	SIMEX adjusted MR-Egger	226	0.98 (0.95 to 1.00)	0.06		2.21e-03 (8.76e-04 to 3.55e-03, 1.34e-03)		
T2D	Loneliness	Weighted median	226	0.99 (0.98 to 1.00)	0.20				
T2D	Loneliness	MR-RAPS	226	1.01 (1.00 to 1.02)	0.11				

Exposure	Outcome	Method	N genetic variants	OR or mean difference (95% CI)	p-value	Heterogeneity test p-value	Directional pleiotropy intercept (95% CI; p-value)	Additional tests for MR approaches	Steiger filtering - percentage of genetic variants
T2D	Loneliness	PRESSO - raw	226	1.01 (1.00 to 1.02)	0.07			Global = 492.23; p=<2e-04, Distortion = 22.69; p=0.70	
T2D	Loneliness	PRESSO - outlier corrected	220 (6 removed)	1.01 (1.00 to 1.02)	0.09				
T2D	Loneliness	MRLAP - observed	302	1.04 (1.01 to 1.06)	1.52e-03				
T2D	Loneliness	MRLAP - corrected	302	1.04 (1.01 to 1.07)	5.99e-03			Corrected vs observed estimate difference test = 0.27; p=0.79	
T2D	Loneliness	MR-CAUSE[a]		1.02 (1.00 to 1.03)				Causal vs sharing model ELPD: delta (SE) = -1.02 (1.88); p=0.29	
T2D	Social isolation	Inverse variance weighted	227	-9.10e-05 (-2.61e-03 to 2.43e-03)	0.94	1.67e-13			99.12
T2D	Social isolation	MR-Egger	227	4.71e-03 (-3.03e-04 to 9.71e-03)	0.07	8.87e-13	-3.02e-04 (-5.76e-04 to -2.89e-05, 0.03)		

Exposure	Outcome	Method	N genetic variants	OR or mean difference (95% CI)	p-value	Heterogeneity test p-value	Directional pleiotropy intercept (95% CI; p-value)	Additional tests for MR approaches	Steiger filtering - percentage of genetic variants
T2D	Social isolation	SIMEX adjusted MR-Egger	227	4.39e-03 (-8.30e-04 to 9.62e-03)	0.10		-2.72e-04 (-5.53e-04 to 9.38e-06, 0.06)		
T2D	Social isolation	Weighted median	227	1.12e-03 (-2.45e-03 to 4.68e-03)	0.54				
T2D	Social isolation	MR-RAPS	227	2.35e-04 (-2.18e-03 to 2.65e-03)	0.85				
T2D	Social isolation	PRESSO - raw	227	-9.10e-05 (-2.61e-03 to 2.43e-03)	0.94			Global = 420.16; p=<2e-04, Distortion = -120.84; p=0.17	
T2D	Social isolation	PRESSO - outlier corrected	221 (6 removed)	4.37e-04 (-1.83e-03 to 2.70e-03)	0.71				
T2D	Social isolation	MRLAP - observed	248	0.01 (-0.01 to 0.04)	0.29				
T2D	Social isolation	MRLAP - corrected	248	0.01 (-0.02 to 0.04)	0.37			Corrected vs observed estimate difference test = 0.12; p=0.90	
T2D	Social isolation	MR-CAUSE[a]		0.00 (0.00 to 0.00)				Causal vs sharing model ELPD: delta (SE) = 0.79 (0.43); p=0.97	

Exposure	Outcome	Method	N genetic variants	OR or mean difference (95% CI)	p-value	Heterogeneity test p-value	Directional pleiotropy intercept (95% CI; p-value)	Additional tests for MR approaches	Steiger filtering - percentage of genetic variants
Suicide attempt[b]	Loneliness	Inverse variance weighted	14	1.00 (1.00 to 1.00)	0.48	3.17e-04			100.00
Suicide attempt	Loneliness	MR-Egger	14	1.08 (1.04 to 1.12)	9.22e-04	0.25	-0.08 (-0.11 to -0.04, 1.02e-03)		
Suicide attempt	Loneliness	SIMEX adjusted MR-Egger	14	1.10 (0.95 to 1.28)	0.22		-1.27e-03 (-0.01 to 8.09e-03, 0.80)		
Suicide attempt	Loneliness	Weighted median	14	1.00 (1.00 to 1.00)	0.56				
Suicide attempt	Loneliness	MR-RAPS	14	1.08 (1.04 to 1.12)	3.88e-05				
Suicide attempt	Loneliness	PRESSO - raw	14	1.08 (1.05 to 1.12)	4.61e-04			Global = 17.43; p=0.37, Distortion = NA; p=NA	
Suicide attempt	Loneliness	PRESSO - outlier corrected	14 (0 removed)	NA (NA to NA)					
Suicide attempt	Loneliness	MRLAP - observed	14	1.23 (1.09 to 1.38)	6.38e-04				
Suicide attempt	Loneliness	MRLAP - corrected	14	1.43 (1.15 to 1.77)	1.29e-03			Corrected vs observed estimate	

Exposure	Outcome	Method	N genetic variants	OR or mean difference (95% CI)	p-value	Heterogeneity test p-value	Directional pleiotropy intercept (95% CI; p-value)	Additional tests for MR approaches	Steiger filtering - percentage of genetic variants
								difference test = -3.01; p=2.60e-03	
Suicide attempt	Loneliness	MR-CAUSE[a]		1.04 (1.00 to 1.08)				Causal vs sharing model ELPD: delta (SE) = -1.61 (1.59); p=0.16	
Suicide attempt[b]	Social isolation	Inverse variance weighted	14	1.55e-05 (-6.71e-04 to 7.02e-04)	0.96	4.86e-04			100.00
Suicide attempt	Social isolation	MR-Egger	14	0.01 (7.56e-04 to 0.02)	0.06	8.57e-03	-0.01 (-0.02 to -7.74e-04, 0.06)		
Suicide attempt	Social isolation	SIMEX adjusted MR-Egger	14	0.01 (7.64e-04 to 0.02)	0.06		-0.01 (-0.02 to -7.84e-04, 0.06)		
Suicide attempt	Social isolation	Weighted median	14	1.04e-05 (-6.13e-04 to 6.34e-04)	0.97				
Suicide attempt	Social isolation	MR-RAPS	14	1.10e-04 (-4.30e-04 to 6.50e-04)	0.69				
Suicide attempt	Social isolation	PRESSO - raw	14	1.55e-05 (-6.71e-04 to 7.02e-04)	0.97			Global = 43.96; p=5.00e-04, Distortion = 134.99; p=0.09	

Exposure	Outcome	Method	N genetic variants	OR or mean difference (95% CI)	p-value	Heterogeneity test p-value	Directional pleiotropy intercept (95% CI; p-value)	Additional tests for MR approaches	Steiger filtering - percentage of genetic variants
Suicide attempt	Social isolation	PRESSO - outlier corrected	12 (2 remove d)	-4.44e-05 (-5.12e-04 to 4.24e-04)	0.86				
Suicide attempt	Social isolation	MRLAP - observed	11	0.14 (-1.42e-03 to 0.28)	0.05				
Suicide attempt	Social isolation	MRLAP - corrected	11	0.26 (-0.02 to 0.54)	0.07			Corrected vs observed estimate difference test = -1.64; p=0.10	
Suicide attempt	Social isolation	MR-CAUSE[a]		0.01 (0.00 to 0.01)				Causal vs sharing model ELPD: delta (SE) = -0.86 (1.35); p=0.26	
Depression	Loneliness	Inverse variance weighted	29	1.20 (1.14 to 1.27)	1.96e-11	1.20e-04			100.00
Depression	Loneliness	MR-Egger	29	1.01 (0.86 to 1.18)	0.90	1.68e-03	6.12e-03 (8.82e-04 to 0.01, 0.03)		
Depression	Loneliness	SIMEX adjusted MR-Egger	29	1.02 (0.84 to 1.25)	0.84		5.76e-03 (-9.12e-04 to 0.01, 0.10)		
Depression	Loneliness	Weighted median	29	1.12 (1.05 to 1.19)	3.20e-04				

Exposure	Outcome	Method	N genetic variants	OR or mean difference (95% CI)	p-value	Heterogeneity test p-value	Directional pleiotropy intercept (95% CI; p-value)	Additional tests for MR approaches	Steiger filtering - percentage of genetic variants
Depression	Loneliness	MR-RAPS	29	1.21 (1.14 to 1.28)	4.17e-11				
Depression	Loneliness	PRESSO - raw	29	1.20 (1.14 to 1.27)	2.78e-07			Global = 68.96; p=8.00e-03, Distortion = 5.49; p=0.71	
Depression	Loneliness	PRESSO - outlier corrected	28 (1 removed)	1.19 (1.13 to 1.25)	1.82e-07				
Depression	Loneliness	MRLAP - observed	2	1.26 (0.88 to 1.80)	0.20				
Depression	Loneliness	MRLAP - corrected	2	1.54 (0.79 to 3.01)	0.20			Corrected vs observed estimate difference test = -1.26; p=0.21	
Depression	Loneliness	MR-CAUSE[a]		1.05 (1.01 to 1.09)				Causal vs sharing model ELPD: delta (SE) = -2.34 (1.74); p=0.09	
Depression	Social isolation	Inverse variance weighted	29	0.02 (4.47e-03 to 0.03)	6.66e-03	2.82e-04			100.00
Depression	Social isolation	MR-Egger	29	0.03 (-9.50e-03 to 0.06)	0.16	2.38e-04	-4.00e-04 (-1.63e-03 to 8.35e-04, 0.53)		

Exposure	Outcome	Method	N genetic variants	OR or mean difference (95% CI)	p-value	Heterogeneity test p-value	Directional pleiotropy intercept (95% CI; p-value)	Additional tests for MR approaches	Steiger filtering - percentage of genetic variants
Depression	Social isolation	SIMEX adjusted MR-Egger	29	0.04 (-0.02 to 0.09)	0.21		-7.83e-04 (-2.57e-03 to 1.00e-03, 0.40)		
Depression	Social isolation	Weighted median	29	7.15e-03 (-5.77e-03 to 0.02)	0.28				
Depression	Social isolation	MR-RAPS	29	0.01 (-9.70e-04 to 0.02)	0.07				
Depression	Social isolation	PRESSO - raw	29	0.02 (4.47e-03 to 0.03)	0.01			Global = 67.67; p=<5e-04, Distortion = 50.49; p=0.12	
Depression	Social isolation	PRESSO - outlier corrected	28 (1 removed)	0.01 (6.85e-04 to 0.02)	0.05				
Depression	Social isolation	MR-CAUSE[a]		0.00 (0.00 to 0.01)				Causal vs sharing model ELPD: delta (SE) = 0.40 (0.82); p=0.69	
Anxiety[c]	Loneliness	Inverse variance weighted	18	1.00 (0.99 to 1.01)	0.88	0.02			100.00
Anxiety	Loneliness	MR-Egger	18	1.01 (0.97 to 1.04)	0.78	0.01	-8.38e-04 (-7.57e-03 to 5.89e-03, 0.81)		

Exposure	Outcome	Method	N genetic variants	OR or mean difference (95% CI)	p-value	Heterogeneity test p-value	Directional pleiotropy intercept (95% CI; p-value)	Additional tests for MR approaches	Steiger filtering - percentage of genetic variants
Anxiety	Loneliness	SIMEX adjusted MR-Egger	18	1.01 (0.96 to 1.06)	0.77		-1.13e-03 (-9.55e-03 to 7.28e-03, 0.79)		
Anxiety	Loneliness	Weighted median	18	1.01 (0.99 to 1.02)	0.36				
Anxiety	Loneliness	MR-RAPS	18	1.00 (0.99 to 1.02)	0.74				
Anxiety	Loneliness	PRESSO - raw	18	1.00 (0.99 to 1.01)	0.88			Global = 35.83; p=0.02, Distortion = NA; p=NA	
Anxiety	Loneliness	PRESSO - outlier corrected	17 (1 removed)	NA (NA to NA)					
Anxiety	Loneliness	MRLAP - observed	15	1.00 (0.98 to 1.03)	0.68				
Anxiety	Loneliness	MRLAP - corrected	15	1.03 (0.58 to 1.84)	0.92			Corrected vs observed estimate difference test = -0.08; p=0.94	
Anxiety	Loneliness	MR-CAUSE[a]		0.97 (0.94 to 1.01)				Causal vs sharing model ELPD: delta (SE) = -1.10 (1.06); p=0.15	

Exposure	Outcome	Method	N genetic variants	OR or mean difference (95% CI)	p-value	Heterogeneity test p-value	Directional pleiotropy intercept (95% CI; p-value)	Additional tests for MR approaches	Steiger filtering - percentage of genetic variants
Anxiety[c]	Social isolation	Inverse variance weighted	20	-9.07e-04 (-2.84e-03 to 1.03e-03)	0.36	0.28			100.00
Anxiety	Social isolation	MR-Egger	20	-1.29e-03 (-6.41e-03 to 3.83e-03)	0.63	0.23	8.16e-05 (-9.20e-04 to 1.08e-03, 0.87)		
Anxiety	Social isolation	SIMEX adjusted MR-Egger	20	-3.03e-03 (-8.20e-03 to 2.14e-03)	0.27		4.19e-04 (-8.10e-04 to 1.65e-03, 0.51)		
Anxiety	Social isolation	Weighted median	20	-1.44e-04 (-2.88e-03 to 2.59e-03)	0.92				
Anxiety	Social isolation	MR-RAPS	20	-1.06e-03 (-3.25e-03 to 1.13e-03)	0.34				
Anxiety	Social isolation	PRESSO - raw	20	-9.07e-04 (-2.84e-03 to 1.03e-03)	0.37			Global = 24.28; p=0.31, Distortion = NA; p=NA	
Anxiety	Social isolation	PRESSO - outlier corrected	20 (0 removed)	NA (NA to NA)					
Anxiety	Social isolation	MR-CAUSE[a]		0.00 (-0.02 to 0.01)				Causal vs sharing model ELPD: delta (SE) = 0.44 (0.21); p=0.98	

Exposure	Outcome	Method	N genetic variants	OR or mean difference (95% CI)	p-value	Heterogeneity test p-value	Directional pleiotropy intercept (95% CI; p-value)	Additional tests for MR approaches	Steiger filtering - percentage of genetic variants
Wellbeing spectrum	Loneliness	Inverse variance weighted	132	0.27 (0.24 to 0.30)	4.41e-136	1.17e-03			69.70
Wellbeing spectrum	Loneliness	MR-Egger	132	0.33 (0.20 to 0.55)	4.07e-05	1.09e-03	-1.26e-03 (-4.65e-03 to 2.13e-03, 0.47)		
Wellbeing spectrum	Loneliness	SIMEX adjusted MR-Egger	132	0.25 (0.13 to 0.47)	2.41e-05		5.60e-04 (-3.66e-03 to 4.78e-03, 0.80)		
Wellbeing spectrum	Loneliness	Weighted median	132	0.30 (0.27 to 0.35)	3.57e-67				
Wellbeing spectrum	Loneliness	MR-RAPS	132	0.26 (0.23 to 0.29)	0.00				
Wellbeing spectrum	Loneliness	PRESSO - raw	132	0.27 (0.24 to 0.30)	2.21e-51			Global = 188.63; p=0.04, Distortion = NA; p=NA	
Wellbeing spectrum	Loneliness	PRESSO - outlier corrected	131 (1 removed)	NA (NA to NA)					
Wellbeing spectrum	Loneliness	MRLAP - observed	120	0.42 (0.40 to 0.45)	6.84e-139				
Wellbeing spectrum	Loneliness	MRLAP - corrected	120	0.41 (0.37 to 0.44)	2.01e-92			Corrected vs observed estimate	

Exposure	Outcome	Method	N genetic variants	OR or mean difference (95% CI)	p-value	Heterogeneity test p-value	Directional pleiotropy intercept (95% CI; p-value)	Additional tests for MR approaches	Steiger filtering - percentage of genetic variants
								difference test = 3.28; p=1.02e-03	
Wellbeing spectrum	Loneliness	MR-CAUSE[a]		0.35 (0.32 to 0.39)				Causal vs sharing model ELPD: delta (SE) = -8.40 (1.16); p=2.74e-13	
Wellbeing spectrum	Social isolation	Inverse variance weighted	133	-0.09 (-0.12 to -0.06)	1.83e-08	1.21e-23			86.47
Wellbeing spectrum	Social isolation	MR-Egger	133	-0.06 (-0.22 to 0.10)	0.48	8.46e-24	-2.24e-04 (-1.27e-03 to 8.22e-04, 0.68)		
Wellbeing spectrum	Social isolation	SIMEX adjusted MR-Egger	133	-0.07 (-0.24 to 0.10)	0.44		-1.83e-04 (-1.36e-03 to 9.95e-04, 0.76)		
Wellbeing spectrum	Social isolation	Weighted median	133	-0.09 (-0.12 to -0.06)	3.49e-08				
Wellbeing spectrum	Social isolation	MR-RAPS	133	-0.10 (-0.13 to -0.07)	1.01e-09				
Wellbeing spectrum	Social isolation	PRESSO - raw	133	-0.09 (-0.12 to -0.06)	1.05e-07			Global = 369.87; p=<0.0003, Distortion = 1.00; p=0.95	

Exposure	Outcome	Method	N genetic variants	OR or mean difference (95% CI)	p-value	Heterogeneity test p-value	Directional pleiotropy intercept (95% CI; p-value)	Additional tests for MR approaches	Steiger filtering - percentage of genetic variants
Wellbeing spectrum	Social isolation	PRESSO - outlier corrected	129 (4 remove d)	-0.09 (-0.12 to -0.06)	2.28e-09				
Wellbeing spectrum	Social isolation	MRLAP - observed	11	0.74 (0.52 to 1.05)	0.09				
Wellbeing spectrum	Social isolation	MRLAP - corrected	11	0.69 (0.37 to 1.29)	0.25			Corrected vs observed estimate difference test = 0.49; p=0.62	
Wellbeing spectrum	Social isolation	MR-CAUSE[a]		-0.07 (-0.11 to -0.03)				Causal vs sharing model ELPD: delta (SE) = -0.73 (2.09); p=0.36	
Positive affect	Loneliness	Inverse variance weighted	92	0.37 (0.33 to 0.41)	2.13e-66	2.02e-09			73.91
Positive affect	Loneliness	MR-Egger	92	0.78 (0.56 to 1.09)	0.14	1.81e-05	-7.08e-03 (-0.01 to -4.09e-03, 1.15e-05)		
Positive affect	Loneliness	SIMEX adjusted MR-Egger	92	0.63 (0.38 to 1.03)	0.07		-5.28e-03 (-9.59e-03 to -9.60e-04, 0.02)		
Positive affect	Loneliness	Weighted median	92	0.43 (0.37 to 0.50)	9.13e-32				

Exposure	Outcome	Method	N genetic variants	OR or mean difference (95% CI)	p-value	Heterogeneity test p-value	Directional pleiotropy intercept (95% CI; p-value)	Additional tests for MR approaches	Steiger filtering - percentage of genetic variants
Positive affect	Loneliness	MR-RAPS	92	0.34 (0.30 to 0.38)	0.00				
Positive affect	Loneliness	PRESSO - raw	92	0.37 (0.33 to 0.41)	2.29e-30			Global = 198.71; p=4.50e-03, Distortion = 2.70; p=0.61	
Positive affect	Loneliness	PRESSO - outlier corrected	91 (1 removed)	0.36 (0.32 to 0.40)	9.97e-32				
Positive affect	Loneliness	MRLAP - observed	9	0.73 (0.65 to 0.81)	1.13e-08				
Positive affect	Loneliness	MRLAP - corrected	9	0.65 (0.55 to 0.78)	1.37e-06			Corrected vs observed estimate difference test = 3.19; p=1.42e-03	
Positive affect	Loneliness	MR-CAUSE[a]		0.73 (0.65 to 0.81)				Causal vs sharing model ELPD: delta (SE) = -5.36 (1.73); p=9.50e-04	
Positive affect	Social isolation	Inverse variance weighted	93	-0.09 (-0.11 to -0.06)	9.96e-11	3.49e-12			93.55
Positive affect	Social isolation	MR-Egger	93	-0.09 (-0.18 to -2.89e-03)	0.05	2.24e-12	2.33e-05 (-7.55e-04 to 8.02e-04, 0.95)		

Exposure	Outcome	Method	N genetic variants	OR or mean difference (95% CI)	p-value	Heterogeneity test p-value	Directional pleiotropy intercept (95% CI; p-value)	Additional tests for MR approaches	Steiger filtering - percentage of genetic variants
Positive affect	Social isolation	SIMEX adjusted MR-Egger	93	-0.10 (-0.23 to 0.02)	0.10		1.33e-04 (-9.95e-04 to 1.26e-03, 0.82)		
Positive affect	Social isolation	Weighted median	93	-0.05 (-0.08 to -0.02)	1.37e-03				
Positive affect	Social isolation	MR-RAPS	93	-0.08 (-0.11 to -0.06)	6.23e-10				
Positive affect	Social isolation	PRESSO - raw	93	-0.09 (-0.11 to -0.06)	4.73e-09			Global = 224.91; p=<5e-04, Distortion = -18.18; p=0.19	
Positive affect	Social isolation	PRESSO - outlier corrected	91 (2 removed)	-0.07 (-0.10 to -0.05)	5.72e-08				
Positive affect	Social isolation	MRLAP - observed	98	-0.28 (-0.36 to -0.20)	1.92e-12				
Positive affect	Social isolation	MRLAP - corrected	98	-0.35 (-0.45 to -0.25)	3.01e-11			Corrected vs observed estimate difference test = 5.33; p=9.77e-08	
Positive affect	Social isolation	MR-CAUSE[a]		-0.02 (-0.05 to 0.00)				Causal vs sharing model ELPD: delta (SE) = -0.87 (1.41); p=0.27	

Exposure	Outcome	Method	N genetic variants	OR or mean difference (95% CI)	p-value	Heterogeneity test p-value	Directional pleiotropy intercept (95% CI; p-value)	Additional tests for MR approaches	Steiger filtering - percentage of genetic variants
Life satisfaction	Loneliness	Inverse variance weighted	73	0.42 (0.38 to 0.47)	5.79e-61	8.07e-06			83.56
Life satisfaction	Loneliness	MR-Egger	73	0.79 (0.64 to 0.99)	0.04	0.06	-6.97e-03 (-9.24e-03 to -4.70e-03, 7.33e-08)		
Life satisfaction	Loneliness	SIMEX adjusted MR-Egger	73	0.61 (0.43 to 0.87)	8.28e-03		-4.38e-03 (-7.91e-03 to -8.57e-04, 0.02)		
Life satisfaction	Loneliness	Weighted median	73	0.55 (0.46 to 0.66)	6.01e-11				
Life satisfaction	Loneliness	MR-RAPS	73	0.36 (0.29 to 0.45)	0.00				
Life satisfaction	Loneliness	PRESSO - raw	73	0.42 (0.38 to 0.47)	3.97e-26			Global = 139.97; p=0.95, Distortion = NA; p=NA	
Life satisfaction	Loneliness	PRESSO - outlier corrected	73 (0 removed)	NA (NA to NA)					
Life satisfaction	Loneliness	MRLAP - observed	75	0.58 (0.55 to 0.62)	5.79e-67				
Life satisfaction	Loneliness	MRLAP - corrected	75	0.49 (0.45 to 0.54)	3.67e-57			Corrected vs observed estimate	

Exposure	Outcome	Method	N genetic variants	OR or mean difference (95% CI)	p-value	Heterogeneity test p-value	Directional pleiotropy intercept (95% CI; p-value)	Additional tests for MR approaches	Steiger filtering - percentage of genetic variants
								difference test = 7.72; p=1.12e-14	
Life satisfaction	Loneliness	MR-CAUSE[a]		0.88 (0.73 to 1.06)				Causal vs sharing model ELPD: delta (SE) = -0.21 (1.02); p=0.42	
Life satisfaction	Social isolation	Inverse variance weighted	73	-0.06 (-0.09 to -0.04)	7.61e-07	1.75e-10			94.52
Life satisfaction	Social isolation	MR-Egger	73	-0.05 (-0.12 to 0.01)	0.12	1.19e-10	-1.15e-04 (-8.19e-04 to 5.89e-04, 0.75)		
Life satisfaction	Social isolation	SIMEX adjusted MR-Egger	73	-0.10 (-0.22 to 0.02)	0.11		3.56e-04 (-8.13e-04 to 1.53e-03, 0.55)		
Life satisfaction	Social isolation	Weighted median	73	-0.04 (-0.07 to -0.01)	6.61e-03				
Life satisfaction	Social isolation	MR-RAPS	73	-0.08 (-0.12 to -0.04)	2.06e-05				
Life satisfaction	Social isolation	PRESSO - raw	73	-0.06 (-0.09 to -0.04)	4.81e-06			Global = 181.40; p=<5e-04, Distortion = -18.75; p=0.29	

Exposure	Outcome	Method	N genetic variants	OR or mean difference (95% CI)	p-value	Heterogeneity test p-value	Directional pleiotropy intercept (95% CI; p-value)	Additional tests for MR approaches	Steiger filtering - percentage of genetic variants
Life satisfaction	Social isolation	PRESSO - outlier corrected	72 (1 remove d)	-0.05 (-0.08 to -0.03)	3.96e-05				
Life satisfaction	Social isolation	MR-CAUSE[a]		0.00 (-0.04 to 0.04)				Causal vs sharing model ELPD: delta (SE) = 0.66 (0.11); p=1.00	

OR=odds ratio, CI=confidence intervals, MR=Mendelian Randomisation, SIMEX= simulation extrapolation, MR-RAPS= MR using robust adjusted profile score, PRESSO=Pleiotropy RESidual Sum and Outlier, CAD=coronary artery disease, HF=heart failure, T2D=type 2 diabetes, SBP=systolic blood pressure, ELPD=expected log pointwise posterior density, SE=standard error

[a] MR-CAUSE is a Bayesian method so the values reported are the posterior median as the estimate and the posterior credible interval as the CI.

[b]Where suicide attempt was the exposure a p-value threshold of 1e-06 was used

[c]Where anxiety was the exposure a p-value threshold of 1e-05 was used

The two-sample Mendelian Randomisation approach used is indicated in the table and all tests were two-tailed.

Table S15. Multivariable Mendelian Randomisation analysis results with loneliness and social isolation as exposures

Exposure	Outcome	Method	OR or mean difference (95% CI)	p-value	Conditional F statistic	Additional tests for MR approaches	Pleiotropy test (p-value)
Loneliness	CAD	Inverse variance weighted	1.21 (0.93 to 1.57)	0.16	17.50		62.91 (1.43e-05)
Social isolation	CAD	Inverse variance weighted	0.66 (0.20 to 2.15)	0.50	16.90		
Loneliness	CAD	Egger - orientation Lone	1.15 (0.63 to 2.09)	0.66			
Social isolation	CAD	Egger - orientation SI	0.81 (0.09 to 7.27)	0.86			
Loneliness	CAD	PRESSO - raw	1.21 (0.93 to 1.57)	0.16		Global = 74.50; p=5.00e-04, Distortion = -2.47; p=1.12	
Social isolation	CAD	PRESSO - raw	0.66 (0.20 to 2.15)	0.50		Global = 74.50; p=5.00e-04, Distortion = -525.28; p=1.12	
Loneliness	CAD	PRESSO - outlier corrected	1.22 (0.96 to 1.55)	0.12			
Social isolation	CAD	PRESSO - outlier corrected	0.94 (0.30 to 2.91)	0.91			
Loneliness	Systolic blood pressure	Inverse variance weighted	-3.33 (-6.95 to 0.29)	0.09	16.32		243.25 (9.29e-40)
Social isolation	Systolic blood pressure	Inverse variance weighted	10.36 (-6.19 to 26.91)	0.23	15.55		
Loneliness	Systolic blood pressure	Egger - orientation Lone	-2.66 (-10.99 to 5.68)	0.54			

Exposure	Outcome	Method	OR or mean difference (95% CI)	p-value	Conditional F statistic	Additional tests for MR approaches	Pleiotropy test (p-value)
Social isolation	Systolic blood pressure	Egger - orientation SI	-14.70 (-44.33 to 14.92)	0.34			
Loneliness	Systolic blood pressure	PRESSO - raw	-3.33 (-6.95 to 0.29)	0.09		Global = 326.95; p=<5e-04, Distortion = -51.02; p=1.18	
Social isolation	Systolic blood pressure	PRESSO - raw	10.36 (-6.19 to 26.91)	0.23		Global = 326.95; p=<5e-04, Distortion = 197.95; p=1.18	
Loneliness	Systolic blood pressure	PRESSO - outlier corrected	-2.20 (-5.91 to 1.51)	0.26			
Social isolation	Systolic blood pressure	PRESSO - outlier corrected	3.48 (-14.29 to 21.24)	0.71			
Loneliness	Heart failure	Inverse variance weighted	1.02 (0.72 to 1.44)	0.93	17.50		40.73 (0.01)
Social isolation	Heart failure	Inverse variance weighted	0.70 (0.15 to 3.40)	0.67	16.90		
Loneliness	Heart failure	Egger - orientation Lone	0.81 (0.36 to 1.79)	0.60			
Social isolation	Heart failure	Egger - orientation SI	5.56 (0.34 to 91.85)	0.24			
Loneliness	Heart failure	PRESSO - raw	1.02 (0.72 to 1.44)	0.93		Global = 49.88; p=0.01, Distortion = NA; p=NA	
Social isolation	Heart failure	PRESSO - raw	0.70 (0.15 to 3.40)	0.67		Global = 49.88; p=0.01, Distortion = NA; p=NA	

Exposure	Outcome	Method	OR or mean difference (95% CI)	p-value	Conditional F statistic	Additional tests for MR approaches	Pleiotropy test (p-value)
Loneliness	Heart failure	PRESSO - outlier corrected	NA (NA to NA)				
Social isolation	Heart failure	PRESSO - outlier corrected	NA (NA to NA)				
Loneliness	Stroke	Inverse variance weighted	0.90 (0.62 to 1.32)	0.60	17.50		36.27 (0.04)
Social isolation	Stroke	Inverse variance weighted	1.57 (0.29 to 8.54)	0.61	16.90		
Loneliness	Stroke	Egger - orientation Lone	0.53 (0.23 to 1.22)	0.15			
Social isolation	Stroke	Egger - orientation SI	63.28 (4.45 to 900.26)	5.53e-03			
Loneliness	Stroke	PRESSO - raw	0.90 (0.62 to 1.32)	0.60		Global = 42.49; p=0.06, Distortion = NA; p=NA	
Social isolation	Stroke	PRESSO - raw	1.57 (0.29 to 8.54)	0.61		Global = 42.49; p=0.06, Distortion = NA; p=NA	
Loneliness	Stroke	PRESSO - outlier corrected	NA (NA to NA)				
Social isolation	Stroke	PRESSO - outlier corrected	NA (NA to NA)				
Loneliness	T2D	Inverse variance weighted	1.22 (0.79 to 1.88)	0.37	17.50		187.47 (9.37e-28)

Exposure	Outcome	Method	OR or mean difference (95% CI)	p-value	Conditional F statistic	Additional tests for MR approaches	Pleiotropy test (p-value)
Social isolation	T2D	Inverse variance weighted	0.62 (0.09 to 4.43)	0.64	16.90		
Loneliness	T2D	Egger - orientation Lone	1.73 (0.65 to 4.61)	0.28			
Social isolation	T2D	Egger - orientation SI	3.14 (0.09 to 107.14)	0.53			
Loneliness	T2D	PRESSO - raw	1.22 (0.79 to 1.88)	0.37		Global = 219.54; p=<5e-04, Distortion = 38.20; p=1.42	
Social isolation	T2D	PRESSO - raw	0.62 (0.09 to 4.43)	0.64		Global = 219.54; p=<5e-04, Distortion = -118.65; p=1.42	
Loneliness	T2D	PRESSO - outlier corrected	1.16 (0.85 to 1.56)	0.36			
Social isolation	T2D	PRESSO - outlier corrected	0.81 (0.20 to 3.31)	0.77			
Loneliness	Suicide attempt	Inverse variance weighted	1.96 (1.07 to 3.60)	0.04	17.50		83.85 (7.49e-09)
Social isolation	Suicide attempt	Inverse variance weighted	2.26 (0.14 to 35.51)	0.57	16.90		
Loneliness	Suicide attempt	Egger - orientation Lone	1.76 (0.42 to 7.28)	0.44			
Social isolation	Suicide attempt	Egger - orientation SI	2.69 (0.02 to 413.49)	0.70			

Exposure	Outcome	Method	OR or mean difference (95% CI)	p-value	Conditional F statistic	Additional tests for MR approaches	Pleiotropy test (p-value)
Loneliness	Suicide attempt	PRESSO - raw	1.96 (1.07 to 3.60)	0.04		Global = 105.22; p=<5e-04, Distortion = -4.05; p=1.00	
Social isolation	Suicide attempt	PRESSO - raw	2.26 (0.14 to 35.51)	0.57		Global = 105.22; p=<5e-04, Distortion = 744.25; p=1.00	
Loneliness	Suicide attempt	PRESSO - outlier corrected	2.02 (1.20 to 3.38)	0.01			
Social isolation	Suicide attempt	PRESSO - outlier corrected	0.88 (0.08 to 9.79)	0.92			
Loneliness	Depression	Inverse variance weighted	2.09 (1.28 to 3.41)	7.17e-03	17.50		64.45 (8.49e-06)
Social isolation	Depression	Inverse variance weighted	0.51 (0.06 to 4.63)	0.55	16.90		
Loneliness	Depression	Egger - orientation Lone	2.12 (0.68 to 6.58)	0.21			
Social isolation	Depression	Egger - orientation SI	0.03 (6.68e-04 to 1.48)	0.09			
Loneliness	Depression	PRESSO - raw	2.09 (1.28 to 3.41)	7.17e-03		Global = 79.99; p=<5e-04, Distortion = 3.16; p=1.00	
Social isolation	Depression	PRESSO - raw	0.51 (0.06 to 4.63)	0.55		Global = 79.99; p=<5e-04, Distortion = -536.56; p=1.00	
Loneliness	Depression	PRESSO - outlier corrected	2.04 (1.32 to 3.17)	4.12e-03			

Exposure	Outcome	Method	OR or mean difference (95% CI)	p-value	Conditional F statistic	Additional tests for MR approaches	Pleiotropy test (p-value)
Social isolation	Depression	PRESSO - outlier corrected	1.17 (0.15 to 9.30)	0.88			
Loneliness	Anxiety	Inverse variance weighted	2.52 (0.98 to 6.50)	0.07	16.61		22.54 (0.43)
Social isolation	Anxiety	Inverse variance weighted	0.10 (1.62e-03 to 6.39)	0.29	16.69		
Loneliness	Anxiety	Egger - orientation Lone	0.94 (0.11 to 7.64)	0.95			
Social isolation	Anxiety	Egger - orientation SI	0.19 (3.89e-05 to 921.42)	0.70			
Loneliness	Anxiety	PRESSO - raw	2.52 (0.98 to 6.50)	0.07		Global = 26.10; p=0.55, Distortion = NA; p=NA	
Social isolation	Anxiety	PRESSO - raw	0.10 (1.62e-03 to 6.39)	0.29		Global = 26.10; p=0.55, Distortion = NA; p=NA	
Loneliness	Anxiety	PRESSO - outlier corrected	NA (NA to NA)				
Social isolation	Anxiety	PRESSO - outlier corrected	NA (NA to NA)				
Loneliness	Wellbeing spectrum	Inverse variance weighted	-0.26 (-0.35 to -0.18)	6.71e-06	16.61		68.45 (8.24e-08)
Social isolation	Wellbeing spectrum	Inverse variance weighted	0.02 (-0.32 to 0.37)	0.89	18.25		

Exposure	Outcome	Method	OR or mean difference (95% CI)	p-value	Conditional F statistic	Additional tests for MR approaches	Pleiotropy test (p-value)
Loneliness	Wellbeing spectrum	Egger - orientation Lone	-0.32 (-0.53 to -0.10)	9.07e-03			
Social isolation	Wellbeing spectrum	Egger - orientation SI	0.10 (-0.55 to 0.74)	0.77			
Loneliness	Wellbeing spectrum	PRESSO - raw	-0.26 (-0.35 to -0.18)	6.71e-06		Global = 152.60; p=<5e-04, Distortion = 13.15; p=1.40	
Social isolation	Wellbeing spectrum	PRESSO - raw	0.02 (-0.32 to 0.37)	0.89		Global = 152.60; p=<5e-04, Distortion = -86.13; p=1.40	
Loneliness	Wellbeing spectrum	PRESSO - outlier corrected	-0.30 (-0.37 to -0.24)	3.41e-08			
Social isolation	Wellbeing spectrum	PRESSO - outlier corrected	0.17 (-0.11 to 0.45)	0.24			
Loneliness	Positive affect	Inverse variance weighted	-0.31 (-0.42 to -0.19)	3.46e-05	16.61		30.72 (0.03)
Social isolation	Positive affect	Inverse variance weighted	-0.30 (-0.77 to 0.16)	0.21	18.25		
Loneliness	Positive affect	Egger - orientation Lone	-0.26 (-0.54 to 0.03)	0.09			
Social isolation	Positive affect	Egger - orientation SI	-0.48 (-1.33 to 0.38)	0.29			
Loneliness	Positive affect	PRESSO - raw	-0.31 (-0.42 to -0.19)	3.46e-05		Global = 46.13; p=0.04, Distortion = -3.12; p=0.89	

Exposure	Outcome	Method	OR or mean difference (95% CI)	p-value	Conditional F statistic	Additional tests for MR approaches	Pleiotropy test (p-value)
Social isolation	Positive affect	PRESSO - raw	-0.30 (-0.77 to 0.16)	0.21		Global = 46.13; p=0.04, Distortion = -365.66; p=0.89	
Loneliness	Positive affect	PRESSO - outlier corrected	-0.30 (-0.36 to -0.23)	4.40e-08			
Social isolation	Positive affect	PRESSO - outlier corrected	-0.07 (-0.34 to 0.21)	0.65			
Loneliness	Life satisfaction	Inverse variance weighted	-0.44 (-0.67 to -0.21)	1.29e-03	16.61		29.09 (0.05)
Social isolation	Life satisfaction	Inverse variance weighted	0.14 (-0.82 to 1.10)	0.78	18.25		
Loneliness	Life satisfaction	Egger - orientation Lone	-0.49 (-1.07 to 0.10)	0.12			
Social isolation	Life satisfaction	Egger - orientation SI	1.28 (-0.40 to 2.96)	0.15			
Loneliness	Life satisfaction	PRESSO - raw	-0.44 (-0.67 to -0.21)	1.29e-03		Global = 37.79; p=0.07, Distortion = NA; p=NA	
Social isolation	Life satisfaction	PRESSO - raw	0.14 (-0.82 to 1.10)	0.78		Global = 37.79; p=0.07, Distortion = NA; p=NA	
Loneliness	Life satisfaction	PRESSO - outlier corrected	NA (NA to NA)				
Social isolation	Life satisfaction	PRESSO - outlier corrected	NA (NA to NA)				

OR=odds ratio, CI=confidence intervals, MR=Mendelian Randomisation, SI=social isolation, PRESSO=Pleiotropy RESidual Sum and Outlier, CAD=Coronary artery disease, HF=heart failure, T2D=type 2 diabetes, SBP=systolic blood pressure

The multivariable Mendelian Randomisation approach used is indicated in the table and all tests were two-tailed.

Supplementary Figures

Figure S1. (a) Manhattan plot and (b) Quantile-Quantile plot from social isolation GWAS (N= 457,086)

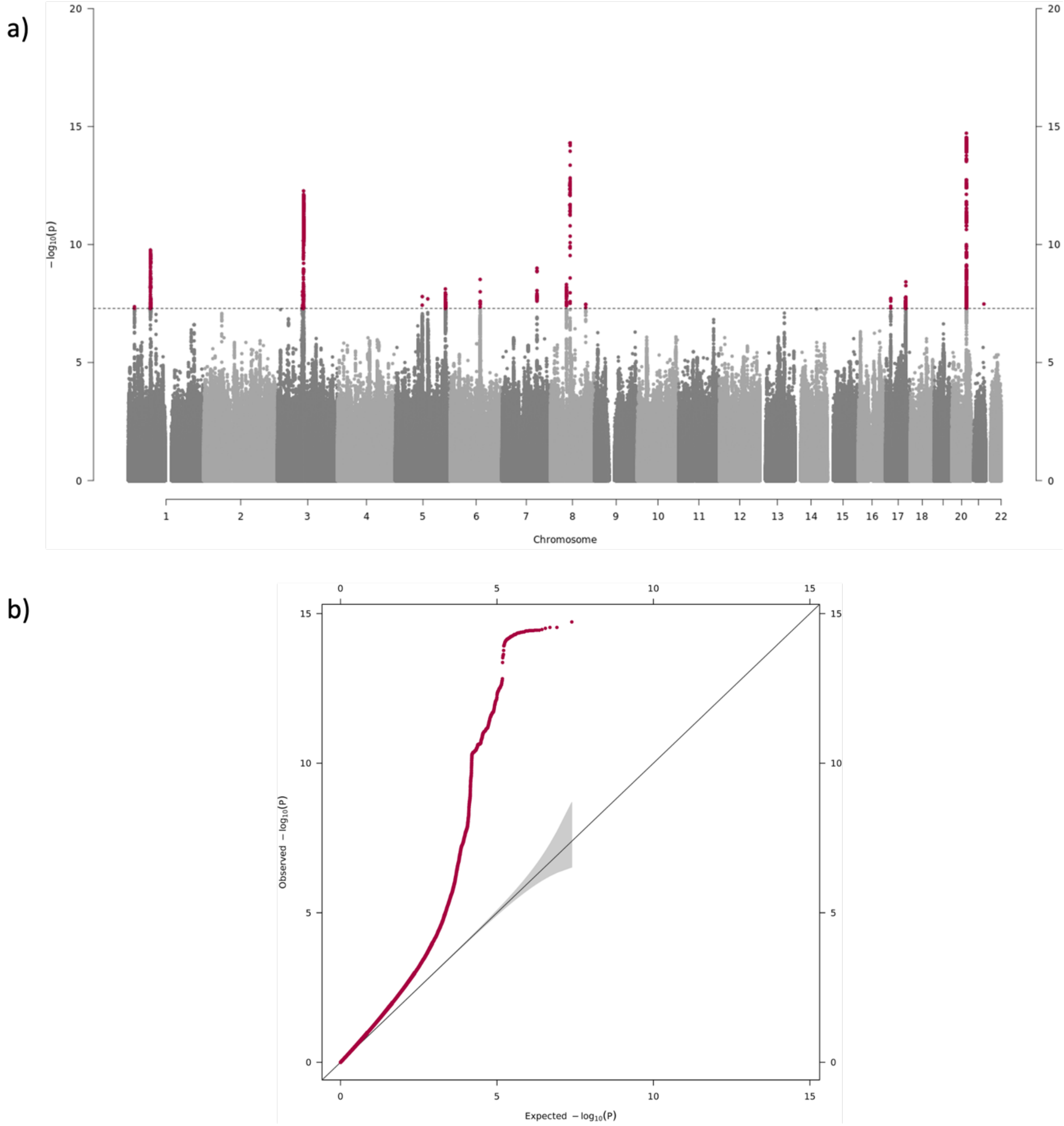

The Manhattan plot (a) shows the genetic variants per chromosome and their respective p-values from the social isolation genome-wide association study (GWAS). The dotted line represents a p-value threshold of 5x10-08. The genetic variants above this line were clumped for independence. The quantile-quantile (QQ) plot presents the observed-values against the expected p-values for the genetic variants.

Supplementary Notes

STROBE-MR checklist of recommended items to address in reports of Mendelian randomization studies

Item No.	Section	Checklist item	Page No.	Relevant text from manuscript
1	**TITLE and ABSTRACT**	Indicate Mendelian randomization (MR) as the study's design in the title and/or the abstract if that is a main purpose of the study	2	This is a triangulation study and therefore MR is not mentioned in the title, but it is mentioned in the abstract: "We use a triangulation approach combining observational, sibling control, and Mendelian Randomisation analyses …"
	**INTRODUCTION**			
2	**Background**	Explain the scientific background and rationale for the reported study. What is the exposure? Is a potential causal relationship between exposure and outcome plausible? Justify why MR is a helpful method to address the study question	3	The exposures are loneliness and social isolation. Previous observational studies suggest relationships between these and a range of health outcomes.<br><br>"However, establishing whether these associations reflect potentially causal relationships, and the direction of causation, can be difficult. Most previous studies have been unable to address this because they may be subject to residual confounding of the relationships between loneliness/social isolation and health outcomes, and reverse causation [3].<br><br>One approach that can overcome the issues of confounding and reverse causation, is Mendelian Randomisation (MR)…"<br><br>We used triangulation of observational sibling control and MR in this study.
3	**Objectives**	State specific objectives clearly, including pre-specified causal hypotheses (if any). State that MR is a method that, under specific assumptions, intends to estimate causal effects	5	"We employed a triangulation approach [33,34] that consisted of three parts: 1) observational analyses to establish whether associations exist between loneliness or social isolation and the health outcomes, adjusting for important potential confounders; 2) sibling control analyses to facilitate control for familial confounding (e.g., parental socioeconomic status) and shared genetic predispositions; and 3) MR analyses to estimate effects, reducing bias from confounding and reverse causation. These analyses, in combination, provide evidence as to whether the associations reflect potential causal effects."<br><br>Assumptions are discussed specifically in the methods (pg 23)
	**METHODS**			
4	**Study design and data sources**	Present key elements of the study design early in the article. Consider including a table listing sources of data		

	for all phases of the study. For each data source contributing to the analysis, describe the following:		
a)	Setting: Describe the study design and the underlying population, if possible. Describe the setting, locations, and relevant dates, including periods of recruitment, exposure, follow-up, and data collection, when available.	19-20	We used GWAS data and data from UK Biobank.<br>"*UK Biobank:* For conventional multivariable observational, sibling control, and 1SMR analyses we used phenotypic and genetic data from the UK Biobank, a large population-based prospective cohort of around 500,000 participants aged between 38 and 73 years and living in the UK, recruited between 2006 and 2010"<br><br>"We used publicly available GWAS summary statistics (N= 17,526 to 2,083,151) for our 2SMR analyses and for our exposures of loneliness and social isolation in 1SMR analyses (see Supplementary Table S1). However, we conducted our own GWAS of social isolation in UK Biobank (see Supplementary Methods Section 3, Supplementary Figure S1 and Supplementary Table S3)."<br><br>Full details are available in the Supplementary Methods.
b)	Participants: Give the eligibility criteria, and the sources and methods of selection of participants. Report the sample size, and whether any power or sample size calculations were carried out prior to the main analysis		See Supplementary Tables S1 and S2.<br><br>Power calculations were pre-registered, see https://doi.org/10.17605/OSF.IO/GWPNY.
c)	Describe measurement, quality control and selection of genetic variants	23-24	1SMR: "Due to the exposure GWAS completely (social isolation) or partially (loneliness) being conducted in UK Biobank, and to thus avoid sample overlap which can bias estimates due to overfitting [73], we used a split sample approach [74]. For this approach we split the sample into two random halves and in each half, we conducted a GWAS on social isolation and loneliness in UK Biobank (see Supplementary Methods Section 3). We used this GWAS data to create genetic scores in the other half of the sample, by using genetic variants and their weights with a p-value threshold of $1x10^{-05}$. We then used these to conduct our 1SMR analyses in each half of the sample. The results from the analyses in each half were then combined in a meta-analysis. Further details of this approach and sensitivity analyses conducted can be found in Supplementary Methods Section 7."<br><br>2SMR: "We used summary data (including weights) of genetic variant-exposure genome-wide associations from published GWAS as described in Supplementary Table S1 as genetic instruments for the exposures in our analyses. These were selected based on a p-value threshold of $5x10^{-08}$ for all analyses, except where suicide attempt ($1x10^{-06}$) and anxiety ($1x10^{-05}$) were the exposures. The steps taken to prepare these data for analyses are described in Supplementary Methods Section 8.

		d)	For each exposure, outcome, and other relevant variables, describe methods of assessment and diagnostic criteria for diseases		See Supplementary Tables S1 and S2.
		e)	Provide details of ethics committee approval and participant informed consent, if relevant	19	"UK Biobank received ethics approval from the UK National Health Service Research Ethics Committee (REC reference for UK Biobank is 11/NW/0382) and all participants gave informed consent to the use of their anonymised data and samples for any health-related research and for UK Biobank to access their health-related records."
5	**Assumptions**		Explicitly state the three core IV assumptions for the main analysis (relevance, independence and exclusion restriction) as well assumptions for any additional or sensitivity analysis	23 - 24	"There are three core assumptions underlying MR:<br><br>1. The genetic instruments are robustly associated with the exposure of interest;<br>2. There is no confounding of the genetic instrument-outcome relationship;<br>3. The genetic instruments are only associated with the outcome via the exposure, and there are no direct effects between the genetic IV and the outcome"<br><br>We included a range of sensitivity analyses: "The steps taken to prepare these data for analyses are described in Supplementary Methods Section 8. For the main analyses we used the inverse-variance weighted (IVW) method [75]. The sensitivity analyses assess various assumptions and potential biases in the MR approach. Inconsistent results may suggest bias from MR assumptions being violated. The sensitivity analyses included were MR-Egger [52], weighted median [76], MR using robust adjusted profile score (MR-RAPS) [77], MR Pleiotropy RESidual Sum and Outlier (MR-PRESSO) [78], MR lap [79] and MR-Causal Analysis Using Summary Effect estimates (CAUSE) [53]. We also conducted Steiger filtering [54] to further infer the most likely direction of effect. Supplementary Methods Section 8 further describes these sensitivity analyses."
6	**Statistical methods: main analysis**		Describe statistical methods and statistics used		
		a)	Describe how quantitative variables were handled in the analyses (i.e., scale, units, model)	21	"We conducted linear regressions (for continuous outcomes), logistic regressions (for binary outcomes) or Cox proportional hazard models (time-to-event outcomes) (i.e., all-cause mortality and incident CAD, heart failure and stroke). Supplementary Methods Section 4 describes how we applied the Cox proportional hazards models in this study."<br><br>Also see Supplementary Tables S1 and S2 for specific methods. Scales are also described in Figure legends.

		b)	Describe how genetic variants were handled in the analyses and, if applicable, how their weights were selected	23-24	1SMR: "Due to the exposure GWAS completely (social isolation) or partially (loneliness) being conducted in UK Biobank, and to thus avoid sample overlap which can bias estimates due to overfitting [73], we used a split sample approach [74]. For this approach we split the sample into two random halves and in each half, we conducted a GWAS on social isolation and loneliness in UK Biobank (see Supplementary Methods Section 3). We used this GWAS data to create genetic scores in the other half of the sample, by using genetic variants and their weights with a p-value threshold of $1x10^{-05}$. We then used these to conduct our 1SMR analyses in each half of the sample. The results from the analyses in each half were then combined in a meta-analysis. Further details of this approach and sensitivity analyses conducted can be found in Supplementary Methods Section 7."<br><br>2SMR: "We used summary data (including weights) of genetic variant-exposure genome-wide associations from published GWAS as described in Supplementary Table S1 as genetic instruments for the exposures in our analyses. These were selected based on a p-value threshold of $5x10^{-08}$ for all analyses, except where suicide attempt ($1x10^{-06}$) and anxiety ($1x10^{-05}$) were the exposures. The steps taken to prepare these data for analyses are described in Supplementary Methods Section 8.
		c)	Describe the MR estimator (e.g. two-stage least squares, Wald ratio) and related statistics. Detail the included covariates and, in case of two-sample MR, whether the same covariate set was used for adjustment in the two samples	23	"In our 1SMR analyses we adjusted for age, sex, the first 20 principal components (PCs) from PC analysis of genotype data, assessment centre and genotyping chip (Supplementary Table S4)."
		d)	Explain how missing data were addressed	6 and 20	We used data that were available for the relevant measures.<br><br>"We used this to investigate the relationships between both loneliness and social isolation and a range of physical, general, and mental health outcomes, with the caveat that some outcomes were not available for either the 1SMR approach (as individual data was needed) or the 2SMR approach (as GWAS data was needed)."<br><br>"The number of UK Biobank participants for our main analyses ranged from 77,577 to 414,432 for observational analyses, 8,004 to 40,440 for sibling control analyses and 106,828 to 333,358 for 1SMR. The difference in sample sizes was due to the data available for each measure."
		e)	If applicable, indicate how multiple testing was addressed	NA	NA
7	**Assessment of assumptions**		Describe any methods or prior knowledge used to assess the assumptions or justify their validity	24	1SMR: "Further details of this approach and sensitivity analyses conducted can be found in Supplementary Methods Section 7."

				2SMR: "We used summary data (including weights) of genetic variant-exposure genome-wide associations from published GWAS as described in Supplementary Table S1 as genetic instruments for the exposures in our analyses. These were selected based on a p-value threshold of $5x10^{-08}$ for all analyses, except where suicide attempt ($1x10^{-06}$) and anxiety ($1x10^{-05}$) were the exposures. The steps taken to prepare these data for analyses are described in Supplementary Methods Section 8."
8	**Sensitivity analyses and additional analyses**	Describe any sensitivity analyses or additional analyses performed (e.g. comparison of effect estimates from different approaches, independent replication, bias analytic techniques, validation of instruments, simulations)	24	1SMR: "Further details of this approach and sensitivity analyses conducted can be found in Supplementary Methods Section 7."<br><br>2SMR: "The steps taken to prepare these data for analyses are described in Supplementary Methods Section 8. For the main analyses we used the inverse-variance weighted (IVW) method [75]. The sensitivity analyses assess various assumptions and potential biases in the MR approach. Inconsistent results may suggest bias from MR assumptions being violated. The sensitivity analyses included were MR-Egger [52], weighted median [76], MR using robust adjusted profile score (MR-RAPS) [77], MR Pleiotropy RESidual Sum and Outlier (MR-PRESSO) [78], MR lap [79] and MR-Causal Analysis Using Summary Effect estimates (CAUSE) [53]. We also conducted Steiger filtering [54] to further infer the most likely direction of effect. Supplementary Methods Section 8 further describes these sensitivity analyses".
9	**Software and pre-registration**			
	a)	Name statistical software and package(s), including version and settings used	21-24	"All analyses were conducted in R version 4.1.0 [66].<br><br>"For 1SMR analyses we used the Applied Econometrics with R (AER) package in R [71] to conduct two-stage least squares regressions"<br><br>"We conducted bidirectional 2SMR analyses (i.e., with loneliness/social isolation as exposures and outcomes) using the 'TwoSampleMR' R package [35]"<br><br>Additional packages are described in relevant sections in the supplementary methods.
	b)	State whether the study protocol and details were pre-registered (as well as when and where)	19	"The analysis plan is pre-registered on the Open Science Framework (https://doi.org/10.17605/OSF.IO/GWPNY; pre-registered 18/12/2023). The analysis plan is located under the 'Files' section. Deviations from this analysis plan are outlined in Supplementary Methods Section 1."
	**RESULTS**			
10	**Descriptive data**			

		a)	Report the numbers of individuals at each stage of included studies and reasons for exclusion. Consider use of a flow diagram	20	“The number of UK Biobank participants for our main analyses ranged from 77,577 to 414,432 for observational analyses, 8,004 to 40,440 for sibling control analyses and 106,828 to 333,358 for 1SMR. The difference in sample sizes was due to the data available for each measure.”
		b)	Report summary statistics for phenotypic exposure(s), outcome(s), and other relevant variables (e.g. means, SDs, proportions)	39	See Table 1
		c)	If the data sources include meta-analyses of previous studies, provide the assessments of heterogeneity across these studies	NA	NA
		d)	For two-sample MR:<br>i. Provide justification of the similarity of the genetic variant-exposure associations between the exposure and outcome samples<br>ii. Provide information on the number of individuals who overlap between the exposure and outcome studies	SI pg7	“MR lap accounts for any sample overlap and reduces bias which may occur when using overlapping samples in the exposure and outcome. MR CAUSE can model both uncorrelated and correlated horizontal pleiotropy, avoiding false positives that may result from correlated pleiotropy.”<br>Also see Table S1 for GWAS specific details.
11	**Main results**				
		a)	Report the associations between genetic variant and exposure, and between genetic variant and outcome, preferably on an interpretable scale	NA	NA
		b)	Report MR estimates of the relationship between exposure and outcome, and the measures of uncertainty from the MR analysis, on an interpretable scale, such as odds ratio or relative risk per SD difference	SI pg 63, pg 91	See Table S13 and S14
		c)	If relevant, consider translating estimates of relative risk into absolute risk for a meaningful time period	NA	NA
		d)	Consider plots to visualize results (e.g. forest plot, scatterplot of associations between genetic variants and outcome versus between genetic variants and exposure)	pg 41-44	Figures 1 to 6
12	**Assessment of assumptions**				
		a)	Report the assessment of the validity of the assumptions	SI pg 63, pg 91	See Table S13 and S14

		b)	Report any additional statistics (e.g., assessments of heterogeneity across genetic variants, such as I^2, Q statistic or E-value)	SI pg 63, pg 91	See Table S13 and S14
13	**Sensitivity analyses and additional analyses**				
		a)	Report any sensitivity analyses to assess the robustness of the main results to violations of the assumptions	SI pg 63, pg 91	See Table S13 and S14
		b)	Report results from other sensitivity analyses or additional analyses	SI pg 63, pg 91	See Table S13 and S14
		c)	Report any assessment of direction of causal relationship (e.g., bidirectional MR)	SI pg 91	See Table S14
		d)	When relevant, report and compare with estimates from non-MR analyses	Pg 6 to 12	Paper presents a triangulation approach
		e)	Consider additional plots to visualize results (e.g., leave-one-out analyses)	NA	NA
	**DISCUSSION**				
14	**Key results**		Summarize key results with reference to study objectives	13	"Using a triangulation approach, our results provide consistent evidence across analyses that loneliness may causally negatively impact mental health and wellbeing outcomes, and that social isolation may causally negatively impact wellbeing outcomes. This is in line with previous studies demonstrating observational associations between loneliness, social isolation and poorer mental health [1,7,8]. Our findings suggest that effects on mental health and wellbeing may be stronger for loneliness and that loneliness could also be influencing the effect of social isolation on positive affect. We also found consistent evidence across analyses that being lonely may causally negatively impact some aspects of general health (i.e., increasing the risk of multimorbidity and decreasing QALYs), in line with previous studies [1]. However, in contrast to previous observational studies [2,14] we did not find consistent evidence across analyses of effects of social isolation on general health, or of either loneliness or social isolation on specific physical health outcomes."
15	**Limitations**		Discuss limitations of the study, taking into account the validity of the IV assumptions, other sources of potential bias, and imprecision. Discuss both direction and magnitude of any potential bias and any efforts to address them	15-17	"The main strength of our study is the use of a triangulation approach. The different methods we used have their own strengths and biases, but by interpreting the results together we have greater confidence in our findings. Another strength is the range of outcomes we explored which incorporate both self-reported and linked NHS electronic care record data, covering different aspects of general, physical and mental health and wellbeing in an in-depth manner. We also included an extensive number of sensitivity

analyses across our approaches which address different biases and assumptions, resulting in more robust conclusions. Finally, we used data from large and recent GWAS and UK Biobank, a large cohort study with rich phenotypic data.

There are several limitations to the current study that should be considered when interpreting our findings. First, there were several analyses, particularly where social isolation was the exposure, where our MR estimates had wide confidence intervals meaning that statistical power may have been lacking to detect effects. Second, our measure of loneliness in UK Biobank was a binary response to a single question asking whether the respondent was lonely, whereas other studies have used composite measures of loneliness. Loneliness is likely to be captured better using such validated composite measures that include different aspects of loneliness. The single item measure we have used could result in weaker associations in this study. Similarly, our social isolation measure did not capture social interactions outside of the household, friends and family, potentially missing key sources of social interaction. Furthermore, our measures of both loneliness and social isolation were assessed at a single time point during middle-late adulthood. Both loneliness and social isolation are likely to change across the life course, and important time periods for loneliness and social isolation e.g., childhood, adolescence and young adulthood are not captured in this study. It may be that effects on the health outcomes studied here vary across different life stages and therefore conclusions about the impact of loneliness and social isolation in earlier life are limited. However, even a single measurement of loneliness is likely to reflect chronic loneliness in some individuals. In addition, by including only a single timepoint we were able to able to examine outcomes that temporally occurred after the exposure was measured. We did include MR analyses which should provide estimates of effects over the lifetime, but these results would be strengthened by the inclusion of analyses in age groups not covered in UK Biobank. Third, whilst we accounted for several important potential confounders in our observational and sibling control analyses, there may be factors that we could not account for. Fourth, results from our sibling analyses may not be generalisable because it required there to be at least two siblings from the same family participating in UK Biobank. In terms of representation, UK Biobank participants are generally healthier and less socioeconomically deprived than the general population [60] and our MR analyses were restricted to individuals of European ancestry. Therefore, our results may not be generalisable outside of those populations. In addition, this may mean that there are fewer cases in terms of the health outcomes examined, continuous measures may be skewed towards ‘healthier’ ranges, and rates of loneliness and social isolation may be lower than in the general population. Individuals with poorer health or who are more lonely or socially isolated may have been less likely to participate in UK Biobank, introducing selection bias in our analyses which could bias associations. Therefore, caution should be taken when interpreting results from analyses based solely on UK Biobank data. Fifth, in our two-sample MR analyses where we included binary exposures, there are limitations to interpreting these effects where these exposures may reflect dichotomisation of an underlying continuous trait e.g., a diagnosis of depression reflects a clinical cut-off of an underlying continuous distribution of depressive symptoms [61]. The

				main limitation of including binary exposures is that the effect estimate may not be particularly interpretable, Therefore, results from MR analyses with binary exposures in this study should be considered in terms of direction of effect as opposed to overly focusing on the magnitude of the effect. Sixth, loneliness and social isolation are unlikely to be directly caused by the genetic variants used as instruments in this study (i.e., they are biologically distal and the pathways from genetic variants to these exposures are likely to be complex). MR analyses are likely to be more reliable when exposures are more directly related to the genetic variants included as instruments, for example, when using biological exposures such as blood-based measures 16. Additionally, biologically distal exposures may increase the chances of there being horizontal pleiotropy. Although we conducted a range of sensitivity analyses to address this, it may be that pleiotropic pathways do still exist. This should be considered when interpreting the MR results and the MR results should also be interpreted alongside the other analyses within the triangulation framework."
16	**Interpretation**			
		a)	Meaning: Give a cautious overall interpretation of results in the context of their limitations and in comparison with other studies	18	"Our study used a combination of approaches, including some that were novel or had limited prior applications in this field (i.e., sibling control and MR analyses), to investigate whether loneliness and social isolation may influence a variety of general, physical and mental health and wellbeing outcomes. Overall, our triangulation study provided robust evidence suggesting effects of loneliness and social isolation on poorer mental health and wellbeing and of loneliness on poorer general health. Taken together, these findings suggest that loneliness, and potentially social isolation, are still important public health concerns, especially with respect to mental health and general health. Interventions targeting loneliness and social isolation may prove to be effective strategies for improving a range of mental health, wellbeing and general health outcomes."
		b)	Mechanism: Discuss underlying biological mechanisms that could drive a potential causal relationship between the investigated exposure and the outcome, and whether the gene-environment equivalence assumption is reasonable. Use causal language carefully, clarifying that IV estimates may provide causal effects only under certain assumptions	17	"Given the findings in this study there are some useful directions that future research could take. It is important to better understand the pathways from loneliness/social isolation to mental health and wellbeing outcomes to target effective interventions to improve these outcomes. Examining the impact of loneliness and social isolation across the life course would provide additional insight, but this was not possible in the current study given the data available. Longitudinal studies (including chronic loneliness and social isolation) would be useful to provide a more comprehensive understanding of the impact of these exposures on health outcomes. In addition, examining relationships with other physical outcomes would also be helpful. Finally, further analyses that would complement this triangulation approach would be useful. For example, within-family MR would be helpful to better account for confounding due to factors such as population stratification and dynastic effects. We did not include this approach here as using the sibling data in UK Biobank would result in underpowered analyses [62], due to the small sample sizes we would have when combined with a split sample approach (similar to our one-sample approach, a split sample approach would be required given the sample overlap in the GWAS and UK Biobank). Further analyses using larger samples with data from mother, father, and child

				trios or sibling pairs may provide greater statistical power; however, the availability of such data to support this type of analysis remains limited.”
		c) Clinical relevance: Discuss whether the results have clinical or public policy relevance, and to what extent they inform effect sizes of possible interventions	13	“We found comparable effect sizes with previously published observational effects (e.g., for depression and death) [7,55]. Our results therefore suggest potentially large negative effects of loneliness on mental health. We also found evidence of effects in the other direction (i.e., poorer mental health and wellbeing causing loneliness or greater social isolation). This suggests that the relationships between loneliness and social isolation and mental health and wellbeing may exacerbate each other..”
17	**Generalizability**	Discuss the generalizability of the study results (a) to other populations, (b) across other exposure periods/timings, and (c) across other levels of exposure	16	“Fourth, results from our sibling analyses may not be generalisable because it required there to be at least two siblings from the same family participating in UK Biobank. In terms of representation, UK Biobank participants are generally healthier and less socioeconomically deprived than the general population [60] and our MR analyses were restricted to individuals of European ancestry. Therefore, our results may not be generalisable outside of those populations.”
	**OTHER INFORMATION**			
18	**Funding**	Describe sources of funding and the role of funders in the present study and, if applicable, sources of funding for the databases and original study or studies on which the present study is based	37	“This work was supported by Nesta. Some of the co-authors (DDH, PB, LTH, LBB) work at Nesta and were involved in the design of the study, analysis, and write up. This work was also supported in part by the UK Medical Research Council Integrative Epidemiology Unit at the University of Bristol (MC_UU_00032/1 and MC_UU_00032/7). REW is funded by a postdoctoral fellowship from the South-Eastern Norway Regional Health Authority (2020024). JT and MPVDW are funded by the European Union (ERC, UNRAVEL-CAUSALITY, project nr. 101076686). Views and opinions expressed are however those of the author(s) only and do not necessarily reflect those of the European Union or the European Research Council. Neither the European Union nor the granting authority can be held responsible for them. PD received support from the National Institute for Health and Care Research Applied Research Collaboration for Oxford and the Thames Valley (NIHR ARC OxTV) through a Dementia Capacity Building Post-Doctoral Training Scheme (DEM-COMM). The views expressed are those of the authors and not necessarily those of the funders, NHS or Department of Health and Social Care. RCR is supported by Cancer Research UK (grant number: C18281/A29019).”
19	**Data and data sharing**	Provide the data used to perform all analyses or report where and how the data can be accessed, and reference these sources in the article. Provide the statistical code needed to reproduce the results in the article, or report whether the code is publicly accessible and if so, where	25-26	“Data availability<br>The UK Biobank data are available under restricted access for approved researchers, access can be obtained through a procedure described at http://www.ukbiobank.ac.uk/using-the-resource/. Access details for GWAS data used in this study are outlined below:

Social isolation: GWAS summary statistics generated as part of this study are available from the University of Bristol's Research Data Repository (http://data.bris.ac.uk/data/), at: https://data.bris.ac.uk/data/dataset/39m1awtj2tjtu2ugpbq5lg12hx.

Loneliness: GWAS summary statistics excluding 23andMe can be downloaded from https://t.co/ARgS84uwKl. Data for the top 10,000 genetic variants including 23andMe are available from the authors at request.

Coronary artery disease (CAD): GWAS summary statistics are available here: http://ftp.ebi.ac.uk/pub/databases/gwas/summary_statistics/GCST90132001-GCST90133000/GCST90132314/ with accession GCST90132314

Heart failure: GWAS summary statistics are available here: https://cvd.hugeamp.org/dinspector.html?dataset=GWAS_HERMES_eu.

Stroke: Details on the GWAS can be found here: https://www.megastroke.org/. For the European subset we contacted the authors of the paper.

Systolic blood pressure: GWAS summary statistics are available here: https://www.ebi.ac.uk/gwas/publications/30224653 with accession GCST006624.

Type 2 diabetes: GWAS summary statistics are available on request from dbGaP (

T2D: Phs001672; Pha004945; T2D.EUR.MVP_Penn_DIAMANTE_Malmo.NatGen2020)

Suicide attempts: The GWAS summary statistics can be requested here: https://docs.google.com/forms/d/e/1FAIpQLSc4hGOJ181WGfP3yTjMxUmdf4d6hK0dkXSwRO0ivcPdZIOslg/viewform.

Depression: The GWAS summary statistics are available to download from the Psychiatric Genomics Consortium (PGC) (https://figshare.com/articles/dataset/mdd2018/14672085) after agreeing to abide by their data access conditions.

Anxiety: The GWAS summary statistics are available to download from the PGC (https://pgc.unc.edu/for-researchers/download-results/?choice=Other+GWAS+DataAnxiety+Neuro+Genetics+Study+%2528ANGST%2529) after agreeing to abide by their data access conditions.

				Wellbeing: The GWAS summary statistics excluding 23andMe are available to download from: https://www.ebi.ac.uk/gwas/publications/30643256.<br><br>Code availability<br><br>Analysis code is available from the University of Bristol's Research Data Repository (http://data.bris.ac.uk/data/), at: https://data.bris.ac.uk/data/dataset/39m1awtj2tjtu2ugpbq5lg12hx [81].
20	**Conflicts of Interest**	All authors should declare all potential conflicts of interest	38	"Nesta funded this project and were involved in the conceptualisation, analysis and preparation of the manuscript, including staff as co-authors on the paper. The authors declare no other competing interests."

This checklist is copyrighted by the Equator Network under the Creative Commons Attribution 3.0 Unported (CC BY 3.0) license (https://creativecommons.org/licenses/by/3.0/deed.en). No changes have been made to the checklist. This checklist is available from: https://www.strobe-mr.org/ [39,40].

References

1. Bycroft, C. *et al.* The UK Biobank resource with deep phenotyping and genomic data. *Nature* **562**, 203–209 (2018).
2. Mitchell, R. *et al.* UK Biobank Genetic Data: MRC-IEU Quality Control, Version 2. (2019) doi:https://doi.org/10.5523/bris.1ovaau5sxunp2cv8rcy88688v.
3. Birney, E., Inouye, M., Raff, J., Rutherford, A. & Scally, A. The language of race, ethnicity, and ancestry in human genetic research. *arXiv* https://doi.org/https://doi.org/10.48550/arXiv.2106.10041 (2021) doi:https://doi.org/10.48550/arXiv.2106.10041.
4. Manichaikul, A. *et al.* Robust relationship inference in genome-wide association studies. *Bioinformatics* **26**, 2867–2873 (2010).
5. Brumpton, B. *et al.* Avoiding dynastic, assortative mating, and population stratification biases in Mendelian randomization through within-family analyses. *Nature Communications* **11**, 3519 (2020).
6. Bautista, L. E., Smeeth, L., Hingorani, A. D. & Casas, J. P. Estimation of Bias in Nongenetic Observational Studies Using "Mendelian Triangulation". *Annals of Epidemiology* **16**, 675–680 (2006).
7. Balduzzi, S., Rücker, G. & Schwarzer, G. How to perform a meta-analysis with R: a practical tutorial. *Evid Based Mental Health* **22**, 153–160 (2019).
8. Hemani, G. *et al.* MR-Base: a platform for systematic causal inference across the phenome using billions of genetic associations. *bioRxiv* 078972 (2016) doi:10.1101/078972.
9. Minelli, C. *et al.* The use of two-sample methods for Mendelian randomization analyses on single large datasets. *International Journal of Epidemiology* **50**, 1651–1659 (2021).
10. Machiela, M. J. & Chanock, S. J. LDlink: A web-based application for exploring population-specific haplotype structure and linking correlated alleles of possible functional variants. *Bioinformatics* **31**, 3555–3557 (2015).
11. Bowden, J., Davey Smith, G. & Burgess, S. Mendelian randomization with invalid instruments: Effect estimation and bias detection through Egger regression. *International Journal of Epidemiology* **44**, 512–525 (2015).
12. Bowden, J., Davey Smith, G., Haycock, P. C. & Burgess, S. Consistent Estimation in Mendelian Randomization with Some Invalid Instruments Using a Weighted Median Estimator. *Genetic Epidemiology* **40**, 304–314 (2016).
13. Zhao, Q., Wang, J., Hemani, G., Bowden, J. & Small, D. S. Statistical inference in two-sample summary-data Mendelian randomization using robust adjusted profile score. *Annals of Statistics* **48**, 1742–1769 (2020).

14. Verbanck, M., Chen, C. Y., Neale, B. & Do, R. Detection of widespread horizontal pleiotropy in causal relationships inferred from Mendelian randomization between complex traits and diseases. *Nature Genetics* **50**, 693–698 (2018).

15. Mounier, N. & Kutalik, Z. Bias correction for inverse variance weighting Mendelian randomization. *Genetic Epidemiology* **47**, 314–331 (2023).

16. Morrison, J., Knoblauch, N., Marcus, J. H., Stephens, M. & He, X. Mendelian randomization accounting for correlated and uncorrelated pleiotropic effects using genome-wide summary statistics. *Nat Genet* **52**, 740–747 (2020).

17. Lawlor, D. A., Harbord, R. M., Sterne, J. A. C., Timpson, N. & Davey Smith, G. Mendelian randomization: Using genes as instruments for making causal inferences in epidemiology. *Statistics in Medicine* **27**, 1133–1163 (2008).

18. Hemani, G., Tilling, K. & Davey Smith, G. Orienting the causal relationship between imprecisely measured traits using GWAS summary data. *PLOS Genetics* **13**, e1007081 (2017).

19. Sanderson, E., Spiller, W. & Bowden, J. Testing and correcting for weak and pleiotropic instruments in two-sample multivariable Mendelian randomization. *Statistics in Medicine* **40**, 5434–5452 (2021).

20. Abdellaoui, A. *et al.* Phenome-wide investigation of health outcomes associated with genetic predisposition to loneliness. *Human Molecular Genetics* **28**, 3853–3865 (2019).

21. Aragam, K. G. *et al.* Discovery and systematic characterization of risk variants and genes for coronary artery disease in over a million participants. *Nat Genet* **54**, 1803–1815 (2022).

22. Shah, S. *et al.* Genome-wide association and Mendelian randomisation analysis provide insights into the pathogenesis of heart failure. *Nat Commun* **11**, 163 (2020).

23. Mishra, A. *et al.* Stroke genetics informs drug discovery and risk prediction across ancestries. *Nature* **611**, 115–123 (2022).

24. Evangelou, E. *et al.* Genetic analysis of over 1 million people identifies 535 new loci associated with blood pressure traits. *Nat Genet* **50**, 1412–1425 (2018).

25. Vujkovic, M. *et al.* Discovery of 318 new risk loci for type 2 diabetes and related vascular outcomes among 1.4 million participants in a multi-ancestry meta-analysis. *Nature Genetics 2020 52:7* **52**, 680–691 (2020).

26. Mullins, N. *et al.* Dissecting the Shared Genetic Architecture of Suicide Attempt, Psychiatric Disorders, and Known Risk Factors. *Biol Psychiatry* **91**, 313–327 (2022).

27. Wray, N. R. *et al.* Genome-wide association analyses identify 44 risk variants and refine the genetic architecture of major depression. *Nature Genetics* **50**, 668–681 (2018).

28. Otowa, T. *et al.* Meta-analysis of genome-wide association studies of anxiety disorders. *Molecular Psychiatry 2016 21:10* **21**, 1391–1399 (2016).

29. Baselmans, B. M. L. *et al.* A Genetic Investigation of the Well-Being Spectrum. *Behavior Genetics* **49**, 286–297 (2019).

30. Hazewinkel, A.-D., Richmond, R. C., Wade, K. H. & Dixon, P. Mendelian randomization analysis of the causal impact of body mass index and waist-hip ratio on rates of hospital admission. *Economics & Human Biology* **44**, 101088 (2022).

31. Harrison, S. *et al.* Long-term cost-effectiveness of interventions for obesity: A mendelian randomisation study. *PLOS Medicine* **18**, e1003725 (2021).

32. Sullivan, P. W., Slejko, J. F., Sculpher, M. J. & Ghushchyan, V. Catalogue of EQ-5D Scores for the United Kingdom. *Med Decis Making* **31**, 800–804 (2011).

33. Dolan, P. Modeling Valuations for EuroQol Health States. *Medical Care* **35**, 1095 (1997).

34. North, T.-L. *et al.* Educational inequality in multimorbidity: causality and causal pathways. A mendelian randomisation study in UK Biobank. *BMC Public Health* **23**, 1644 (2023).

35. Patel, R. S. *et al.* Reproducible disease phenotyping at scale: Example of coronary artery disease in UK Biobank. *PLOS ONE* **17**, e0264828 (2022).

36. Eastwood, S. V. *et al.* Algorithms for the Capture and Adjudication of Prevalent and Incident Diabetes in UK Biobank. *PLOS ONE* **11**, e0162388 (2016).

37. Okbay, A. *et al.* Genome-wide association study identifies 74 loci associated with educational attainment. *Nature* **533**, 539–542 (2016).

38. Soares, A. L. G. *et al.* Sex differences in the association between childhood maltreatment and cardiovascular disease in the UK Biobank. *Heart* **106**, 1310–1316 (2020).

39. Strengthening the reporting of observational studies in epidemiology using mendelian randomisation (STROBE-MR): explanation and elaboration | The BMJ. https://www.bmj.com/content/375/bmj.n2233.full.

40. Skrivankova, V. W. *et al.* Strengthening the Reporting of Observational Studies in Epidemiology Using Mendelian Randomization: The STROBE-MR Statement. *JAMA* **326**, 1614–1621 (2021).
