# Supplementary material for: Investigating relationships between loneliness, social isolation and health
Source: Nat Commun. 2026 Jul 15;17:5840. doi: 10.1038/s41467-026-74758-7 (PMC13373220; doi:10.1038/s41467-026-74758-7)
Supplement: Supplementary file 2 — Reporting Summary [file 41467_2026_74758_MOESM2_ESM.pdf]

nature portfolio

Corresponding author(s): Zoe E Reed

Last updated by author(s): Jun 5, 2026

Reporting Summary

Nature Portfolio wishes to improve the reproducibility of the work that we publish. This form provides structure for consistency and transparency in reporting. For further information on Nature Portfolio policies, see our Editorial Policies and the Editorial Policy Checklist.

Statistics

For all statistical analyses, confirm that the following items are present in the figure legend, table legend, main text, or Methods section.

n/a	Confirmed	
☐	☒	The exact sample size (*n*) for each experimental group/condition, given as a discrete number and unit of measurement
☐	☒	A statement on whether measurements were taken from distinct samples or whether the same sample was measured repeatedly
☐	☒	The statistical test(s) used AND whether they are one- or two-sided<br>*Only common tests should be described solely by name; describe more complex techniques in the Methods section.*
☐	☒	A description of all covariates tested
☐	☒	A description of any assumptions or corrections, such as tests of normality and adjustment for multiple comparisons
☐	☒	A full description of the statistical parameters including central tendency (e.g. means) or other basic estimates (e.g. regression coefficient) AND variation (e.g. standard deviation) or associated estimates of uncertainty (e.g. confidence intervals)
☐	☒	For null hypothesis testing, the test statistic (e.g. *F*, *t*, *r*) with confidence intervals, effect sizes, degrees of freedom and *P* value noted<br>*Give P values as exact values whenever suitable.*
☒	☐	For Bayesian analysis, information on the choice of priors and Markov chain Monte Carlo settings
☒	☐	For hierarchical and complex designs, identification of the appropriate level for tests and full reporting of outcomes
☐	☒	Estimates of effect sizes (e.g. Cohen's *d*, Pearson's *r*), indicating how they were calculated

Our web collection on statistics for biologists contains articles on many of the points above.

Software and code

Policy information about availability of computer code

Data collection	Analysis code is available from the University of Bristol's Research Data Repository (http://data.bris.ac.uk/data/), at: https://data.bris.ac.uk/data/dataset/39m1awtj2tjtu2ugpbq5lg12hx.
Data analysis	Analysis code is available from the University of Bristol's Research Data Repository (http://data.bris.ac.uk/data/), at: at: https://data.bris.ac.uk/data/dataset/39m1awtj2tjtu2ugpbq5lg12hx. All analyses were conducted in R version 4.1.0.

For manuscripts utilizing custom algorithms or software that are central to the research but not yet described in published literature, software must be made available to editors and reviewers. We strongly encourage code deposition in a community repository (e.g. GitHub). See the Nature Portfolio guidelines for submitting code & software for further information.

Data

Policy information about availability of data

All manuscripts must include a data availability statement. This statement should provide the following information, where applicable:

- Accession codes, unique identifiers, or web links for publicly available datasets
- A description of any restrictions on data availability
- For clinical datasets or third party data, please ensure that the statement adheres to our policy

The UK Biobank data are available under restricted access for approved researchers, access can be obtained through a procedure described at http://www.ukbiobank.ac.uk/using-the-resource/. Access details for GWAS data used in this study are outlined below:

Social isolation: GWAS summary statistics generated as part of this study are available from the University of Bristol's Research Data Repository (http://data.bris.ac.uk/data/), at: https://data.bris.ac.uk/data/dataset/39m1awtj2tjtu2ugpbq5lg12hx80.

Loneliness: GWAS summary statistics excluding 23andMe can be downloaded from https://t.co/ARgS84uwKl. Data for the top 10,000 genetic variants including 23andMe are available from the authors at request.

Coronary artery disease (CAD): GWAS summary statistics are available here: http://ftp.ebi.ac.uk/pub/databases/gwas/summary_statistics/GCST90132001-GCST90133000/GCST90132314/ with accession GCST90132314

Heart failure: GWAS summary statistics are available here: https://cvd.hugeamp.org/dinspector.html?dataset=GWAS_HERMES_eu.

Stroke: Details on the GWAS can be found here: https://www.megastroke.org/. For the European subset we contacted the authors of the paper.

Systolic blood pressure: GWAS summary statistics are available here: https://www.ebi.ac.uk/gwas/publications/30224653 with accession GCST006624.

Type 2 diabetes: GWAS summary statistics are available on request from dbGaP (
T2D: Phs001672; Pha004945; T2D.EUR.MVP_Penn_DIAMANTE_Malmo.NatGen2020)

Suicide attempts: The GWAS summary statistics can be requested here: https://docs.google.com/forms/d/e/1FAIpQLSc4hGOJ181WGfP3yTjMxUmdf4d6hK0dkXSwRO0ivcPdZIOslg/viewform.

Depression: The GWAS summary statistics are available to download from the Psychiatric Genomics Consortium (PGC) (https://figshare.com/articles/dataset/mdd2018/14672085) after agreeing to abide by their data access conditions.

Anxiety: The GWAS summary statistics are available to download from the PGC (https://pgc.unc.edu/for-researchers/download-results/?choice=Other+GWAS+DataAnxiety+Neuro+Genetics+Study+%2528ANGST%2529) after agreeing to abide by their data access conditions.

Wellbeing: The GWAS summary statistics excluding 23andMe are available to download from: https://www.ebi.ac.uk/gwas/publications/30643256.

Research involving human participants, their data, or biological material

Policy information about studies with human participants or human data. See also policy information about sex, gender (identity/presentation), and sexual orientation and race, ethnicity and racism.

Reporting on sex and gender

In Table 1 we present the UK Biobank sample characteristics: Female = 54% (sex obtained from NHS records)

In all observational and sibling control analyses we adjusted for potential confounders with two levels of adjustment. In our first adjusted models we included potential demographic confounders (including sex).

In our 1SMR analyses we adjusted for age, sex ...

Reporting on race, ethnicity, or other socially relevant groupings

We include in Table 1 Ethnicity (self-reported responses):
White: 94%
Asian or Asian British (Indian, Pakistani, Bangladeshi): 2%
Black or Black British: 2%
Mixed or any other Asian background, or Chinese or other ethnic group: 2%

We adjust for this in observational and sibling control analyses

We also note the following in our supplementary information:
In addition, we restricted our sample for 1SMR analyses to include only individuals who self-reported as 'White' and 'British' and who had very similar genetic ancestry based on a principal components analysis of genotypes. Self-reported responses were from touchscreen questionnaire questions asking, 'What is your ethnic group?' (response options: White; Mixed; Asian or Asian British; Black or Black British, Chinese, Other ethnic group, Do not know; Prefer not to answer). If they selected 'White' then they were asked 'What is your ethnic background?' (response options: British; Irish; Any other white background; Prefer not to answer). We acknowledge that ethnicity is a complex social construct with different meanings across different contexts, and is distinct to, although often overlapping with, genetic ancestry (Birney et al., 2021). This was done to ensure that the UK Biobank sample was similar in ancestry to the GWAS samples to avoid any bias in the results due to potential differences in associations by ancestry.

Population characteristics

This information is detailed in Table 1

Recruitment

UK Biobank: For conventional multivariable observational, sibling control, and 1SMR analyses we used phenotypic and genetic data from the UK Biobank, a large population-based prospective cohort of around 500,000 participants aged between 38 and 73 years and living in the UK, recruited between 2006 and 2010 55.

Selection biases and generalisability are discussed in the limitations section

Ethics oversight

UK Biobank received ethics approval from the UK National Health Service Research Ethics Committee (REC reference for UK Biobank is 11/NW/0382)

Note that full information on the approval of the study protocol must also be provided in the manuscript.

Field-specific reporting

Please select the one below that is the best fit for your research. If you are not sure, read the appropriate sections before making your selection.

☒ Life sciences ☐ Behavioural & social sciences ☐ Ecological, evolutionary & environmental sciences

For a reference copy of the document with all sections, see nature.com/documents/nr-reporting-summary-flat.pdf

Life sciences study design

All studies must disclose on these points even when the disclosure is negative.

Sample size

Sample size was not determined as we included all participants with relevant data.

For conventional multivariable observational, sibling control, and 1SMR analyses we used phenotypic and genetic data from the UK Biobank, a large population-based prospective cohort of around 500,000 participants aged between 38 and 73 years and living in the UK, recruited between 2006 and 2010. The number of UK Biobank participants for our main analyses ranged from 77,577 to 414,432 for observational analyses, 8,004 to 40,440 for sibling control analyses and 106,828 to 333,358 for 1SMR. The difference in sample sizes was due to the data available for each measure.

We used publicly available GWAS summary statistics for our 2SMR analyses and for our exposures of loneliness and social isolation in 1SMR analyses (see Supplementary Table S1). This table includes sample sizes. We used the largest GWAS available for each measure.

Data exclusions

We excluded participants who withdrew their consent using the latest withdrawal lists for this project (project number: 81499).

In UK Biobank there were 488,377 participants with genetic data available. Details on the quality control and other processing steps prior to using these data are described in Supplementary Information Section 2. The number of UK Biobank participants for our main analyses ranged from 77,577 to 414,432 for observational analyses, 8,004 to 40,440 for sibling control analyses and 106,828 to 333,358 for 1SMR. The difference in sample sizes was due to the data available for each measure.

For 1SMR: In addition, we restricted our sample for 1SMR analyses to include only individuals who self-reported as 'White' and 'British' and who had very similar genetic ancestry based on a principal components analysis of genotypes.

For the Social isolation GWAS: We used BOLT-LMM software within the pipeline to conduct our GWAS, which used a linear mixed model accounting for relatedness and population stratification and restricted to individuals of 'European' ancestry.

Replication

In our triangulation approach we first conducted observational analyses to establish whether there was an association between loneliness or social isolation and a given health outcome. We then used sibling control and Mendelian Randomisation (MR) analyses to provide evidence of effects. Triangulation is particularly useful when different methods have different biases and assumptions. If results are consistent across the different methods, there is greater confidence in the results. Our triangulation approach was qualitative (i.e., assessing whether there was consistent evidence of effects). Therefore, we have not focused on significance thresholds and have not adjusted for multiple testing. We have instead considered evidence across the main analyses, sensitivity analyses and have also considered the precision of estimates when interpreting results.

Randomization

There was no randomisation in this study as we used a secondary data analysis approach drawn from an existing general population study. Covariates were included in the analyses to adjust for potential confounders.

Blinding

This is not relevant to this study which used a secondary data analysis approach.

Reporting for specific materials, systems and methods

We require information from authors about some types of materials, experimental systems and methods used in many studies. Here, indicate whether each material, system or method listed is relevant to your study. If you are not sure if a list item applies to your research, read the appropriate section before selecting a response.

Materials & experimental systems

n/a	Involved in the study	
☒	☐	Antibodies
☒	☐	Eukaryotic cell lines
☒	☐	Palaeontology and archaeology
☒	☐	Animals and other organisms
☒	☐	Clinical data
☒	☐	Dual use research of concern
☒	☐	Plants

Methods

n/a	Involved in the study	
☒	☐	ChIP-seq
☒	☐	Flow cytometry
☒	☐	MRI-based neuroimaging

Plants

Seed stocks	NA
Novel plant genotypes	NA
Authentication	NA
