# Supplementary material for: Investigating relationships between loneliness, social isolation and health
Source: Nat Commun. 2026 Jul 15;17:5840. doi: 10.1038/s41467-026-74758-7 (PMC13373220; doi:10.1038/s41467-026-74758-7)
Supplement: Supplementary file 3 — Transparent Peer Review file [file 41467_2026_74758_MOESM3_ESM.pdf]

nature portfolio

Peer Review File

Investigating relationships between loneliness, social isolation and health

Corresponding Author: Dr Zoe Reed

This file contains all reviewer reports in order by version, followed by all author rebuttals in order by version.

Version 0:

Reviewer comments:

Reviewer #1

(Remarks to the Author)
This is a study which utilized different designs to investigate the relation of loneliness and social isolation with different health outcomes. Whilst important, I feel that the novelty of the study is not very clearly delineated, especially there are already quite some Mendelian randomization studies conducted (amongst which also include UK Biobank). I am also concerned over the use of a social construct as an exposure in an MR. Please see below for my comments.

Major comments
I am personally less hesitant to consider social construct as a valid exposure for Mendelian randomziation design given these constructs are not as well defined and could be vulnerable to different degrees of horizontal pleiotropy compared to other biomarkers which are more likely to have a biological component, such as lipids and glucose. This somewhat echos with the point in the limitation (Line 431-434). This is particularly important especially when there are already quite some MRs being conducted in this area. Stronger justification would be helpful to highlight the novelty of the study.

Please explain in more detail why a new GWAS is needed for the social isolation instead of using a previously published one in UK Biobank (https://www.nature.com/articles/s41467-018-04930-1). I think a more useful approach is to use a family-MR design to address possible violations of independence assumption (https://www.nature.com/articles/s41467-020-17117-4).

The bi-directional analyses would be difficult to interpret, especially for composite outcomes such as QALYs, let alone the issue of using binary traits (https://pubmed.ncbi.nlm.nih.gov/30039250/).

Line 178: Please clarify the meaning of complexity of the model when discussing results from the Cox model.

Whilst I agree that we should not only rely on statistical significance to determine causation, some estimates were very imprecise, such as the MR estimate for death, making these estimates nearly impossible to make any valid inference.

I am not sure if correction for multiple testing is present. Please clarify.

Minor comments
Line 149-150: Beta, 95%CI for phenotypic correlation are the same.

Line 170: Not sure why a p value of 0.83 was mentioned

Can the authors add references to the 2nd and 3rd paragraph of the Discussion? This would help readers to understand their interpretation in the context of other evidence.

Why is mean difference presented for dichotomous outcomes (e.g. Table S12)?

Reviewer #2

(Remarks to the Author)

The authors used a triangulation approach combining observational analysis, sibling control design, and Mendelian Randomization to explore the relationship between loneliness and social isolation and outcomes related to physical health, mental health, wellbeing and general health. I do have several questions and suggestions.
Major:
1. The authors completed the STROBE-MR checklist and described the three core assumptions underlying MR in the manuscript. In the observational and sibling control analysis, the author identified multiple confounders. However, those confounders were not mentioned or adjusted in the MR part. Does this study design violate the core assumptions of Mendelian randomization?
2. In the MR analysis part, the authors calculated F-statistics to estimate the strength of the instruments. This is a normal step for MR. However, I can not find any threshold that was determined and used. I think the threshold is necessary. What's more, some of the F-statistics shown in Table S13 were as low as 3.49. Do these IVs really meet the analysis requirements?
3. This study conducted a large-scale Mendelian randomization analysis; why were the p-values not adjusted? Please clarify and justify the p-value threshold.
Minor:
1. I am not sure enough if it was done to interrogate ; the word "randomisation" was used in this manuscript. However, the word "randomization" was used in most MR studies. Is it necessary to check the spelling?
2. The description of the MVMR method in the manuscript is Simplistic.
3. In the discussion part, "we found comparable effect sizes with previously published observational effects." Please cite some references to substantiate this sentence.
4. In the visualization part, is it necessary to show the life satisfaction and wellbeing spectrum results in Figure 5 and Figure 6? It might be more appropriate to consider them as secondary outcomes and explain them in the supplementary materials because of the lack of analysis.

Reviewer #3

(Remarks to the Author)
This is a very impressive manuscript examining relations between loneliness and social isolation with a wide array of health outcomes. The authors use a triangulation approach, comparing observational, within-family, MR effects to make causal inferences. The comprehensiveness of these analyses are a particular strength. Noting that I am not an expert in MR, the analyses seem to be well-executed and are quite impressive in their scope. The figures are also quite helpful in summarizing the results. I may even suggest an additional summary table; given how many results there are, it may be helpful for readers to be able to more quickly compare the effect sizes for a subset of key results (or perhaps aggregated across like results?).

My only other suggestion is for a more comprehensive Introduction. Has there been previous within-family work that is relevant? What might account for the previous mixed findings from MR? Is there a theoretical background for why loneliness versus social isolation may have causal impacts on health? Does this vary across different outcomes? I understand that space might be limited, but I think the very impressive set of results would be more impactful if couched within this context.

Version 1:

Reviewer comments:

Reviewer #1

(Remarks to the Author)
Thank you for the detailed response and I have no further comments.

(Remarks on code availability)

Reviewer #2

(Remarks to the Author)
Thanks for providing detailed clarifications. My concerns have been addressed in the revised submission. I have no further comments.

(Remarks on code availability)

Reviewer #3

(Remarks to the Author)
Thank you for your responses. I like the additions to the Introduction, and agree that a comprehensive table like the one you describe would not be practical. The thrust of my comment, however, was to highlight a subset results that the authors found most noteworthy, or perhaps an aggregated summary across methods or outcomes. Nonetheless, this is just a minor suggestion to make an already impressive manuscript a bit more easily digestible.

(Remarks on code availability)

Reviewer #4

(Remarks to the Author)
Review of Manuscript: Investigating causal relationships between loneliness, social isolation and health

Thank you for the opportunity to review this manuscript which examines the associations between loneliness, social isolation, and heath using data from UK Biobank. This is an important study: the topic is timely and public health relevant, the methodological approach is rigorous and robust, and the interpretation of the findings is presented with care. This paper is likely to have a significant impact on research in this area.

While the authors are transparent in addressing the limitations of their study, it would be helpful to further consider how these limitations may influence the findings. A primary concern relates to the choice of the sample for testing the causal associations between loneliness, social isolation and health. Although UK Biobank is a large cohort offering rich of data and research opportunities, it may not be optimal for testing causal effects of loneliness on health outcomes. Specifically:

Sample characteristics: UK Biobank participants are volunteers who tend to be white, older, and more educated than the general population in the UK. They also tend to follow healthy lifestyle. The authors should discuss how these sample characteristics may affect the generalisability and interpretation of the findings.

Measurement of loneliness: Loneliness was assessed at a single time point in adulthood using a single-item measure. The authors should consider how this limited assessment may constrain the conclusions drawn about the causal relationship between loneliness and health.

Age of participants: The mean age of participants is 56.45 years. Prior research indicates that loneliness can emerge early in life. A more comprehensive understanding of the effects of loneliness and social isolation would benefit from longitudinal studies that follow individuals from childhood through older adulthood, incorporating early-life assessments alongside later-life health outcomes. The authors should acknowledge this as a limitation.

For optimal clarity, the authors should avoid using acronyms whenever possible, and especially when referring to health outcomes.

(Remarks on code availability)

Version 2:

Reviewer comments:

Reviewer #4

(Remarks to the Author)
Thank you for considering my comments and addressing them in a revised version of the manuscript. This study is an important contribution to the field and I look forward to seeing it published. I have no further comments.

(Remarks on code availability)

Open Access This Peer Review File is licensed under a Creative Commons Attribution 4.0 International License, which permits use, sharing, adaptation, distribution and reproduction in any medium or format, as long as you give appropriate credit to the original author(s) and the source, provide a link to the Creative Commons license, and indicate if changes were made.
In cases where reviewers are anonymous, credit should be given to 'Anonymous Referee' and the source.
The images or other third party material in this Peer Review File are included in the article's Creative Commons license, unless indicated otherwise in a credit line to the material. If material is not included in the article's Creative Commons license and your intended use is not permitted by statutory regulation or exceeds the permitted use, you will need to obtain permission directly from the copyright holder.
To view a copy of this license, visit https://creativecommons.org/licenses/by/4.0/

Response to reviewer comments

Reviewer: 1

1. This is a study which utilized different designs to investigate the relation of loneliness and social isolation with different health outcomes. Whilst important, I feel that the novelty of the study is not very clearly delineated, especially there are already quite some Mendelian randomization studies conducted (amongst which also include UK Biobank). I am also concerned over the use of a social construct as an exposure in an MR. Please see below for my comments.

Response: We have addressed the specific comments below and hope that these changes now highlight the strength and novelty of our triangulation study. Whilst there are existing MR studies, some of which include UK Biobank, standalone MR studies can be biased. That is why we embedded our MR analyses within a comprehensive triangulation framework of other methods that rely on different biases and have different strengths. This included observational analyses adjusted for important potential confounders, sibling control analyses and both one-sample and two-sample MR analyses.

Taken together, our results across this combination of methods support stronger inference than a standalone MR study. The combination of 1) pre-registration of our proposed work, 2) the comprehensive nature of our analyses within a triangulation approach and 3) the range of outcomes examined in this study, goes significantly beyond existing studies. We also agree with the reviewer that using a social construct as an exposure in MR comes with some risk, which is why we decided to apply a unique triangulation approach and based our conclusions on results across these analyses rather than from MR analyses alone.

We have now added more discussion around the limitations of the MR analyses. We have responded further to each of these points where relevant below.

In addition, we have edited the Abstract, Introduction and Conclusions to highlight the novelty of our approach, as follows (we have also tracked changes in the manuscript):

Abstract: "This research extends and goes further than related work in this area by combining different methods, with different strengths and possible biases, in a pre-registered triangulation approach that examined a comprehensive range of outcomes. (Page 2)

Introduction: "Whilst there have been many previous observational and longitudinal studies in this area of research, there have been few MR studies, and even fewer studies that examine these relationships using MR alongside other approaches with different strengths and biases." (Page 4)

Introduction: "In the current study we have built on previous MR analyses, in particular the most recent aforementioned MR study by: 1) including both loneliness and social isolation

as exposures, 2) using more specific measures of these (previous measures have captured multiple related concepts making it difficult to disentangle specific effects), 3) including a wider number of relevant outcomes (e.g., including continuous measures and novel outcomes in this area such as rates of hospital admissions and quality-adjusted life years), 4) using a greater range of sensitivity analyses, and 5) conducting MR alongside other analyses to strengthen causal inference. These aspects are particularly important given that loneliness and social isolation are complex phenotypes and the genetic variants proxying for these may violate key assumptions of MR." (Page 4)

Introduction: "This approach extends previous research in this area by using a pre-registered triangulation approach, including a comprehensive number of sensitivity analyses and examining a range of outcomes." (Page 5)

Conclusions: "Our study used a combination of approaches, including some that were novel or had limited prior applications in this field (i.e., sibling control and MR analyses), to investigate whether loneliness and social isolation may causally influence a variety of general, physical and mental health and wellbeing outcomes." (Page 25)

2. I am personally less hesitant to consider social construct as a valid exposure for Mendelian randomziation design given these constructs are not as well defined and could be vulnerable to different degrees of horizontal pleiotropy compared to other biomarkers which are more likely to have a biological component, such as lipids and glucose. This somewhat echos with the point in the limitation (Line 431-434). This is particularly important especially when there are already quite some MRs being conducted in this area. Stronger justification would be helpful to highlight the novelty of the study.

Response: The reviewer raises an important point. To mitigate this potential bias, we designed a comprehensive triangulation approach, including not only MR but also extensive, confounder-adjusted regression analyses and sibling control analyses. We have now added further discussion of this to both the Introduction and the Discussion as detailed below. It is important to emphasize that this is not a standalone MR study, and the use of a triangulation framework is particularly important in cases like this where exposures are more biologically distal. Triangulation approaches including MR have been used to examine other relationships with social constructs (for example: van de Weijer et al, 2024 [1]). MR is a useful approach with orthogonal sources of bias to observational and other causal inference approaches, making it appropriate to consider as one of a series of approaches within a triangulation framework.

In addition, we have used a range of sensitivity analyses, some of which examine the presence of, and account for, horizontal pleiotropy. Many previous MR studies have not included such a comprehensive number of sensitivity analyses. By doing so we were able to better understand the results of our MR analyses. We also note that horizontal pleiotropy could still be having an impact despite the results of the sensitivity analyses, which is true of many MR studies and again highlights why it is important to use a triangulation approach

including MR. We have expanded on this point in the Discussion, as detailed below. None of the previous MR studies in this area use a triangulation approach like ours, and most use standalone MR. Furthermore, we do not make any causal conclusions from the MR analyses alone (as this is not advised for distal social outcomes) but instead we draw conclusions based on the overall body of evidence across the different study designs.

Given this, our triangulation study is an important addition to the literature as it goes beyond what previous studies have done and considers these important points raised.

Introduction: "However, caution should still be taken when interpreting MR results using exposures like loneliness and social isolation that are more biologically distal from genetic instruments, and where these are less well defined than more biological exposures would be. Therefore, it is important to consider findings from MR analyses alongside findings from other approaches." (Page 4)

Discussion: "We considered our results across the three different sets of analyses, as each analysis is prone to different limitations and biases. For example, the exposures in our MR analyses are not biological exposures and may be prone to horizontal pleiotropy (i.e., the instrument influencing the outcomes through pathways not via the exposure). In addition, some of our analyses (e.g., the one-sample MR analysis for loneliness and death) resulted in very imprecise estimates. A key strength of this study is the integration of multiple analyses through a triangulation approach, which allows for a comprehensive examination of each exposure-outcome relationship and enables the evaluation of conclusions based on the results from these diverse analyses." (Page 21)

And

Discussion: "MR analyses are likely to be more reliable when exposures are more directly related to the genetic variants included as instruments, for example, when using biological exposures such as blood-based measures [16]. Additionally, biologically distal exposures may increase the chances of there being horizontal pleiotropy. Although we conducted a range of sensitivity analyses to address this, it may be that pleiotropic pathways do still exist. This should be considered when interpreting the MR results and the MR results should also be interpreted alongside the other analyses within the triangulation framework." (Page 24)

3. Please explain in more detail why a new GWAS is needed for the social isolation instead of using a previously published one in UK Biobank (https://www.nature.com/articles/s41467-018-04930-1). I think a more useful approach is to use a family-MR design to address possible violations of independence assumption (https://www.nature.com/articles/s41467-020-17117-4).

Response: When we designed this study, we examined what existing social isolation GWAS were available and discussed these in depth. The study mentioned above was one of the ones we considered. However, we opted to conduct our own GWAS due to the nature of the measure used in the earlier study. The Day *et al* paper included GWAS for loneliness, social

isolation, social interactions and ability to confide. The social isolation GWAS used similar measures to us but combined these in a dichotomised variable (cases were defined as those who lived alone or did not see friends or family). That dichotomisation limited the continuous nature of the variables because there are levels to social isolation other than being completely socially isolated or not. One of the key issues in this field is that the measures are poorly defined [2], and we wanted to include exposures where we could allow as much detail as possible. In addition, better phenotyping reduces error, which deflates heritability. Therefore, better phenotypic measures with less measurement error will improve our genetic discovery in GWAS. The social interaction GWAS in the Day *et al* study was related to taking part in specific activities (sports club or gym; pub or social club; religious group). Such measurements are not useful to use because attending any of these groups could be picking up other behaviours e.g., physical activity, alcohol consumption etc as opposed to social interactions specifically. Therefore, we conducted our own GWAS for the specific exposure we thought would be the most informative. We have clarified this need for a new GWAS for social isolation in the Methods as follows:

"However, we conducted our own GWAS of social isolation in UK Biobank (see **Supplementary Materials Section 3, Supplementary Figure S1 and Supplementary Table S3**). There are GWAS of measures related to social isolation that already exist [23]. However, those typically focus on binary measures related to frequency of social contacts or on specific leisure activities. Therefore, to ensure as much information as possible was captured in the exposure, we conducted our own GWAS of the social isolation measure described above." (Page 27)

Whilst within-family MR can be conducted using sibling data from the UK Biobank, MR within sibships has been reported to be substantially less powerful than MR using unrelated individuals (often due to the much smaller sample sizes from restricting to siblings within a sample), including in work that members of this study's team conducted [3,1]. Given that, we opted to instead include sibling control analyses adjusting for key potential confounders. However, based on this comment we have conducted an initial analysis for this response to assess how well powered within-sibling MR would be for this sample. We have outlined this further in Appendix 1 (at the end of this document) in this response.

To summarise, given the split sample data we had available (which was used in the one-sample MR analyses and would also be required for within-sibling MR analyses) we found that within-sibling MR analyses for loneliness and mental health outcomes were substantially underpowered (we examined these relationships given these were where we had the strongest results across all analyses). As shown in the Appendix 1, Tables 1 and 2, the 95% confidence intervals were wide, and analyses too underpowered given the number of sibling pairs available in these samples to interpret these results. Based on this it was not suitable to include within-family MR analyses in the paper.

We have, however, discussed the potential to conduct within-family MR with larger samples in the *Future directions* section of the Discussion, as follows:

“Finally, further analyses that would complement this triangulation approach would be useful. For example, within-family MR would be helpful to better account for confounding due to factors such as population stratification and dynastic effects. We did not include this approach here as using the sibling data in UK Biobank would result in underpowered analyses [61], due to the small sample sizes we would have when combined with a split sample approach (similar to our one-sample approach, a split sample approach would be required given the sample overlap in the GWAS and UK Biobank). Further analyses using larger samples with data from mother, father, and child trios or sibling pairs may provide greater statistical power; however, the availability of such data to support this type of analysis remains limited.” (Page 25)

4. The bi-directional analyses would be difficult to interpret, especially for composite outcomes such as QALYs, let alone the issue of using binary traits (https://pubmed.ncbi.nlm.nih.gov/30039250/).

Response: The bidirectional analyses were only conducted where summary level GWAS data was available. Therefore, this did not include QALYs and other composite measures. We have clarified this in the Results as follows:

“These analyses were restricted to those where summary level GWAS data was available, similar to our other two-sample analyses (i.e., not including hospital admissions, QALYs, multimorbidity, death, self-harm, depression trait, anxiety trait and meaning in life).” (Page 19)

In cases where bidirectional relationships may be present, we agree this makes interpretability more difficult. We have conducted bidirectional MR analyses where possible to help investigate this. In addition, we have included MR methods such as Steiger filtering to help determine the likely direction of effect. In terms of binary exposures being more difficult to interpret we have added discussion of this to the Limitations section as follows:

“Fifth, in our two-sample MR analyses where we included binary exposures, there are limitations to interpreting these effects where these exposures may reflect dichotomisation of an underlying continuous trait e.g., a diagnosis of depression reflects a clinical cut-off of an underlying continuous distribution of depressive symptoms [61]. The main limitation of including binary exposures is that the effect estimate may not be particularly interpretable, Therefore, results from MR analyses with binary exposures in this study should be considered in terms of direction of effect as opposed to overly focusing on the magnitude of the effect.” (Page 24)

We also clarify that we are interested in examining whether there might be bidirectional effects as opposed to the magnitude of these effects as follows in the Results:

“We were interested in whether there was evidence to suggest there may also be effects operating in the opposite direction, but we were not focused on interpreting the magnitudes of these effects.” (Page 19)

Line 178: Please clarify the meaning of complexity of the model when discussing results from the Cox model.

Response: We have clarified this point in the manuscript. The complexity we referred to previously is in fact the reduced power of MR (and any instrumental variable) analyses compared to most other methods applied to similar sample sizes. This is more pronounced in 1SMR because sample sizes were smaller in these analyses. Furthermore, the power of any Cox proportional hazards model is related to the number of events. These factors combine to give particularly imprecise estimates for 1SMR analyses of these rarer survival-time outcomes. We have edited the text to read as follows:

"We note that the Cox proportional hazards models used for the 1SMR where death was the outcome were likely too imprecise to reliably assess evidence of an effect. MR analyses are less powerful than other methods applied to the same sample size. This is exacerbated by the reduced statistical power of a Cox model when the event is rare. Where we do not see evidence of effects, confidence intervals were wide so should not be interpreted as evidence of absence of an effect." (Page 9)

And

"We note that the Cox proportional hazards models used for the 1SMR, where heart failure, CAD and stroke were outcomes, were also likely too imprecise to reliably assess evidence of an effect. MR analyses are less powerful than other methods applied to the same sample size, and this reduced statistical power in our 1SMR analyses was not mitigated by the large sample sizes in the GWAS used for the 2SMR. This is exacerbated by the reduced statistical power of a Cox model when the event is rare. Where we do not see evidence of effects, confidence intervals were wide so should not be interpreted as evidence of absence of an effect." (Page 12)

5. Whilst I agree that we should not only rely on statistical significance to determine causation, some estimates were very imprecise, such as the MR estimate for death, making these estimates nearly impossible to make any valid inference.

Response: We agree with this point and in particular note this is the case mainly for 1SMR analyses. The triangulation approach is useful for this reason. We tried to highlight this point in the previous version of the paper as well. We have edited the language to ensure that when referring to MR analyses alone we do not refer to these as causal effects. Given that, for death, for example, the other analyses have much narrower CIs and do suggest an effect, we are hesitant to rule an effect out because of imprecise 1SMR estimates, hence our mentioning the following in the results: "Additionally, where we do not see evidence of effects, confidence intervals were wide so should not be interpreted as evidence of absence of an effect." This is also useful to highlight further in the Discussion and therefore we have added the following text:

“We considered our results across the three different sets of analyses, as each analysis is prone to different limitations and biases. For example, the exposures in our MR analyses are not biological exposures and may be prone to horizontal pleiotropy (i.e., the instrument influencing the outcomes through pathways not via the exposure). In addition, some of our analyses (e.g., the one-sample MR analysis for loneliness and death) resulted in very imprecise estimates. A key strength of this study is the integration of multiple analyses through a triangulation approach, which allows for a comprehensive examination of each exposure-outcome relationship and enables the evaluation of conclusions based on the results from these diverse analyses.” (Page 21)

6. I am not sure if correction for multiple testing is present. Please clarify.

Response: We did not correct for multiple testing because our triangulation approach was primarily focused on assessing whether there was evidence across analyses supporting potential causal effects. We sought to avoid arbitrary dichotomisation based on specific p-value thresholds by assessing the evidence comprehensively across primary analyses, sensitivity analyses, and through consideration of estimate precision.

Therefore, we did not feel that adjusting for multiple testing would be appropriate and in fact may be detrimental to the interpretation of these results. For this reason, we also prefer to avoid describing results as “statistically significant” (see Sterne and Davey Smith, 2001 https://www.bmj.com/content/322/7280/226.1); we instead discuss our results in the context of the overall body of evidence they provide. Even in the absence of these considerations, the multiple testing burden across these analyses is likely to be low.

We have added further detail of this approach to the Methods as follows:

“Our triangulation approach was qualitative (i.e., assessing whether there was consistent evidence of potential causal effects). Therefore, we have not focused on significance thresholds and have not adjusted for multiple testing. We have instead considered evidence across the main analyses, sensitivity analyses and have also considered the precision of estimates when interpreting results.” (Page 27)

7. Line 149-150: Beta, 95%CI for phenotypic correlation are the same.

Response: These are correct after rounding to 2 decimal places. The beta was 0.1304713 and the 95% CI was 0.1274456 to 0.1334946 prior to rounding.

8. Line 170: Not sure why a p value of 0.83 was mentioned

Response: We included this to try and summarise the range of p-values across analyses. We have now amended the text to make this clearer as follows:

“For death and hospital admissions, there were associations between being lonely and an increased hazard for death across all approaches, except MR ($p \leq 0.001$ to 0.006) as well as increased rates of hospital admissions in the observational and sibling control analyses for

the between-family effect only ($p \leq 0.001$ to 0.04); the corresponding MR estimates were not inconsistent but were very imprecise (**Figure 1**). We also found associations between increased social isolation and an increased hazard for death in the observational and the sibling control analyses for the within-family effect only ($p \leq 0.001$); again, the corresponding MR estimate was not inconsistent but was imprecise (**Figure 2**).” (Page 8)

9. Can the authors add references to the 2nd and 3rd paragraph of the Discussion? This would help readers to understand their interpretation in the context of other evidence.

Response: We have added references to these paragraphs as highlighted below.

3rd paragraph: “We found comparable effect sizes with previously published observational effects e.g., for depression and death [7,55].” (Page 22)

4th paragraph: “Our findings may reflect an absence of evidence as opposed to evidence of absence, particularly given previous findings in longitudinal studies suggesting effects of loneliness and social isolation on physical health outcomes, including cardiovascular disease and T2D [3,14].” (Page 22)

4th paragraph: Third, we examined only a select number of physical health outcomes, and it may be that there are effects on other physical health outcomes not examined here (i.e., non-cardiovascular outcomes). Previous studies have found effects on other physical health outcomes that we did not examine, such as dementia, Alzheimer’s disease, asthma and osteoarthritis, amongst others [4,56,57], and these merit examination in future triangulation studies. Outcomes examined in this study were chosen for being particularly burdensome health outcomes with previously observed associations with loneliness and/or social isolation [1,7,12,38,40,44–46,48,51]. (Page 22)

10. Why is mean difference presented for dichotomous outcomes (e.g. Table S12)?

Response: We apologise for this oversight. For the one-sample MR sensitivity analyses we used a different approach to the main one-sample MR analyses. In order to conduct weighted median and weighted mode sensitivity analyses a two-sample MR approach was used. As the sample was split into two, in order to obtain overall estimates for these analyses the results were meta-analysed using the metagen() function in R. These results should have been reported as the log of the odds ratio/hazards ratio and not the mean difference for binary outcomes. However, we have now converted these to odds ratios and hazards ratios to be more interpretable and amended the column header in Supplementary Table S12 to reflect this.

Reviewer: 2

1. The authors completed the STROBE-MR checklist and described the three core assumptions underlying MR in the manuscript. In the observational and sibling control analysis, the author identified multiple confounders. However, those confounders were not

mentioned or adjusted in the MR part. Does this study design violate the core assumptions of Mendelian randomization?

Response: This does not violate a core assumption of Mendelian randomisation. The assumption around confounders in MR is that there is no confounding of the genetic variants and the outcome. This assumption specifically refers to issues around dynastic effects, population structure or stratification etc. One approach to avoid violating this assumption is to include individuals from the same population. Therefore, in our one-sample MR analyses we included individuals of European ancestry only. This assumption does not refer to confounders such as those included in the observational and sibling control analyses. Any variables like those we included as potential confounders in the observational and sibling control analyses would have occurred post-conception and therefore would be considered as an intermediate variable in the MR analysis and should not be adjusted for to avoid collider bias. We adjusted for these variables in observational and sibling control analyses as this is not an issue in those analyses and these were considered important potential confounders. We have clarified why we do not include these in the MR analyses as follows:

"We do not adjust for these in our MR analyses as this would be inappropriate given that these potential confounders occur post-conception (and therefore cannot influence genetic variants) and the assumption in MR related to confounding refers to there being no confounding of the genetic variants and outcomes." (Page 28)

2. In the MR analysis part, the authors calculated F-statistics to estimate the strength of the instruments. This is a normal step for MR. However, I can not find any threshold that was determined and used. I think the threshold is necessary. What's more, some of the F-statistics shown in Table S13 were as low as 3.49. Do these IVs really meet the analysis requirements?

Response: We have tried to avoid using an arbitrary dichotomisation e.g., using a threshold of 10, in this study. We have instead considered instrument strength in a continuous way [4]. Whilst a threshold of F-statistic > 10 is a useful guide [5], we caution against interpreting values above or below this value as being indicative of "strong" or "weak" instruments. Instead, low values are likely to indicate weak instrument bias and results should be interpreted as accounting for this. Where life satisfaction was the exposure, i.e., in the bidirectional analyses, the instrument had an F-statistic of 3.49, this was much lower than other values and may suggest that the instrument is weak. We did find evidence of effects where life satisfaction was the exposure for both loneliness and social isolation and had therefore not commented on this in the main paper before. However, we have now added this to the relevant Results section (we have also tracked changes in the manuscript) and highlighted how the F-statistic was evaluated in the Supplementary Materials, as follows:

"Where life satisfaction was the exposure the F-statistic was 3.49, which likely indicates the presence of weak instrument bias [18]. Given this, the findings for life satisfaction should be interpreted with caution." (Page 19)

And

"Again, given the F-statistic of 3.49 for life satisfaction, the results likely indicate the presence of weak instrument bias, and caution should be taken when interpreting them." (Page 19)

And in the Supplementary Materials: "To estimate the strength of our instruments we calculated first stage F-statistics, where a low value may indicate the presence of weak instruments that could bias results [12,17]." (Page 7)

3. This study conducted a large-scale Mendelian randomization analysis; why were the p-values not adjusted? Please clarify and justify the p-value threshold.

Response: As mentioned in response to Reviewer 1's comment around multiple testing, we did not correct for multiple testing as in our triangulation approach as we were primarily focused on assessing whether there was evidence across multiple different methodological approaches supporting potential causal effects (i.e., consistent evidence of effects across all analyses would be considered as a basis for inferring a causal effect). In addition, prior to analyses, based on previous literature we were already expecting there to be an association (although not necessarily causal) between loneliness and the health outcomes. Therefore, correcting for multiple testing would like lead to overcorrecting given existing knowledge. We sought to avoid arbitrary dichotomisation based on specific p-value thresholds by assessing the evidence comprehensively across primary analyses, sensitivity analyses, and through consideration of estimate precision.

Therefore, we did not feel that adjusting for multiple testing would be appropriate and in fact may be detrimental to the interpretation of these results. For this reason, we also prefer to avoid describing results as "statistically significant" (see Sterne and Davey Smith, 2001 https://www.bmj.com/content/322/7280/226.1); we instead discuss our results in the context of the overall body of evidence they provide. Even in the absence of these considerations, the multiple testing burden across these analyses is likely to be low.

We have added further detail of this approach to the Methods as follows:

"Our triangulation approach was qualitative i.e., assessing whether there was consistent evidence of potential causal effects. Therefore, we have not focused on significance thresholds and have not adjusted for multiple testing. We have instead considered evidence across the main analyses, sensitivity analyses and have also considered the precision of estimates when interpreting results." (Page 27)

4. I am not sure enough if it was done to interrogate ; the word "randomisation" was used in this manuscript. However, the word "randomization" was used in most MR studies. Is it necessary to check the spelling?

Response: Both randomisation and randomization have both been used in previous MR studies. We will defer to the Editor to decide which version should be used.

5. The description of the MVMR method in the manuscript is Simplistic.

Response: We have added more details to both the Results and Methods sections, as well as the Supplementary Materials, where MVMR is mentioned, as follows:

Results: "We conducted multivariable MR (MVMR) analyses with both loneliness and social isolation as exposures to assess whether their influences on outcomes were independent (**Supplementary Table S16**). MVMR allows for the inclusion of multiple exposures to estimate the causal effect of each exposure conditioned on the other. Results supported 2SMR findings for increased loneliness on increased risk of suicide attempt, depression and decreased levels of wellbeing spectrum, positive affect and life satisfaction. These results were consistent across sensitivity analyses, with the exception of MR-Egger, where confidence intervals were wide (**Supplementary Table S16**). This suggests that social isolation did not impact the influence of loneliness on these outcomes." (Page 19)

Methods: "We additionally conducted multivariable MR (MVMR) [78] analyses, using the 'MVMR' package in R, to estimate independent effects of loneliness and social isolation on health outcomes. MVMR estimates the direct effect of multiple exposures on an outcome in a single estimation and is not biased by the inclusion of genetic variants associated with many/all of the exposures in the model. Genetic instruments for both exposures were included to allow the effect of loneliness and social isolation on the outcome to be estimated, whilst conditioning on the other. Sensitivity analyses to detect and correct for potential horizontal pleiotropy were also included (see **Supplementary Materials Section 9** for further details)." (Page 31)

Supplementary Materials Section 9: "MVMR is an extension of MR that can be used to estimate the potential causal effects of multiple exposures (here loneliness and social isolation) on an outcome and assess whether effects of each exposure (conditioning on the other) are independent. In order to do this data for associations of each genetic variant in the instrument with each of the exposures was required. Analyses were conducted using the 'MVMR' package in R [18] using a 2SMR approach. Conditional F-statistics were also calculated with this R package. The main MVMR analysis was the IVW approach. We also conducted sensitivity analyses which included MR Egger, oriented on each exposure (i.e., ensuring the betas are positive for that exposure) to correct for measured and unmeasured horizontal pleiotropy and MR-PRESSO (raw and outlier corrected) to detect outliers and correct for horizontal pleiotropy via removal of outliers. MR PRESSO also included the global and distortion tests, similar to in the main two-sample MR analyses. Finally, we included a pleiotropy test which measures SNP heterogeneity, which can indicate pleiotropy." (Page 8)

6. In the discussion part, "we found comparable effect sizes with previously published observational effects." Please cite some references to substantiate this sentence.

Response: We have added references (7 and 55) to support this statement, as follows:

“We found comparable effect sizes with previously published observational effects (e.g., for depression and death) [7,55].” (Page 21)

The references added are:

7. Mann, F. et al. Loneliness and the onset of new mental health problems in the general population. Soc Psychiatry Psychiatr Epidemiol 57, 2161–2178 (2022).

55. Holt-Lunstad, J., Smith, T. B., Baker, M., Harris, T. & Stephenson, D. Loneliness and Social Isolation as Risk Factors for Mortality: A Meta-Analytic Review. Perspectives on Psychological Science 10, 227–237 (2015).

7. In the visualization part, is it necessary to show the life satisfaction and wellbeing spectrum results in Figure 5 and Figure 6? It might be more appropriate to consider them as secondary outcomes and explain them in the supplementary materials because of the lack of analysis.

Response: We are inclined to retain these in the current Figures to allow these results to be included alongside others in the main manuscript. We are happy to consider moving these under the advice of the Editor if preferred though.

Reviewer: 3

1. This is a very impressive manuscript examining relations between loneliness and social isolation with a wide array of health outcomes. The authors use a triangulation approach, comparing observational, within-family, MR effects to make causal inferences. The comprehensiveness of these analyses are a particular strength. Noting that I am not an expert in MR, the analyses seem to be well-executed and are quite impressive in their scope. The figures are also quite helpful in summarizing the results. I may even suggest an additional summary table; given how many results there are, it may be helpful for readers to be able to more quickly compare the effect sizes for a subset of key results (or perhaps aggregated across like results?).

Response: We thank the reviewer for their positive comment. We agree that a summary table of the results would be useful for readers alongside the figures. However, given the number of outcomes (19 in total) and the number of methods this would result in a table that is about 3 pages long with both exposures (if exposure-outcome relationships were the rows and the methods were the columns). We opted to include figures to display the main results which bring together all results and allow for easy comparison of results across outcomes and methods. We include full results in the supplementary materials for readers if they want to see specific details.

We can include this if the Editor would prefer the results to also be presented in a table despite the size this would be.

2. My only other suggestion is for a more comprehensive Introduction. Has there been

previous within-family work that is relevant? What might account for the previous mixed findings from MR? Is there a theoretical background for why loneliness versus social isolation may have causal impacts on health? Does this vary across different outcomes? I understand that space might be limited, but I think the very impressive set of results would be more impactful if couched within this context.

Response: We have added further details to the Introduction as suggested. First, we have included some background on within-family studies in this area, noting that there is generally a lack of studies using this approach and none that are similar to our analyses:

"Within-family analyses can also help to estimate effects of an exposure on an outcome, whilst controlling for familial confounding and shared genetic predispositions. This approach requires the use of cohort data where there are siblings or other family members. Given this limitation there are few studies using within-family approaches to investigate relationships between loneliness/social isolation and health [28-30]. Of those that do exist they are focused on specific health-related outcomes and there are no studies, to our knowledge, looking at a range of health outcomes using within-family approaches." (Page 5)

Second, we have included more discussion around the previous mixed findings in MR studies, as follows:

"Other MR studies have not found evidence for causal effects of loneliness/social isolation on both physical and mental health outcomes [23-25], reflecting mixed findings in this area. These mixed MR findings may be a result of differences in the exact exposures used (i.e., examining leisure activities [22] versus frequency of social contacts [26] with both being described as social isolation measures, or combining measures of loneliness and social isolation into a single measure making it difficult to disentangle specific effects [23,25]) or differences in the approaches taken. For example, some previous studies have used a wide range of sensitivity analyses and found less consistent results across these suggesting a lack of evidence of effects [24,27], whilst others have included minimal sensitivity analyses [25], or have focused largely on results from the main analyses [20,22]. This has meant the way in which results have been interpreted has varied. In addition, many of those studies have further limitations in terms of using less stringent p-value thresholds for instrument selection [20,22], not including appropriate sensitivity analyses or not interpreting results appropriately in light of these [20,22,25]." (Page 4)

Third, in relation to the theoretical background on loneliness versus social isolation we note that there are few studies that have examined both loneliness and social isolation, so it is difficult to know whether they have different effects on health outcomes. Where they have been studied together (e.g., Christiansen et al., 2021[6]), they have impacted the same physical health outcomes (albeit to different extents) but appeared to do that via different pathways. For example, psychological factors (e.g., stress) predominantly explained the effects of loneliness on health outcomes, whereas behavioural factors (e.g., smoking and inactivity) were more important for SI. Those findings suggest that pathways through which loneliness and social isolation influence health outcomes might depend on the specific outcome.

We have added the following text to the introduction:

“We note that there are few studies that have examined the effects of both loneliness and social isolation on health and therefore it is difficult to disentangle whether they have different effects on health outcomes. Where these have been studied together [3] they have influenced similar outcomes, although often to different extents [1]. However, the nature of these relationships may be different and there is a need to investigate whether influences of loneliness and social isolation on health outcomes are independent.” (Page 3)

References:

1. van de Weijer MP, Demange PA, Pelt DHM, Bartels M, Nivard MG. Disentangling potential causal effects of educational duration on well-being, and mental and physical health outcomes. Psychol Med. 2024 May;54(7):1403-1418. doi: 10.1017/S003329172300329X. Epub 2023 Nov 15. PMID: 37964430.

2. Lim MH, Qualter P, Ding D, Holt-Lunstad J, Mikton C, Smith BJ. Advancing loneliness and social isolation as global health challenges: taking three priority actions. Public Health Res Pract. 2023 Sep 13;33(3):3332320. doi: 10.17061/phrp3332320. PMID: 37699761.

3. Brumpton B, Sanderson E, Heilbron K, Hartwig FP, Harrison S, Vie GÅ, Cho Y, Howe LD, Hughes A, Boomsma DI, Havdahl A, Hopper J, Neale M, Nivard MG, Pedersen NL, Reynolds CA, Tucker-Drob EM, Grotzinger A, Howe L, Morris T, Li S; Within-family Consortium; 23andMe Research Team; Auton A, Windmeijer F, Chen WM, Bjørngaard JH, Hveem K, Willer C, Evans DM, Kaprio J, Davey Smith G, Åsvold BO, Hemani G, Davies NM. Avoiding dynastic, assortative mating, and population stratification biases in Mendelian randomization through within-family analyses. Nat Commun. 2020 Jul 14;11(1):3519. doi: 10.1038/s41467-020-17117-4. PMID: 32665587; PMCID: PMC7360778.

4. Lawlor DA, Harbord RM, Sterne JA, Timpson N, Davey Smith G. Mendelian randomization: using genes as instruments for making causal inferences in epidemiology. Stat Med. 2008 Apr 15;27(8):1133-63. doi: 10.1002/sim.3034. PMID: 17886233.

5. Burgess S, Thompson SG; CRP CHD Genetics Collaboration. Avoiding bias from weak instruments in Mendelian randomization studies. Int J Epidemiol. 2011 Jun;40(3):755-64. doi: 10.1093/ije/dyr036. Epub 2011 Mar 16. PMID: 21414999.

6. Christiansen J, Lund R, Qualter P, Andersen CM, Pedersen SS, Lasgaard M. Loneliness, Social Isolation, and Chronic Disease Outcomes. Ann Behav Med. 2021 Mar 20;55(3):203-215. doi: 10.1093/abm/kaaa044. PMID: 32865550.

Appendix 1

We conducted within-sibling MR analyses in response to Reviewer 1's comment 3. Our analyses indicated that the within-sibling MR approach is underpowered. This is not surprising given the wide confidence intervals observed for some of the one sample MR analyses and that the sample size was further limited as a split sample approach within siblings was needed.

We conducted these analyses for loneliness and mental health outcomes only, as this is where we observed the strongest results across the other analyses, thus serving as a useful example for exploring this approach. As demonstrated in Tables 1 and 2 below (each table presents results for one of the split samples), the analyses were very underpowered as evidenced by the large estimates, wide confidence intervals and corresponding large p-values.

We did not conduct further analyses for the other outcomes, and we have not included this approach within the revised paper, given these were underpowered.

We would also like to highlight the very small sample sizes for some of these outcomes. The sample sizes could be increased slightly as we used the split samples generated for the previous one-sample MR analyses, which did not account for sibships and therefore the sample size was further limited. However, this is not feasible and given the below results this is unlikely to yield informative results.

Table 1. Within-sibling MR analyses for split sample 1 examining the relationship between loneliness and mental health outcomes

Outcome	N	Estimate	Lower CI	Upper CI	p-value
Self harm	3,366	1.30	-13.06	15.66	0.86
Suicide attempt	3,361	0.18	-7.32	7.68	0.96
Depression diagnosis	10,248	0.61	-1.34	2.57	0.54
Anxiety diagnosis	10,248	-0.11	-1.67	1.45	0.89
Depression trait	3,321	33.71	-295.10	362.52	0.84
Anxiety trait	3,334	1620.48	-628,369.68	631,610.65	1.00
Positive affect / happiness	5,394	-1.50	-4.24	1.24	0.28
Meaning in life	3,305	-8.10	-64.83	48.64	0.78

CI=95% confidence interval

Table 2. Within-sibling MR analyses for split sample 2 examining the relationship between loneliness and mental health outcomes

Outcome	N	Estimate	Lower CI	Upper CI	p-value
Self harm	3,277	0.22	-2.30	2.74	0.87
Suicide attempt	3,273	0.15	-1.65	1.95	0.87

Depression diagnosis	10,002	0.70	-1.62	3.03	0.55
Anxiety diagnosis	10,002	-0.25	-2.12	1.62	0.79
Depression trait	3,226	-24.78	-158.15	108.59	0.72
Anxiety trait	3,242	-35.14	-214.85	144.56	0.70
Positive affect / happiness	5,215	0.83	-4.25	5.92	0.75
Meaning in life	3,213	7.62	-22.83	38.07	0.62

CI=95% confidence interval

Response to reviewer comments

Reviewer: 1

1. Thank you for the detailed response and I have no further comments.

Response: We thank the reviewer for their time and comment.

Reviewer: 2

1. Thanks for providing detailed clarifications. My concerns have been addressed in the revised submission. I have no further comments.

Response: We thank the reviewer for their time and comment.

Reviewer: 3

1. Thank you for your responses. I like the additions to the Introduction, and agree that a comprehensive table like the one you describe would not be practical. The thrust of my comment, however, was to highlight a subset results that the authors found most noteworthy, or perhaps an aggregated summary across methods or outcomes. Nonetheless, this is just a minor suggestion to make an already impressive manuscript a bit more easily digestible.

Response: We thank the reviewer for their positive comments. We have not highlighted a subset of results as we have presented results carefully to avoid dichotomisation based on p-values, and to avoid interpreting an absence of evidence as evidence of absence of an effect. We do agree that a summary of our findings would be useful at the start of the results, so we have added this, before describing the results in detail, as follows:

"To summarise, we found evidence to suggest causal effects of loneliness and social isolation on poorer mental health and wellbeing and of loneliness on poorer general health, with stronger effects for loneliness compared to social isolation in our results. We did not find evidence of effects of loneliness and social isolation on physical health outcomes, although we were unable to rule out causal relationships here." (Page 9)

Reviewer: 4

1. Thank you for the opportunity to review this manuscript which examines the associations between loneliness, social isolation, and heath using data from UK Biobank. This is an important study: the topic is timely and public health relevant, the methodological approach is rigorous and robust, and the interpretation of the findings is presented with care. This paper is likely to have a significant impact on research in this area.

While the authors are transparent in addressing the limitations of their study, it would be helpful to further consider how these limitations may influence the findings. A primary concern relates to the choice of the sample for testing the causal associations between loneliness, social isolation and health. Although UK Biobank is a large cohort offering rich of data and research opportunities, it may not be optimal for testing causal effects of loneliness on health outcomes. Specifically:

Sample characteristics: UK Biobank participants are volunteers who tend to be white, older, and more educated than the general population in the UK. They also tend to follow healthy lifestyle. The authors should discuss how these sample characteristics may affect the generalisability and interpretation of the findings.

Measurement of loneliness: Loneliness was assessed at a single time point in adulthood using a single-item measure. The authors should consider how this limited assessment may constrain the conclusions drawn about the causal relationship between loneliness and health.

Age of participants: The mean age of participants is 56.45 years. Prior research indicates that loneliness can emerge early in life. A more comprehensive understanding of the effects of loneliness and social isolation would benefit from longitudinal studies that follow individuals from childhood through older adulthood, incorporating early-life assessments alongside later-life health outcomes. The authors should acknowledge this as a limitation.

Response: We thank the reviewer for their comments. We agree that these study limitations should be more clearly highlighted, and that the findings should be interpreted in light of them. We have addressed each of the points as outlined below.

Sample characteristics: We already included reference to UK Biobank participants being healthier and less socioeconomically deprived in our limitations section. We have expanded on this point to further highlight how our results could be impacted by that limitation (strengths and limitations section, page 27):

"In terms of representation, UK Biobank participants are generally healthier and less socioeconomically deprived than the general population [60] and our MR analyses were restricted to individuals of European ancestry. Therefore, our results may not be generalisable outside of those populations. In addition, this may mean that there are fewer cases in terms of the health outcomes examined, continuous measures may be skewed towards 'healthier' ranges, and rates of loneliness and social isolation may be lower than in the general population. Individuals with poorer health or who are more lonely or socially isolated may have been less likely to participate in UK Biobank, introducing selection bias in our analyses which could bias associations. Therefore, caution should be taken when interpreting results from analyses based solely on UK Biobank data."

Measurement of loneliness: We noted already that we only used responses to a single question around loneliness but have expanded on this in the strengths and limitations section (Page 26; see below):

"Second, our measure of loneliness in UK Biobank was a binary response to a single question asking whether the respondent was lonely, whereas other studies have used composite measures of loneliness. Loneliness is likely to be captured better using such validated composite measures that include different aspects of loneliness. The single item measure we have used could result in weaker associations in this study."

Age of participants: This is an important limitation of the study, and we have added this to the strengths and limitations section of the discussion as follows:

"Furthermore, our measures of both loneliness and social isolation were assessed at a single time point during middle-late adulthood. Both loneliness and social isolation are likely to change across the life course, and important time periods for loneliness and social isolation e.g., childhood, adolescence and young adulthood are not captured in this study. It may be that effects on the health outcomes studied here vary across different life stages and therefore conclusions about the impact of loneliness and social isolation in earlier life are limited. However, even a single measurement of loneliness is likely to reflect chronic loneliness in some individuals. In addition, by including only a single timepoint we were able to able to examine outcomes that temporally occurred after the exposure was measured. We did include MR analyses which should provide estimates of effects over the lifetime, but these results would be strengthened by the inclusion of analyses in age groups not covered in UK Biobank." (Page 27)

We have also added how this would be important to examine further in future studies within the Future directions section, as follows (Page 28):

"Examining the impact of loneliness and social isolation across the life course would provide additional insight, but this was not possible in the current study given the data available. Longitudinal studies (including chronic loneliness and social isolation) would be useful to provide a more comprehensive understanding of the impact of these exposures on health outcomes."

2. For optimal clarity, the authors should avoid using acronyms whenever possible, and especially when referring to health outcomes.

Response: We agree that it would be more helpful for readers to have these acronyms spelt out. We have removed the health outcome acronyms from the main text (with the

exception of QALYs), although have kept these in tables and figures with footnotes defining them in order to save space.
